# Effects of Probiotics on Preterm Infant Gut Microbiota Across Populations: A Systematic Review and Meta-Analysis

**DOI:** 10.1016/j.advnut.2024.100233

**Published:** 2024-05-20

**Authors:** Pandi He, Leilei Yu, Fengwei Tian, Wei Chen, Hao Zhang, Qixiao Zhai

**Affiliations:** 1 State Key Laboratory of Food Science and Resources, Jiangnan University, Wuxi, Jiangsu, China; 2 School of Food Science and Technology, Jiangnan University, Wuxi, Jiangsu, China; 3 National Engineering Research Center for Functional Food, Jiangnan University, Wuxi, Jiangsu, China

**Keywords:** meta-analysis, probiotics, preterm infants, gut microbiota, MetaCyc functional pathways

## Abstract

Microbiota in early life is closely associated with the health of infants, especially premature ones. Probiotics are important drivers of gut microbiota development in preterm infants; however, there is no consensus regarding the characteristics of specific microbiota in preterm infants receiving probiotics. In this study, we performed a meta-analysis of 5 microbiome data sets (1816 stool samples from 706 preterm infants) to compare the gut microbiota of preterm infants exposed to probiotics with that of preterm infants not exposed to probiotics across populations. Despite study-specific variations, we found consistent differences in gut microbial composition and predicted functional pathways between the control and probiotic groups across different cohorts of preterm infants. The enrichment of *Acinetobacter*, *Bifidobacterium*, and *Lactobacillus* spp and the depletion of the potentially pathogenic bacteria *Finegoldia*, *Veillonella*, and *Klebsiella* spp. were the most consistent changes in the gut microbiota of preterm infants supplemented with probiotics. Probiotics drove microbiome transition into multiple preterm gut community types, and notably, preterm gut community type 3 had the highest α-diversity, with enrichment of *Bifidobacterium* and *Bacteroides* spp. At the functional level, the major predicted microbial pathways involved in peptidoglycan biosynthesis consistently increased in preterm infants supplemented with probiotics; in contrast, the crucial pathways associated with heme biosynthesis consistently decreased. Interestingly, *Bifidobacterium* sp. rather than *Lactobacillus* sp. gradually became dominant in gut microbiota of preterm infants using mixed probiotics, although both probiotic strains were administered at the same dosage. Taken together, our meta-analysis suggests that probiotics contribute to reshaping the microbial ecosystem of preterm infants at both the taxonomic and functional levels of the bacterial community. More standardized and relevant studies may contribute to better understanding the crosstalk among probiotics, the gut microbiota, and subsequent disease risk, which could help to give timely nutritional feeding guidance to preterm infants.

This systematic review and meta-analysis was registered at PROSPERO (https://www.crd.york.ac.uk/PROSPERO/) as CRD42023447901.

**Statement of Significance**

Although there are systematic reviews of probiotics and clinical outcomes in preterm infants, gut microbiota has not yet been taken into account in the analyses. To our knowledge, this is the first report to comprehensively examine the association between probiotics and gut microbiota in preterm infants.

## Introduction

Preterm is defined as infants born alive before 37 wk of gestation. Approximately 15 million preterm births occur per annum, accounting for 1 in 9 births worldwide [1], and ∼1 million of these children die owing to complications of preterm birth [2]. The establishment of the gut microbiota in preterm infants is delayed compared with that in full-term infants, which is partially attributed to cesarean delivery, receipt of antibiotics, and the special environment of neonatal intensive care units [3,4]. In infants delivered vaginally at term, facultative anaerobic bacteria (such as *Streptococcus* and *Staphylococcus* spp.) predominate in the early stages, followed by Bacteroides-dominated and Bifidobacterium-dominated communities [5,6]. The preterm gut microbiome is characterized by decreased α-diversity, delayed colonization by obligate anaerobic bacteria, and enrichment of potentially pathogenic bacteria [[7], [8], [9]], following somewhat conserved microbial colonization patterns [10,11]. Altered gut microbial ecosystems and lower diversity are positively related to risk of serious clinical complications such as necrotizing enterocolitis (NEC) [12], late-onset sepsis (LOS) [13], and asthma [14].

Interventions to “normalize” the preterm gut microbiome are promising for improving health and preventing diseases in preterm infants. Supplementation with infant commensal bacteria in the form of probiotic supplements appears to be a viable method to promote the colonization of the gut of preterm infants by beneficial early-life microbiota members [15]. It has recently been reported that probiotics are important drivers of gut microbiome development in preterm infants [16]. Meta-analyses have indicated that probiotics contribute to reducing NEC, sepsis, and all-cause mortality in preterm infants [[17], [18], [19]]. We hypothesized that the well-known benefits of probiotics on clinical outcomes in preterm infants may be partially attributable to their effects on the gut microbiota. Recently, several studies have suggested that probiotics exert health benefits by regulating the gut microbiota in preterm infants [[20], [21], [22]]. Heterogeneity in probiotic species and doses, microbiome data processing, and analytical approaches lead to difficulties in synthesizing and interpreting these results.

Until now, meta-analyses on the role of probiotics on clinical outcomes in premature infants have been conducted, including all-cause mortality [[17], [18], [19]], NEC [[17], [18], [19]], LOS [[17], [18], [19]], retinopathy of prematurity [23], bronchopulmonary dysplasia [24], and neurodevelopment [25]; however, none of the studies evaluated the impacts of probiotics on the development of the preterm gut microbiota. In this study, we describe findings from a large-scale meta-analysis of 16S rRNA gene amplicon sequencing to explore the association between probiotics and the development of gut microbiota in preterm infants and to better understand the crosstalk among probiotics, gut microbiota, and subsequent disease risk.

## Methods

This systematic review and meta-analysis has been registered in the PROSPERO registry (CRD42023447901).

### Search strategy

A systematic search of articles published through November 22, 2023, was performed in Web of Science, PubMed, Cochrane, and EMBASE. To optimize the search results, keywords and their synonyms were used, combined with the Boolean phrases “AND” intergroup and “OR” intragroup. No restrictions were imposed on the year of publication. The reference lists of all relevant articles and reviews were manually searched. The detailed search strategies are presented in Supplemental Table 1.

### Inclusion and exclusion criteria

The inclusion criteria were as follows: *1*) intervention or observational studies (randomized controlled trials or cohort studies); *2*) administration of probiotics as the exclusive intervention or exposure to probiotic supplements; and *3*) collection of gut microbiome results from preterm infants. The exclusion criteria were as follows: *1*) inappropriate research types (reviews, in vitro studies, and animal studies); *2*) studies that did not consider probiotics as the exclusive intervention or exposure factor; *3*) the composition of the infant gut microbiota was not measured except to determine species-specific abundance; and *4*) unavailable metadata and sequencing data.

### Study selection

Articles identified according to the search strategy were summarized using EndNote software 9. After removing duplicate articles, a final repository (containing 545 potential studies) was generated for subsequent screening. Two reviewers (HPD, YLL) independently screened the titles and abstracts of the identified studies to confirm whether they meet the eligibility criteria. Full texts were retrieved after screening, and then reviewed. Disagreements were resolved by discussion with a senior reviewer (TFW) to reach a consensus.

### Data sources and study population

We identified 16 studies that cataloged the gut microbiome of preterm infants exposed to probiotics using metagenomics. Eight of these studies did not make the raw data publicly available. We were unsuccessful in obtaining the raw reads from the authors because either the data were protected by ethical restrictions or the authors did not respond to our requests. In other cases, the raw reads were available, but some of these studies were unable to link the data to case–control status because this information was not reported in the metadata. Again, we were unsuccessful in obtaining metadata from the authors for 3 studies because either the data were protected by ethical restrictions or the authors did not answer our requests. Microbial amplicons metagenomics data for the 5 studies ultimately included in this study were downloaded directly from the European Nucleotide Archive or the NCBI Sequence Read Archive by listed accessions Table 1 [20,21,[26], [27], [28]]. The reanalysis of these public data sets followed the related ethical regulations. The 5 included studies were conducted in Sweden, New Zealand, Canada, the United Kingdom, and Austria. The overall meta-analysis sample size for preterm infants was 1816, with a sample size of 913 for intervention studies (control, *n* = 265; probiotics, *n* = 648) and 903 for observational studies (no exposure to probiotics, *n* = 420; exposure to probiotics, *n* = 483). The subjects of 4 studies were supplemented with multiple-strain probiotics (*n* = 637) [21,[26], [27], [28]], and those of 3 studies were supplemented with single-strain probiotics (*n* = 494) [20,26,27]. In summary, 1816 stool samples from preterm infants (2 y or younger) across 5 countries were used in our analyses. Three studies with control groups that provided available information on the type of probiotic supplementation in preterm infants were included in a subgroup analysis of α-diversity by probiotic type, with a sample size of 1437 (single-strain probiotics, *n* = 384; multiple-strain probiotics, *n* = 368) and subsequent analysis of gut microbial composition and functions [20,21,27].

**TABLE 1:** Characteristics of the included studies.^1^

Data origin, study population	Study design, sample size	16S rRNA region; sequencing platform	Probiotic species; dose (CFU/d); vehicle	Clinical registry number	Accession number
Sweden [20]	Longitudinal stool sample collection of 132 ELBW preterm infants (BW < 1000 g and GA between 23^+0^ and 27^+6^ wk) at 1, 2, 3, and 4 wk, PMW36, and 2 y. *N* = 558 (534)	V3-V4; Illumina MiSeq	*Limosilactobacillus reuteri* DSM 17938 (1.25 × 10^8^); oil drops	NCT01603368	PRJEB36531^2^
United Kingdom [21]	Longitudinal stool sample collection of 234 preterm infants (BW < 2000 g and GA ≤ 34 wk) at 0–9, 10–29, 30–49, and 50–99 d. *N* = 598 (581)	V1-V2; Illumina MiSeq	Infloran® probiotics (Desma Healthcare); *Bifidobacterium bifidum* (1 × 10^9^) and *Lactobacillus acidophilus* (1 × 10^9^); capsule	NA	PRJEB31653^2^
Austria [27]	Stool samples were taken from 54 preterm infants (BW < 1500 g and GA ≤ 34 wk) every other day throughout the first 2 wk of life, starting with meconium. *N* = 358 (322)	V4; Illumina MiSeq	*Lacticaseibacillus rhamnosus* (1 × 10^9^) split into 2 doses per day; NA *Bifidobacterium infantis* (2 × 10^9^) and *L. acidophilus* (2 × 10^9^); NA	DRKS00009290	PRJEB37883^2^
New Zealand [26]	Stool samples were collected from 227 MLPT infants (GA between 32^+0^ and 35^+6^ wk) who enrolled in the Different Approaches to Moderate and late preterm Nutrition (DIAMOND) trial at day 10 (chronological age) and 4 mo (corrected age). *N* = 320 (313)	V3-V4; Illumina MiSeq	LGG (Dicoflor60 Dicofarm SpA); NA; NA Labinic™ Drops (Biofloratech); *L. acidophilus* (6.7 × 10^8^), *B. bifidum* (6.7 × 10^8^), and *B. longum* subspecies *infantis* (6.7 × 10^8^); oil drops	ACTRN12616001199404	PRJNA645223^3^
Canada [28]	Longitudinal weekly stool sample collection from 59 preterm infants (GA < 31 wk) during the first month of life, starting with meconium. *N* = 131 (66)	V6; Illumina HiSeq 2500	FloraBABY probiotics (Renew Life); *Bifidobacterium breve* (6 × 10^8^), *B. bifidum* (4 × 10^8^), *B. infantis* (3 × 10^8^), *B. longum* (2 × 10^8^), and *L. rhamnosus* (5 × 10^8^); powder	ISRCTN66482337	PRJNA531325^3^

The sample size in parentheses is the number of samples eventually included in the meta-analysis after filtering low-quality or irrelevant samples.

Abbreviations: BW, birth weight; ELBW, extremely low birth weight; GA, gestational age; LGG, *Lacticaseibacillus rhamnosus* GG; MLPT, moderate-late preterm; PMW, postmenstrual week.

1 NA indicates that the information was not specified in the original article.

2 European Nucleotide Archive (ENA).

3 NCBI Sequence Read Archive (SRA).

### Data processing

The sequence data from the included studies were reprocessed [20,21,[26], [27], [28]]. To guarantee the consistency of the data necessary for meta-analyses in this study, sequence processing *QIIME2* (https://qiime2.org/) was used as the analysis pipeline and *SILVA* (release 132) was used as a 16S rRNA sequence reference [29]. α-Rarefaction curves of the bacterial communities were calculated using *QIIME2* with the default parameters. Samples with <10,000 sequencing reads were excluded from the subsequent analysis, resulting in the removal of 84 samples from the 4 studies [20,21,26,27]. To perform the analysis on a per-study basis, the samples were rarefied to the lowest depth in the study using Vegan rrarefy function, and the diversity, specnumber, and estimateR functions of Vegan (version 2.6-2) were applied to calculate the estimates of Shannon, Simpson (log base e), Chao1, and richness based on the rarefied amplicon sequence variant (ASV), respectively [30]. Bray–Curtis dissimilarities were generated based on the rarefied ASV from 5 combined studies using the vegdist function of the Vegan package (version 2.6-2) [30]. The relative taxonomic abundance was calculated based on the rarefied ASV. Dirichlet multinomial mixtures (DMM) were used to model the relationships between microbial communities [31], which were determined by 16S sequencing using the modified R code of Stewart et al. [32]. DMM clusters (community types) were determined based on the lowest Laplace approximation score. The proportion of samples occupying DMM clusters at each time point was plotted to visualize changes in preterm community types over time. The metagenomic functions of the microbial communities in preterm infants were predicted based on rarefied ASV tables using PICRUSt2 on the Wekemo Bioincloud (https://www.bioincloud.tech) web platform [33], and the relative abundances of the resulting MetaCyc functional pathways were calculated [34].

### Statistical analysis

The relationship between probiotics and gut microbial α-diversity in 3 independent cohorts was analyzed using locally estimated scatterplot smoothing with data fitting (Shannon, Simpson, richness, and Chao1 indexes) and visualized using the ggplot2 R package (version 3.4.4) [35]. The meta-analysis was executed using the meta R package (version 6.0-0) in R software (version 4.2.2) [36]. In the meta-analysis of α-diversity (Shannon, Simpson, richness, and Chao1 indexes), the effect size was assessed based on the standard mean difference (SMD), and the SMD of α-diversity between probiotics and controls was calculated based on the mean and SD. Heterogeneity was evaluated using *I*^2^ and Cochran Q statistics. According to the Cochrane recommendations, heterogeneity was considered moderate to substantial if *I*^2^ was between 30% and 75% and considerable if *I*^2^ was >75% [37]. The calculation of common and random-effect estimates for meta-analyses with continuous outcome data was performed in R software (version 4.2.2) using the meta R package (version 6.0-0) [36]. Consistent with previous studies [38], when there was significant heterogeneity (*P* ≤ 0.05), we used the random effects, and when there was no significant heterogeneity (*P* > 0.05), we used the fixed effects. For these α-diversity indexes, we performed a subgroup analysis using a Q test to analyze the SMD of studies assessing α-diversity across different probiotic types (single-strain or multiple-strain probiotics); on the contrary, sensitivity analysis was conducted to evaluate the robustness of the results. Funnel plots were used to evaluate publication bias. Considering the potential publication bias, the trim-and-fill method was applied to observe changes in pooled estimates after the imputation of data from potentially unpublished articles [39]. Principal coordinates analysis was conducted using the PCoA function of APE [40], and Adonis was calculated using the Adonis function of Vegan (version 2.6-2) with 999 permutations to assess the contribution of covariates to the variance in Bray–Curtis dissimilarity [30]. The relationship between probiotics and gut microbial Bray–Curtis dissimilarity in all samples (*n* = 1816) was analyzed using locally estimated scatterplot smoothing with data fitting (PCo1 and PCo2) and visualized using the ggplot2 R package (version 3.4.4) [35]. We applied the scree plot to explore the percentage of variation explained by each principal component (PC) and then carried out the UMAP dimensionality reduction analysis on the top several PCs identified by the scree plot, which was performed using the umap R package (version 0.2.10.0). Differential abundances of taxonomy (phylum, family, and genus) and metabolic pathways detected in ≥5% of the samples in the 3 independent cohorts were corrected for false discovery rate (FDR) using the Kruskal–Wallis test, and the differential taxonomy (*n* = 44) and metabolic pathways (top 50 of 347) common to the 3 independent cohorts were further visualized using the heatmap R package (version 1.0.12) [41]. Effect sizes for the meta-analysis of differential taxonomy and metabolic pathways were evaluated by SMD in the centered log-ratio (CLR) using the random-effect meta-analysis method (meta R package, version 6.0-0) [36]. Data were transformed using CLR (CLR function in easyCODA R package, version 0.34.3) [42], flowing imputing zeros were estimated using Bayesian-multiplicative replacements via the count zero multiplicative method (cmultRepl function in zCompostions R package, version 1.4.0-1) [43]. The dynamics of taxonomy and metabolic pathways in the preterm control and probiotic groups were analyzed using Area Plot tools in the Hiplot Pro (https://hiplot.com.cn/) web platform, based on the differential characteristics identified by meta-analysis. The dynamics of the differential taxa and metabolic pathways in the meta-analysis were visualized using the ggalluvial R package (version 0.12.5) [44].

## Results

### Study selection

**FIGURE 1:**
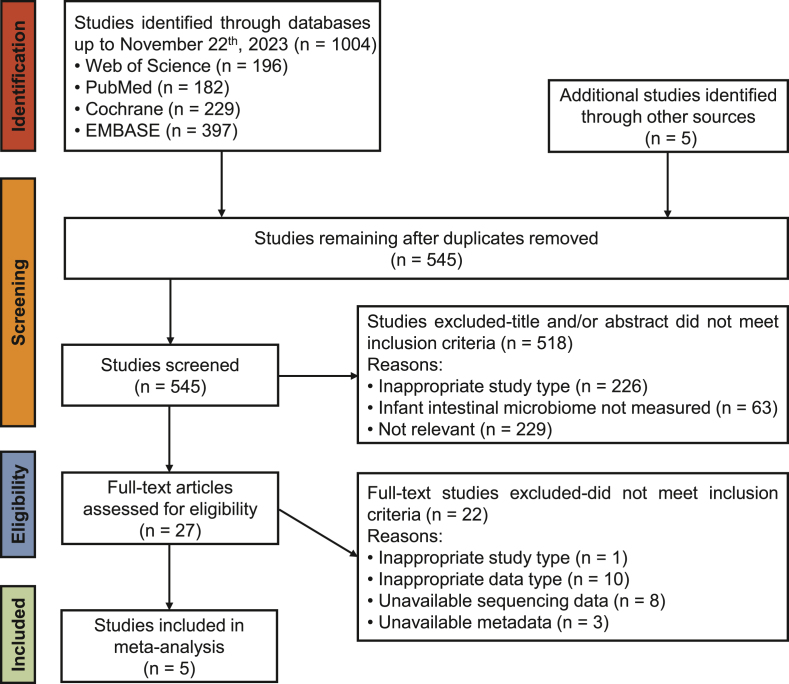
Preferred Reporting Items for Systematic Reviews and Meta-analyses (PRISMA) flow diagram.

The primary database search generated a list of 1009 potentially relevant studies. After removing duplicates, 545 studies remained for further screening. Furthermore, 518 studies were eliminated after the evaluation of titles and abstracts according to the study type and irrelevance; 27 studies qualified for full-text evaluation, and 22 were excluded owing to research type, data type, unavailable sequencing data, and unavailable metadata (Supplemental Table 2). Therefore, 5 publications were included in the meta-analysis (Figure 1).

### Study characteristics

The 5 included publications reported the results of observational studies [21,27] or randomized controlled trials [20,26,28], which were conducted between 2020 and 2021 in Sweden [20], New Zealand [26], Canada [28], United Kingdom [21], and Austria [27]. Only 1 of the 3 randomized controlled trials included was an unblinded study [26], and the remaining were double-blinded studies [20,28]. Four studies were multicenter trials [20,21,26,27], and 1 study was a single-center trial [28]. All studies recruited infants from neonatal intensive care units. The study population consisted of subjects with extremely low birth weight preterm infants, preterm infants, and moderate-late preterm infants, according to birth weight and/or duration of pregnancy, and the sample sizes were between 54 and 234. In the intervention studies, probiotic vehicles include powder [28] and oil drops [20,26]; probiotic species were 1 [20,26], 3 [26], or 5 [28]; and the minimum daily dose of probiotics was 1.25 × 10^8^ CFU [20], with a maximum dose of 2.01 × 10^9^ CFU [26]. However, in the observational studies, probiotic vehicles included capsules [21]; probiotic species were 1 [27] or 2 [21,27]; and the minimum daily dose of probiotics was 1 × 10^9^ CFU and the maximum dose was 4 × 10^9^ CFU [27]. The duration of the intervention in the randomized controlled trials ranged from 22 d to postmenstrual week (PMW) 36 [20,26,28], whereas the duration of exposure in the observational studies ranged from 68.5 d to PMW34 [21,27]. Details of each study are presented in Table 1. The timeline of sample collection for the included articles is shown in Supplemental Figure 1.

### Probiotic exposures and gut microbial α-diversity in preterm infants

**FIGURE 2:**
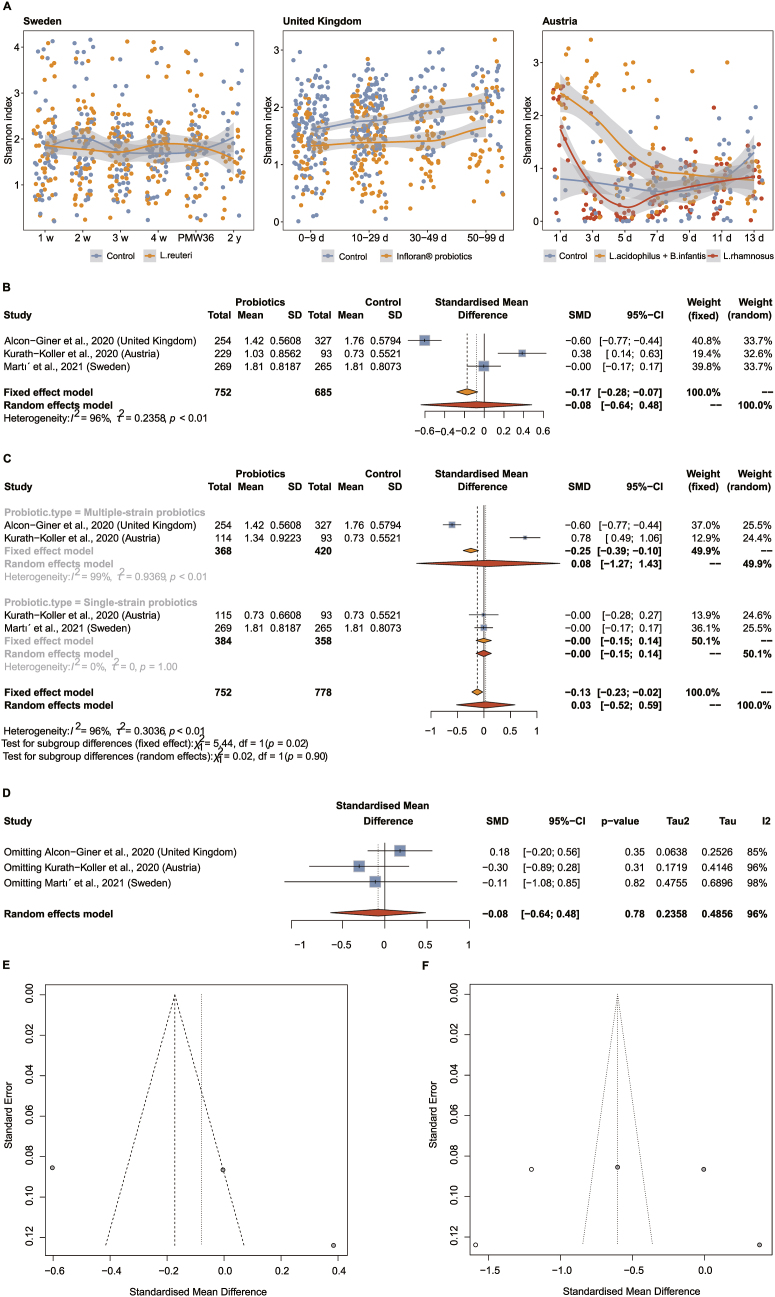
Probiotic exposures and gut microbial diversity in preterm infants. (A) LOESS fits (95% CIs shaded in gray) over time for Shannon index. (B) Forest plots of the association between probiotics and Shannon index. (C) Subgroup analysis of Shannon index between probiotics and control preterm infants by probiotic types. (D) Sensitivity analysis for multivariate analysis of Shannon index via excluding a study at a time and then pooling the remaining studies. (E) Funnel plot for assessing potential publication bias in the Shannon index before applying the trim-and-fill method. (F) Funnel plot for assessing potential publication bias in the Shannon index after applying the trim-and-fill method. *B. infantis*, *Bifidobacterium infantis*; *L. acidophilus*, *Lactobacillus acidophilus*; LOESS, locally estimated scatterplot smoothing; *L. reuteri*, *Limosilactobacillus reuteri*; *L. rhamnosus*, *Lacticaseibacillus rhamnosus*; PMW, postmenstrual week; SMD, standardized mean difference.

In preterm infants, across the 3 included studies [20,21,27], the association between probiotics and gut microbial α-diversity (Shannon, Simpson, Chao1, and richness indexes) was inconsistent compared with the control, and the α-diversity indexes varied dynamically over time (Figure 2A; Supplemental Figures 2A, **3**A, and **4**A). As shown in the forest plot, there were no significant changes in α-diversity in any of the preterm infants with or without probiotics: Shannon index—SMD: −0.08; 95% CI: −0.64, 0.48; *P* =  0.78 (Figure 2B); Simpson index—SMD: −0.12; 95% CI: −0.61, 0.36; *P* = 0.62 (Supplemental Figure 2B); richness index—SMD: −0.01; 95% CI: −0.81, 0.79; *P* = 0.98 (Supplemental Figure 3B); or Chao1 index—SMD: −0.00; 95% CI: −0.83, 0.83; *P* =  1.00 (Supplemental Figure 4B). Forest plots indicated significant heterogeneity (*I*^2^ > 75%; *P* < 0.01) or inconsistencies among the included studies. Therefore, we performed a subgroup analysis of the α-diversity indexes based on the type of probiotics. There were also no obvious differences in the subgroups of probiotic types between the probiotics and controls for the Shannon index (multiple-strain probiotics—SMD: 0.08; 95% CI: −1.27, 1.43; single-strain probiotics—SMD: −0.00; 95% CI: −0.15, 0.14; *P* = 0.90) (Figure 2C), Simpson index (multiple-strain probiotics—SMD: 0.02; 95% CI: −1.18, 1.23; single-strain probiotics—SMD: −0.04; 95% CI: −0.19, 0.10; *P* = 0.91) (Supplemental Figure 2C), richness index (multiple-strain probiotics—SMD: 0.22; 95% CI: −1.60, 2.04; single-strain probiotics—SMD: 0.23; 95% CI: −0.31, 0.76; *P* = 1.00) (Supplemental Figure 3C), and Chao1 index (multiple-strain probiotics—SMD: 0.24; 95% CI: −1.64, 2.11; single-strain probiotics—SMD: 0.25; 95% CI: −0.31, 0.81; *P* = 0.99) (Supplemental Figure 4C). Multiple-strain probiotics were a source of heterogeneity for the Shannon and Simpson indexes but not for the richness and Chao1 indexes.

The results of sensitivity analysis in gut microbial α-diversity found that adjusted SMDs ranged from −0.30 to 0.18 for the Shannon index (Figure 2D), from −0.30 to 0.10 for the Simpson index (Supplemental Figure 2D), from −0.37 to 0.34 for the richness index (Supplemental Figure 3D), and from −0.37 to 0.36 for the Chao1 index (Supplemental Figure 4D). The results of the sensitivity analyses excluding 1 study at a time and then pooling the remaining studies were not statistically significant (95% CI including 0) in agreement with the overall analyses, which may be due to substantial heterogeneity or inconsistency among the 3 independent cohorts of preterm infants, for example, probiotic species and doses. Publication bias was assessed using funnel plots, and the results suggested that potential publication bias was present in all gut microbial α-diversity indexes, as evidenced by the asymmetry of the funnel plots (Figure 2E; Supplemental Figures 2E, 3E, and 4E). Given the potential publication bias, the trim-and-fill method was performed by adding estimated SMDs of 2 potential unpublished articles to reach symmetry in the funnel plots (Figure 2F; Supplemental Figures 2F, 3F, and 4F). It is worth noting that the number of studies included in this meta-analysis was <10, so the funnel plot can be used only as a subjective qualitative method to judge publication bias, and more well-designed randomized controlled trials are needed in the future to elucidate the role of probiotics in reshaping the gut microbiome of preterm infants. The resulting pooled adjusted SMD for the Shannon index was −0.60 (95% CI: −1.32, 0.11; *P* = 0.10) (Supplemental Figure 5A), within the CIs of our main finding (SMD: −0.08, 95% CI: −0.64, 0.48; *P* = 0.78) (Figure 2B). The resulting pooled adjusted SMD for the Simpson index was −0.59 (95% CI: −1.21, 0.04; *P* = 0.07) (Supplemental Figure 5B), within the CIs of our main finding (SMD: −0.12, 95% CI: −0.61, 0.36; *P* = 0.62) (Supplemental Figure 2B). The resulting pooled adjusted SMD for the richness index was −0.70 (95% CI: −1.67, 0.27; *P* = 0.16) (Supplemental Figure 5C), within the CIs of our main finding (SMD: −0.01, 95% CI: −0.81, 0.79; *P* = 0.98) (Supplemental Figure 3B). The resulting pooled adjusted SMD for the Chao1 index was −0.72 (95% CI: −1.72, 0.29; *P* = 0.16) (Supplemental Figure 5D), within the CIs of our main finding (SMD: −0.00, 95% CI: −0.83, 0.83; *P* = 1.00) (Supplemental Figure 4B).

### Probiotic exposures and gut microbiota structure in preterm infants

**FIGURE 3:**
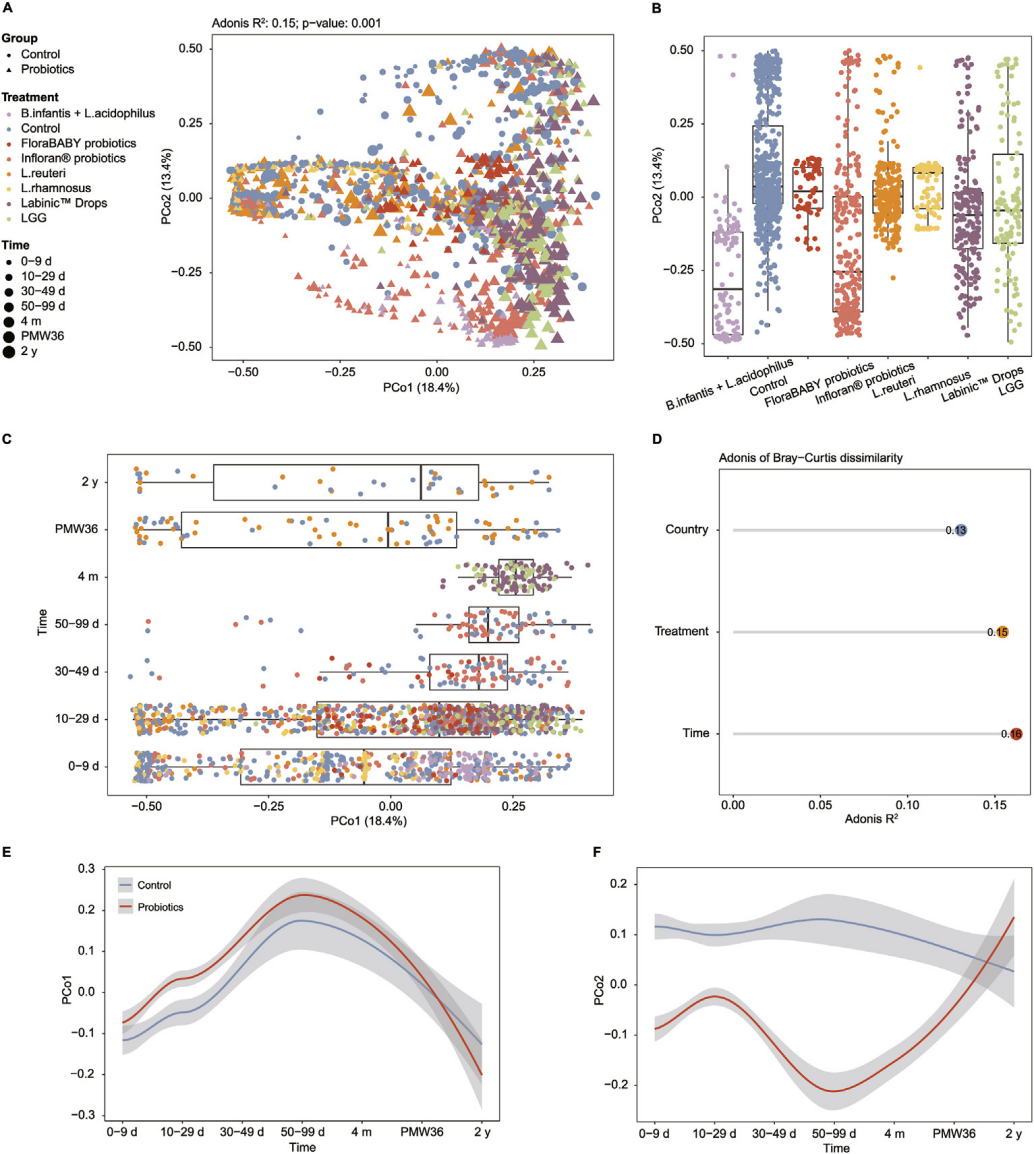
Probiotic exposures and gut microbiome structure in preterm infants. (A) PCoA of Bray–Curtis dissimilarity of 1816 fecal samples from preterm infants (2 y or younger) across 5 countries based on the rarefied ASV abundances. Point shape indicates group, point color indicates treatment, and point size is matching to the sampling times. Box plots demonstrate the distribution of treatments along PCo2 (B) and sampling times along PCo1 (C). (D) The lollipop plot shows the 3 host factors available that were significantly related to gut microbial variations (Bray–Curtis dissimilarity). Amount of variance (*R*^2^) and statistical significance were performed by PERMANOVA (modeled by “Adonis”). LOESS fits (95% CIs shaded in gray) over time for PCo1 (E) and PCo2 (F). ASV, amplicon sequence variant; *B. infantis*, *Bifidobacterium infantis*; *L. acidophilus*, *Lactobacillus acidophilus*; LOESS, locally estimated scatterplot smoothing; *L. reuteri*, *Limosilactobacillus reuteri*; *L. rhamnosus*, *Lacticaseibacillus rhamnosus*; LGG, *Lacticaseibacillus rhamnosus* GG; PCo, principal coordinate; PCoA, principal coordinates analysis; PMW, postmenstrual week.

The Bray–Curtis dissimilarity, generated from 1816 16S rRNA sequencing samples [20,21,[26], [27], [28]], indicated that sampling times and treatments were related to the first and second axes of variation, respectively (Figure 3A–C). A comparison of preterm infants supplemented with different probiotic species of the same type demonstrated that probiotics of the same species had a similar association on microbiota composition (Figure 3B). We used Adonis to assess the contribution of available clinical covariates to gut microbial variation derived from Bray–Curtis dissimilarity between samples. The treatment and host country were significant explanatory factors (*R*^2^ = 0.15 and 0.13; *P* = 0.001); however, sampling time had the strongest effect (*R*^2^ = 0.16; *P* = 0.001) (Figure 3D). The microbiomes of preterm infants supplemented with probiotics differed from those of infants not supplemented with probiotics within PMW36, although the differences between probiotic-treated and control infants were maximal at 50–99 d and then resolved by 36 wk (Figure 3E,F). The gut microbiota of preterm infants showed dynamic changes over time, suggesting an obvious sensitivity of the development of the preterm infant microbiota to the timing of probiotic supplementation (Figure 3F). Based on the fact that the first 2 PCs in Figure 3A captured only ∼30% of the variance in the data, we applied the scree plot to explore the percentage of variation explained by each PC (Supplemental Figure 6A) and found that the cumulative variation for the top 13 PCs was >90% (91.85%). We further performed the UMAP dimensionality reduction analysis on the top 13 PCs identified by the scree plot. As shown in the UMAP plot (Supplemental Figure 6B), we observed different effects of these PCs on the gut microbiome structure of preterm infants.

### Probiotic exposures and the gut microbial composition in preterm infants

Across the 3 included studies [20,21,27], Firmicutes and Proteobacteria were the dominant bacterial phyla during the first 2 y after birth in preterm infants, whereas Actinobacteria gradually became dominant when supplemented with Infloran® probiotics (*Bifidobacterium bifidum* + *Lactobacillus acidophilus*) or *Bifidobacterium infantis* + *L. acidophilus* (Supplemental Figure 7). Staphylococcaceae and Enterobacteriaceae were the most abundant bacterial families in preterm infants during the first 2 y, and the relative abundance of Bifidobacteriaceae tended to increase over time, but the level of presence remained low. However, Bifidobacteriaceae gradually became dominant when supplemented with Infloran® probiotics (*B. bifidum* + *L. acidophilus*) or *B. infantis* + *L. acidophilus* (Supplemental Figure 8). *Staphylococcus* and *Enterococcus* spp. were the dominant genera in preterm infants, and the relative abundances of *Staphylococcus* spp tended to decrease over time. The level of *Escherichia-Shigella* spp. was also high throughout the neonatal period, whereas *Bifidobacterium* and *Lactobacillus* spp. were low. The abundance of *Lactobacillus* spp. increased when supplemented with *Limosilactobacillus reuteri* or *Lacticaseibacillus rhamnosus*, and interestingly, *Bifidobacterium* spp. gradually became dominant when supplemented with Infloran® probiotics (*B. bifidum* + *L. acidophilus*) or *B. infantis* + *L. acidophilus* (Supplemental Figure 9). It is worth noting that the relative abundances of lactobacilli (phylum, family, and genus in Supplemental Figures 7–9, respectively) were not sustained over time even during probiotic administration (in contrast to bifidobacteria). Variations in the gut microbiota at the phylum, family, and genus levels in the 3 independent cohorts were analyzed using the Kruskal–Wallis test (differential taxa were not detected in 1 of the cohorts), identifying 44 common differential taxa (FDR < 0.05) included in the meta-analysis (Supplemental Table 3).

**FIGURE 4:**
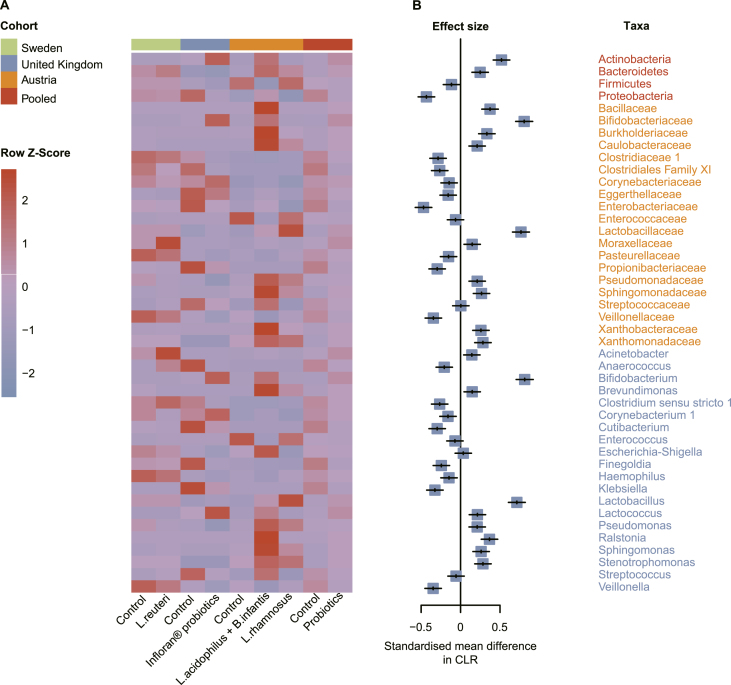
Probiotic exposures and the preterm infant gut microbiota. (A) Heatmap of all samples (*n* = 1437) showing the 44 taxa with significant changes related to probiotics use in each cohort and meta-analysis. (B) Effect sizes were calculated by SMD in CLR using a random-effect meta-analysis method. Differential abundances of phylum, family and genus detected in ≥5% of the samples in that study was corrected for FDR using the Kruskal–Wallis test. *B. infantis*, *Bifidobacterium infantis*; CLR, centered log-ratio; FDR, false discovery rate; *L. acidophilus*, *Lactobacillus acidophilus*; *L. reuteri*, *Limosilactobacillus reuteri*; *L. rhamnosus*, *Lacticaseibacillus rhamnosus*; SMD, standardized mean difference.

There was substantial heterogeneity in the differences in the relative abundance of the gut microbiota between the control and probiotic groups in the 3 independent cohorts of preterm infants. For example, the level of *Enterococcus* sp. increased in preterm infants supplemented with probiotics compared with that in the controls in the 2 studies conducted in Sweden and the United Kingdom, but the opposite was observed in the Austrian study. Notably, the enrichment of *Lactobacillus* spp. in probiotic compared with that in nonprobiotic preterm infants was observed in the 2 studies from the United Kingdom and Austrian, but the opposite was observed in the study from Sweden (Figure 4A). However, the included studies showed significant consistencies. At the family level, the levels of Moraxellaceae and Sphingomonadaceae increased significantly overall (consistent in all 3 studies) in preterm infants with probiotic compared with those with nonprobiotic supplementation, in contrast to Clostridiales Family XI and Veillonellaceae, which had a consistent downward trend. At the genus level, *Finegoldia* and *Veillonella* decreased in preterm infants supplemented with probiotics, whereas the abundance of *Acinetobacter* increased (consistent in all 3 studies) (pooled *P* < 0.05) (Supplemental Table 3 and Figure 4). Details of the meta-analysis are summarized in Supplemental Table 3, with significant changes observed in 39 of the 44 taxa (pooled *P* < 0.05). As shown in the forest plot in Figure 4B, 21 taxa, including *Bifidobacterium* and *Lactobacillus* spp. significantly increased in preterm infants supplemented with probiotics, whereas the opposite result was observed for another 18 taxa, including *Veillonella* and *Klebsiella* spp.

**FIGURE 5:**
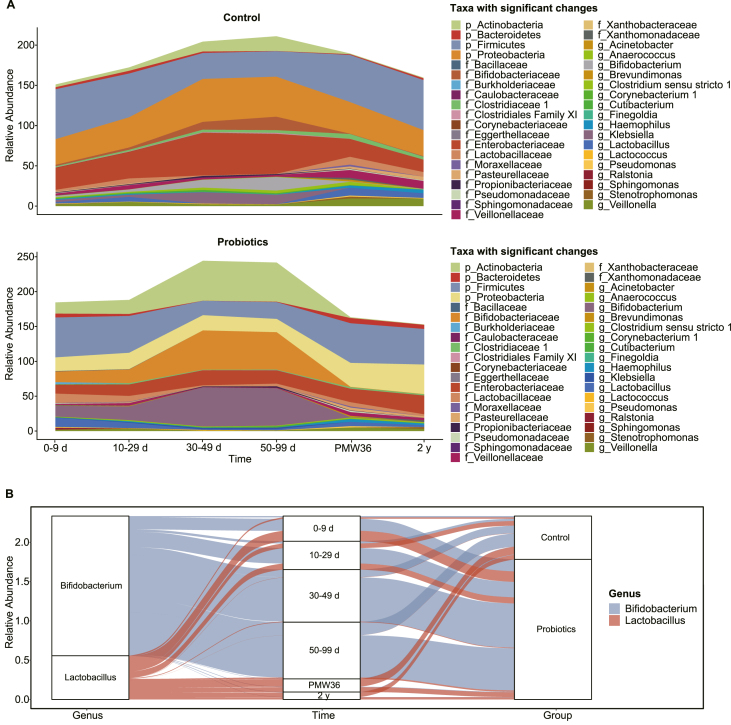
Dynamics of the relative abundances of significantly altered taxa in the control and probiotic groups in preterm infants. (A) Comparison of differences in taxonomic structure at the phylum, family, and genus levels between controls and preterm infants receiving probiotics. (B) Alluvial diagram showing the relative abundance of *Bifidobacterium* and *Lactobacillus* spp. in the control and probiotic groups at different time points. The left panel shows the bacterial genera, the middle panel the time points, and the right panel the groups. The curved lines across panels indicate the relative abundance of bacterial genera, while the colors correspond to different bacterial genera. PMW, postmenstrual week.

Longitudinal analysis of the significantly altered taxa between the control and probiotic groups in preterm infants revealed major shifts in the levels of several taxa during the first 2 y of life. The relative abundance of Lactobacillaceae and *Lactobacillus* sp. in preterm infants supplemented with probiotics was consistently higher than that in controls during probiotic supplementation, whereas the opposite result was observed after the cessation of probiotic supplementation (PMW36), whereas the dynamics of the relative abundance of *Klebsiella* sp. were the opposite of these 2 taxa. Similarly, the levels of Bacillaceae, Actinobacteria, Bifidobacteriaceae, and *Bifidobacterium* sp. were consistently higher in preterm infants receiving probiotics than those in controls until the 2 y of life, after which levels decreased, whereas Proteobacteria and Enterobacteriaceae were the opposite of the above taxa. Interestingly, probiotic administration resulted in a consistently lower relative abundance of Firmicutes, Clostridiaceae 1, Veillonellaceae, *Veillonella* sp., and *Finegoldia* sp. than those in the controls in preterm infants during the first 2 y after birth (Figure 5). Notably, *Bifidobacterium* sp., rather than *Lactobacillus* sp., gradually dominated during probiotic supplementation in preterm infants (especially at 30–99 d), but levels of *Bifidobacterium* sp. decreased sharply after cessation of probiotic supplementation (PMW36) (Figure 5B).

### Role of probiotics in shaping the gut community of preterm infants

**FIGURE 6:**
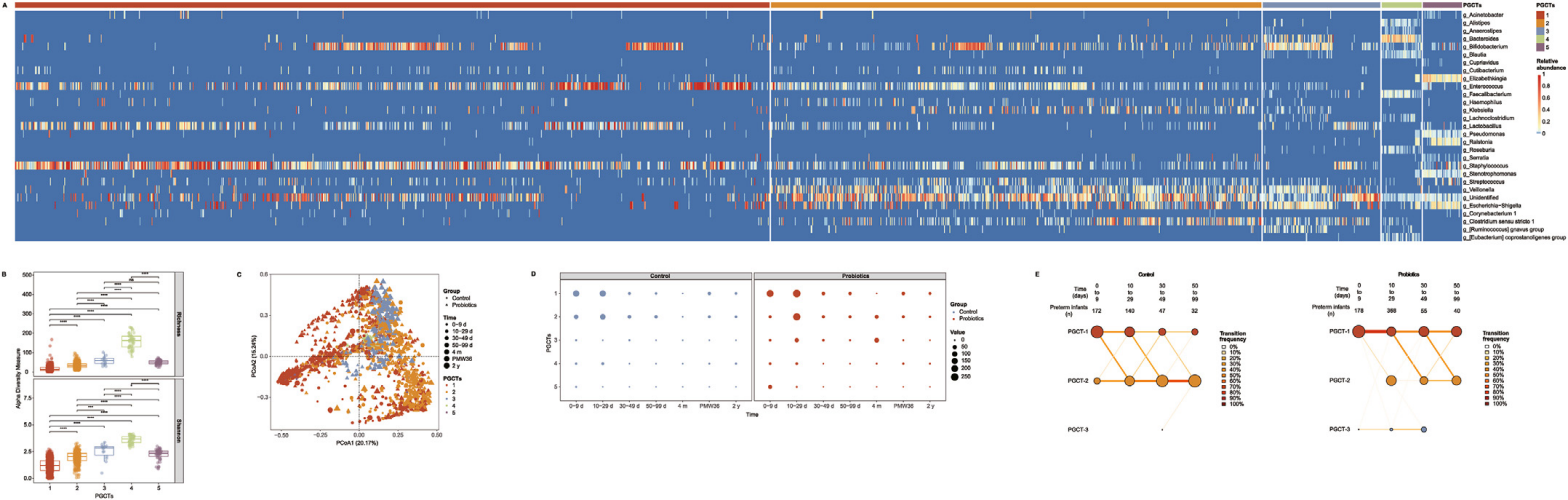
DMM clustering of 16S rRNA gene sequencing data. (A) Heatmap of all samples (*n* = 1816) showing the relative abundance of the 10 most dominant bacterial genera, stratified by PGCTs. (B) Box plots showing the α-diversity (richness and Shannon diversity) for each PGCT. The center line denotes the median, the box limits denote the IQR, and the whiskers extend to the limits. (C) PCoA of Bray–Curtis dissimilarity of 1816 fecal samples from preterm infants (2 y or younger) across 5 countries based on the rarefied ASV abundances. Point shape indicates group, point color indicates PGCTs, and point size is matching to the sampling times. (D) Distribution of samples in the identified 5 PGCTs (y axis), clustered using DMM at each time point (x axis) in the total samples (*n* = 1816). Sizes of circles are scaled by the occurrence number of each PGCT within at each time point. (E) Transition model showing the progression of samples (*n* = 1282; PRJEB36531 was not included in the analysis as the subject ID were not provided owing to data protections reasons) through each PGCT from day 0 to day 99, based on the group. The nodes and edges are sized based on the total counts. Nodes are colored according to PGCT and edges by the transition frequency. Transitions with <1% frequency are not shown. PCoA, principal coordinates analysis; PGCT, preterm gut community type; PMW, postmenstrual week.

DMM modeling was used for 16S rRNA gene sequencing to determine 5 clusters to be optimal (based on lowest Laplace approximation), herein termed preterm gut community types (PGCTs). The genera *Staphylococcus*, *Enterococcus*, *Bifidobacterium*, *Bacteroides*, and *Elizabethkingia* dominated the PGCTs (Figure 6A). The PGCTs exhibited significant differences in richness and Shannon diversity and microbial compositions (Figure 6B,C) and had different spatial and temporal distributions (Figure 6D). Specifically, PGCT-1 and PGCT-2 were common in the control sample for most of the first 2 y of life, with the exception of 4 mo. However, PGCT-1 and PGCT-5 were common in preterm samples supplemented with probiotics at 0–9 d; 3 PGCTs were common in preterm samples supplemented with probiotics at 10–99 d (PGCT1, 2, 3); PGCT-2 and PGCT-3 were common in preterm samples supplemented with probiotics at 4 mo; and common PGCTs in preterm samples supplemented with probiotics at PMW36 and 2 y were consistent with those of the control sample (PGCT-1 and PGCT-2). *Staphylococcus* and *Enterococcus* spp. dominated during the first 99 d of preterm infants, in which 63% of preterm infants transitioned from PGCT-1 to PGCT-2 (*Staphylococcus* and *Enterococcus* spp. were dominant in both PGCTs) (Supplemental Table 4). The community type during the initial developmental phase of preterm infants supplemented with probiotics was dominated by PGCT-1, with *Staphylococcus* and *Enterococcus* as the main genera. As preterm infants aged, the microbiomes of their stools diversified into PGCT-1, PGCT-2, and PGCT-3 during days 10–49 and, as expected, the bacterial richness and Shannon diversity increased significantly through each PGCT. The PGCT-3, which had the highest α-diversity, had higher abundance of *Bifidobacterium* and *Bacteroides* spp. Community types in probiotic-supplemented preterm infants at 50–99 d were dominated by PGCT-1 and PGCT-2 (*Staphylococcus* and *Enterococcus* spp. were dominant in both PGCTs) (Figure 6E).

### Probiotic exposures and the gut microbial functions in preterm infants

**FIGURE 7:**
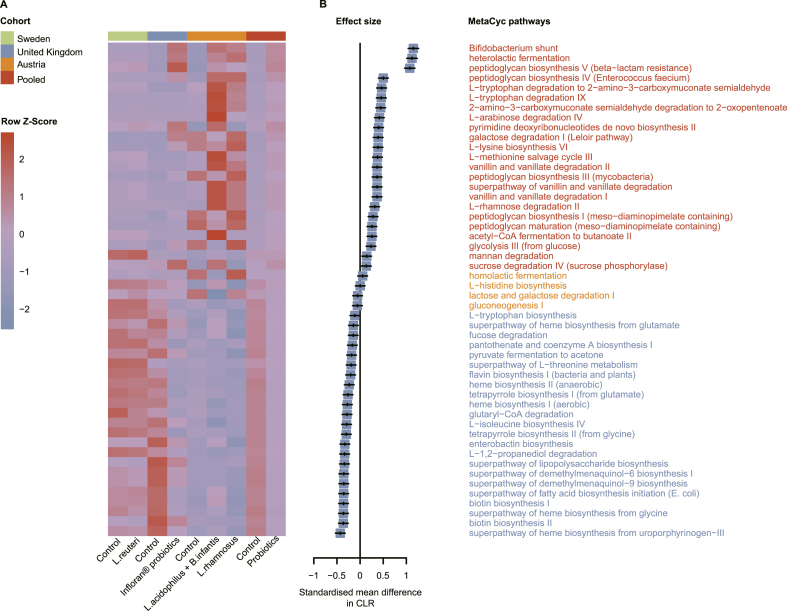
Probiotic exposures and gut bacterial metabolic pathway abundances in preterm infants. Only selected relevant pathways are displayed (a complete overview is demonstrated in Supplemental Figure 10). (A) Heatmap of all samples (*n* = 1437) showing the 50 metabolic pathways with significant changes related to probiotics use in each cohort and meta-analysis. (B) Effect sizes were calculated by SMD in CLR using a random-effect meta-analysis method. Differential abundances of metabolic pathways detected in ≥5% of the samples in that study was corrected for FDR using the Kruskal–Wallis test. *B. infantis*, *Bifidobacterium infantis*; CLR, centered log-ratio; FDR, false discovery rate; *L. acidophilus*, *Lactobacillus acidophilus*; *L. reuteri*, *Limosilactobacillus reuteri*; *L. rhamnosus*, *Lacticaseibacillus rhamnosus*.

Tests for the differential abundance of predicted pathways between the control and probiotic groups of preterm infants were performed, as with the taxonomic data. Variations in gut bacterial metabolic pathways in the 3 independent cohorts were analyzed using the Kruskal–Wallis test, identifying 347 common differential metabolic pathways (FDR < 0.05) included in the meta-analysis (Supplemental Table 5). Across the 3 included studies [20,21,27], important concordance was found despite significant differences in the functional pathways of the gut microbial MetaCyc functional pathways between the control and probiotic groups of preterm infants. Specifically, most of the predicted pathways enriched in the probiotic group were associated with peptidoglycan and pyrimidine deoxyribonucleotides biosynthesis, vanillin and vanillate, mannan, glycine betaine and protocatechuate degradation, and L-methionine salvage cycle III (consistent in all 3 studies), whereas controls were enriched in pathways related to heme, tetrapyrrole, biotin and flavin biosynthesis, glutaryl-CoA, and L-1,2-propanediol degradation, the metabolism of L-threonine, and the production of acetone via pyruvate fermentation (consistent in all 3 studies) (pooled *P* < 0.05) (Figure 7; Supplemental Table 5 and Supplemental Figure 10). Details of the meta-analysis are provided in Supplemental Table 5, showing significant changes in 273 of the 347 predicted metabolic pathways (pooled *P* < 0.05). As shown in the forest plot in Figure 7B and Supplemental Figure 10B, 139 metabolic pathways, including peptidoglycan biosynthesis, vanillin, and vanillate degradation, were significantly increased in preterm infants supplemented with probiotics, whereas the opposite result was observed for another 134 metabolic pathways, including heme and tetrapyrrole biosynthesis.

**FIGURE 8:**
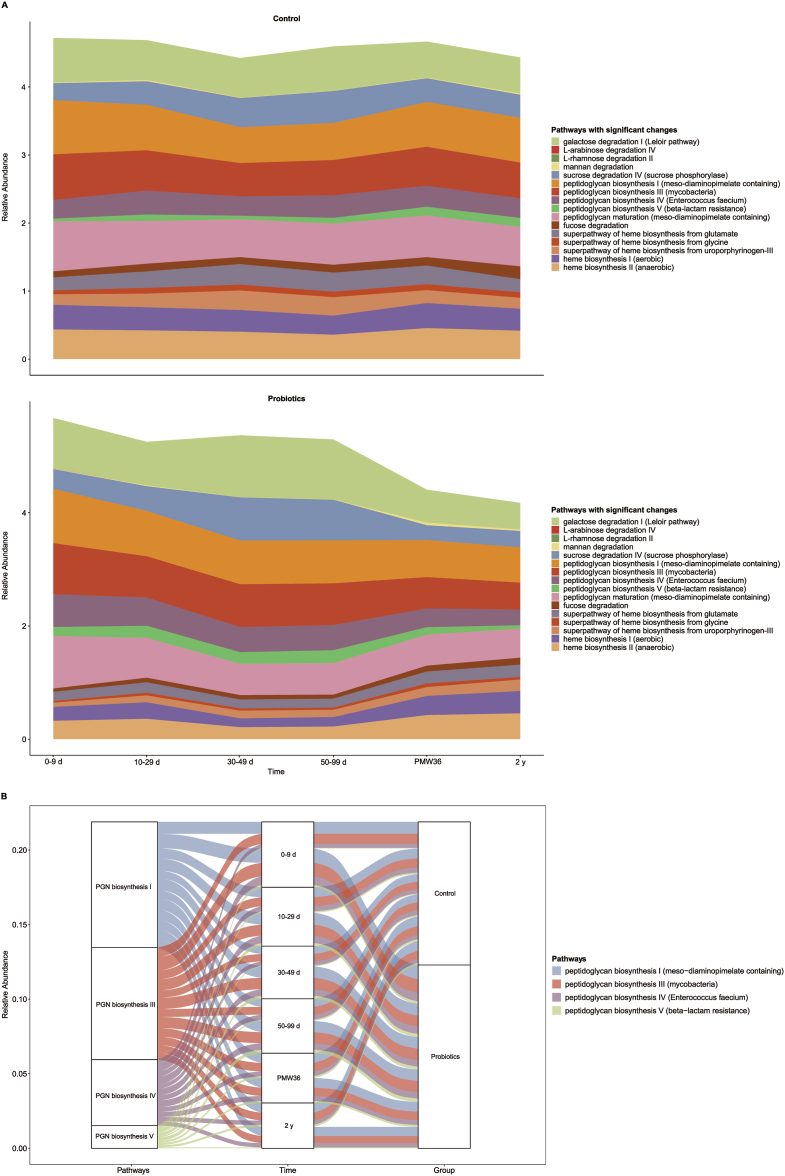
Dynamics of the relative abundances of significantly altered metabolic pathways in the control and probiotic groups in preterm infants. (A) Comparison of the structure of differential metabolic pathways between controls and preterm infants receiving probiotics. (B) Alluvial diagram showing the relative abundance of several pathways associated with peptidoglycan biosynthesis in the control and probiotic groups at different time points. The left panel shows the metabolic pathways, the middle panel the time points, and the right panel the groups. The curved lines across panels indicate the relative abundance of metabolic pathways, while the colors correspond to different metabolic pathways. PGN, peptidoglycan; PMW, postmenstrual week.

Longitudinal analysis of the significantly altered metabolic pathways between the control and probiotic groups in preterm infants revealed major shifts in the levels of several pathways during the first 2 y of life. The relative abundance of metabolic pathways involved in peptidoglycan biosynthesis (I and III), and sucrose degradation was consistently higher in probiotic-supplemented preterm infants than that in the controls during probiotic supplementation, whereas the opposite result was observed after the cessation of probiotic supplementation (PMW36). Similarly, the relative abundance of metabolic pathways associated with peptidoglycan biosynthesis (IV and V) and galactose degradation was consistently higher in preterm infants supplemented with probiotics than those in the controls until the age of 2 y, with the opposite result occurring. In contrast, changes in the levels of several pathways involved in heme biosynthesis were diametrically opposite to those of the aforementioned metabolic pathways. Interestingly, probiotic administration resulted in a consistently lower relative abundance of fucose degradation and the superpathway of heme biosynthesis from glycine than that in controls during the first 2 y of life in preterm infants (Figure 8). Notably, the relative abundance of several pathways associated with peptidoglycan biosynthesis was consistently higher in preterm infants supplemented with probiotics than that in the controls during the entire period of probiotic supplementation (0–99 d), but this effect disappeared after cessation of probiotic supplementation (PMW36), and of the 4 relevant metabolic pathways, peptidoglycan biosynthesis I and III accounted for a larger proportion (Figure 8B).

## Discussion

Previous studies have indicated that microbial diversity in preterm infants supplemented daily with probiotics increased for a specific period [20,21,27] and then reached similar [20,27] or lower diversity than that of controls [21]. A meta-analysis of 3 independent cohorts of preterm infants indicated substantial heterogeneity or inconsistency in the differences in α-diversity indexes between the control and probiotic groups. Therefore, we performed a subgroup analysis of the α-diversity indexes by probiotic type (single-strain or multiple-strain probiotics). The results showed that multiple-strain probiotics were a source of heterogeneity for the Shannon and Simpson indexes. Our combined results (random-effect model) showed no significant differences in the α-diversity indexes in all preterm infants with or without probiotics, after excluding sampling times. The results of the sensitivity meta-analysis excluding any of the 3 above-described studies for microbial α-diversity were consistent with the overall analysis [20,21,27], none of which was statistically significant. In subgroup analysis based on the type of probiotic used, the results remained the same. We speculate that probiotics have strain-specific effects on gut microbial diversity in preterm infants.

Consistent with previous studies [20,21,27], we showed that the gut microbiota of preterm infants supplemented with probiotics was significantly different from that of controls. The observed differences were robust in different sampling cohorts, despite a limited fraction of the variation in microbial amplicon metagenomic data being explained by probiotics, suggesting that alterations in the gut microbiota of preterm infants receiving probiotics are a general phenomenon. Analysis of Bray–Curtis dissimilarity across all 5 independent cohorts of preterm infants revealed that sampling time and treatment were forcefully related to the first and second axes of variation [20,21,[26], [27], [28]], respectively, and that probiotics of the same species had more similar effects on microbiota composition. Notably, among the available host factors, although treatment and host country were significant explanatory factors for gut microbial variation, sampling time played the strongest role, suggesting that multiple factors are involved in the different microbiome profiles over the first 2 y of life, rather than the probiotics alone. The microbiome profile of preterm infants supplemented with probiotics showed dynamic changes over time, suggesting the obvious sensitivity of microbiota development in preterm infants to the timing of probiotic supplementation and that probiotics are important drivers of gut microbiome development in preterm infants. Concerns have been raised regarding the use of D-lactate–producing probiotics in infants [45]. To date, there have been no cases of D-lactic acidosis due to intake of D-lactate–producing *Lactobacillus* sp. in either healthy infants or preterm infants. However, cases of D-lactic acidosis have been reported in infants and adults with short bowel syndrome or small intestinal bypass [[46], [47], [48], [49]], as well as in children with small bowel resection [50]. To our knowledge, deficiencies of thiamine, riboflavin, and biotin have been associated with lactic acidosis in preterm infants [[51], [52], [53]]. In addition, feeding the acidified milk can also lead to acidosis in preterm infants, largely attributed to the fact that many of the premature infants were not capable of handling this relatively small acid load [54].

Our results are consistent with those of previous studies showing that in preterm infants [20,21,27], Firmicutes and Proteobacteria were the dominant bacterial phyla; Staphylococcaceae and Enterobacteriaceae were the most abundant bacterial families; *Staphylococcus*, *Enterococcus*, and *Escherichia-Shigella* were the dominant genera; and there was a tendency for the relative abundance of *Staphylococcus* spp to decrease over time, whereas *Bifidobacterium* and *Lactobacillus* spp. were present at lower. In contrast, the microbial signatures after probiotic supplementation were dominated by the administered probiotic genera, which reflected in the higher relative abundance in samples from preterm infants supplemented with probiotics. Despite expected variations, our meta-analysis revealed some cross-study consistency. In all 3 studies [20,21,27], the levels of Moraxellaceae, Sphingomonadaceae, and *Acinetobacter* consistently increased, whereas those of Clostridiales Family XI and Veillonellaceae consistently decreased in probiotic compared with nonprobiotic preterm infants. Specifically, the relative abundances of *Finegoldia* and *Veillonella* spp persistently decreased in probiotic compared with those of nonprobiotic preterm infants. The relative abundance of *Acinetobacter* sp. has been demonstrated to be positively related to a lower allergy prevalence [55], and a higher abundance of *Acinetobacter* sp. has been found in children without respiratory tract infections than that in those with respiratory tract infections [56]. However, the increased abundance of *Finegoldia* sp. contributes to increased susceptibility to early-life eczema [57], recurrence of Crohn disease [58], and later risk of HIV seroconversion [59]. In addition, the expansion of the potential pathogenic bacterium *Veillonella* sp. was positively correlated with multiple pathologies, including inflammatory bowel disease [60], colorectal cancer [61], childhood asthma [62], and autism spectrum disorder [63].

Longitudinal analysis of significantly altered taxa between the control and probiotic groups in preterm infants revealed that the abundance of *Finegoldia* and *Veillonella* spp. was significantly lower in preterm infants supplemented with probiotics at 0–9 and 10–29 d from birth. During probiotic supplementation, preterm infants supplemented with probiotics had remarkably higher abundances of Actinobacteria, Bifidobacteriaceae, Lactobacillaceae, *Bifidobacterium*, and *Lactobacillus*, whereas the controls had observably higher abundances of Proteobacteria, Enterobacteriaceae, and *Klebsiella*. Interestingly, the levels of *Bifidobacterium* sp. were higher than those of *Lactobacillus* sp. in the gut microbiota of preterm infants supplemented with a mix of probiotics, although both probiotic strains were administered at the same dosage. *Bifidobacterium* sp. has been reported to contain specific genes that contribute to human milk oligosaccharide utilization, which may be associated with the higher abundance of *Bifidobacterium* sp. in breast milk–fed preterm infants supplemented with mixed probiotics [21,64,65]. Notably, the levels of *Bifidobacterium* sp. sharply decreased after the cessation of probiotic supplementation, which might indicate a transient and limited persistence of *Bifidobacterium* sp. in the preterm gut microbiota. However, preterm infants differ from term breastfed infants in that term infants colonization with a probiotic strain of *Bifidobacterium longum* subspecies *infantis* (*B. infantis*) was maintained for months after cessation of probiotic administration [66,67], likely due to selective consumption of human milk oligosaccharides [68].

The preterm microbiota displays “delayed” maturity with prolonged membership of facultative anaerobic bacteria compared with that of the predominantly strict anaerobic community of term infants [10,69,70]. Most infants were initially dominated by *Staphylococcus* sp., then transitioned to a state dominated by *Klebsiella*, *Enterococcus*, or *Escherichia* spp. as infants aged [7,8,10,[71], [72], [73]]. Consistent with another cohort, we observed that preterm infant gut bacterial communities were predominantly clustered into 5 PGCTs, which was supported by the metagenomic sequencing data [16]. Transition model revealed that preterm infants were more likely to transition into the *Staphylococcus* sp.–dominated and *Enterococcus* sp.–dominated PGCT-2. As preterm infants supplemented with probiotics age, the microbiomes of their stools gradually differentiated from the initial PGCT-1 to PGCT-2 and PGCT-3 during days 10–49, and the bacterial richness and Shannon diversity of these PGCTs increased significantly. The main genera of PGCT-1 and PGCT-2 were *Staphylococcus* and *Enterococcus*. The PGCT-3 had the highest α-diversity, with higher abundance of *Bifidobacterium* and *Bacteroides* spp, implying a more mature pattern closer to that of the full-term healthy neonate [74].

Across the 3 included studies [20,21,27], important concordances were found, although the relative abundances in gut bacterial metabolic pathways in the control and probiotic groups of preterm infants were largely heterogeneous. The major microbial pathways involved in peptidoglycan biosynthesis and vanillin and vanillate degradation consistently increased, whereas the relative abundances of crucial metabolic pathways associated with heme biosynthesis consistently decreased in probiotic-supplemented compared with those nonprobiotic-supplemented preterm infants. Longitudinal analysis of the significantly altered metabolic pathways between the control and probiotic groups of preterm infants revealed that the relative abundances of metabolic pathways involved in peptidoglycan biosynthesis (I, III, IV, and V), sucrose degradation, and galactose degradation were significantly higher in preterm infants supplemented with probiotics during the probiotic period, whereas the control group had a significantly higher abundance of several pathways involved in heme biosynthesis. Bacterial cell wall peptidoglycan is a crucial gut microbial signal capable of influencing neurodevelopment and behavior [75], physiologic development [76], inflammatory responses [77], immune responses [78], and enhancing host tolerance to enteric pathogens [79], suggesting that peptidoglycan can act as a driver of health and disease, mediating the host–gut microbiota crosstalk. Notably, several pathways involved in peptidoglycan biosynthesis were consistently upregulated in preterm infants supplemented with probiotics than those in controls during probiotic supplementation, indicating a novel mechanism by which probiotics regulate immunity in preterm infants. However, increased levels of heme biosynthesis affect the effectiveness of fecal microbiota transplantation in ulcerative colitis patients [80]. These findings may offer insights into the potential mechanisms underlying the lower risk of subsequent disease in preterm infants supplemented with probiotics in early life.

Despite our efforts, this meta-analysis had some limitations. First, the sample collection, DNA extraction, sequencing of targeted regions, and platforms varied among the included studies, resulting in technical variation. We mitigated the batch effect as much as possible using a consistent workflow and statistical methods. Second, the gestational age and birth weight of preterm infants varied across the included studies, and only 1 study was conducted on extremely preterm infants. Therefore, we were unable to analyze the associations of probiotics in this vulnerable subgroup of infants. Third, the probiotics used and their doses varied among the 5 included studies; thus, further subgroup analyses of probiotic species/strains and doses could not be performed. Finally, because of the limited availability of relevant metadata, we were unable to consider more detailed confounding factors such as gestational age, birth mode, sex, and feeding practice. Although promising, given the heterogeneity in pooled analyses, additional well-designed randomized controlled trials are needed to elucidate the role of probiotics in reshaping the gut microbiome of preterm infants and to identify taxa that may be mechanistically related to reducing risk of NEC, LOS, and asthma in preterm infants.

In summary, the findings of our meta-analysis found consistency among the 5 countries, which might contribute to illustrating the association between probiotics and gut microbiota in preterm infants. Although probiotics have strain-specific effects on gut microbial α-diversity in preterm infants, the associations of the same probiotic species on microbiota composition are similar. During the first 2 y after birth, time was the primary factor influencing the preterm gut microbiome, followed by probiotics and country. Our meta-analysis revealed consistent differences in gut microbial composition and predicted functional pathways between preterm infants receiving probiotics and controls. The enrichment of *Acinetobacter*, *Bifidobacterium*, and *Lactobacillus* spp and the depletion of the potentially pathogenic bacteria such as *Finegoldia*, *Veillonella*, and *Klebsiella* spp. were the most consistent changes in the gut microbiota of preterm infants supplemented with probiotics. In addition, preterm infants who did not receive probiotics were more likely to transition into the *Staphylococcus* and *Enterococcus*-dominated PGCT-2, whereas probiotics induced a shift in the microbiota to multiple PGCTs and the highest α-diversity was found in PGCT-3, which was enriched in *Bifidobacterium* and *Bacteroides* spp. At the functional level, the major predicted microbial pathways involved in peptidoglycan biosynthesis consistently increased in preterm infants supplemented with probiotics; in contrast, the crucial pathways associated with heme biosynthesis consistently decreased. In conclusion, our findings support the idea that probiotics contribute to reshaping the developmental trajectory of homeostatic gut microbiota in preterm infants at both the taxonomic and functional levels of the bacterial community.

## Author contributions

The authors’ responsibilities were as follows – PH, WC, HZ, QZ: designed research; PH, LY, FT: conducted research; PH, HZ, QZ: analyzed data; PH, QZ: wrote the article; HZ, QZ: had primary responsibility for final content; and all authors: have read and approved the final manuscript.

## Conflict of interest

The authors report no conflicts of interest.

## Funding

This work was supported by the National Natural Science Foundation of China (No. U23A20259); the Special Fund for Science and Technology Program of Jiangsu Province (BM2022019); and the Fundamental Research Funds for the Central Universities (JUSRP622013).

## Data availability

All raw 16s rRNA sequencing data used in the analyses of this study have been previously published [20,21,[26], [27], [28]], which are available from the European Nucleotide Archive (ENA) or the NCBI Sequence Read Archive (see Table 1 for the identifiers of included data sets).
