# Supplementary material for: Effects of Probiotics on Preterm Infant Gut Microbiota Across Populations: A Systematic Review and Meta-Analysis
Source: Adv Nutr. 2024 May 20;15(6):100233. doi: 10.1016/j.advnut.2024.100233 (PMC11251410; doi:10.1016/j.advnut.2024.100233)
Supplement: Multimedia component 1 [file mmc1.docx]

**Effects of probiotics on preterm infant gut microbiota across populations: A systematic review and meta-analysis**

**Pandi He**

**Supplementary Materials**

**Supplemental Table 1.** Search strategy.

**Supplemental Table 2.** Full-text studies excluded.

**Supplemental Table 3.** Significant taxa obtained by Kruskal-Wallis test in pooled data, and meta-analysis of the differential taxa.

**Supplemental Table 4.** The result of DMM transitions over time.

**Supplemental Table 5.** Significant metabolic pathways obtained by Kruskal-Wallis test in pooled data, and meta-analysis of the differential pathways.

**Supplemental Figure 1.** Timeline of sample collection among the included studies exploring the association between probiotics and gut microbiota in preterm infants. The filled diamonds (◊) represent the start and end of probiotics supplementation for preterm infants. The filled circles (○) represent the near times when the samples were collected (timeline not to scale). Studies involving single-strain probiotics are denoted in blue, multiple-strain probiotics are denoted in orange, and single- or multiple-strain probiotics are denoted in red. *B.*, *Bifidobacterium*; *L. acidophilus*, *Lactobacillus acidophilus*; LOHS, length of hospital stay; *L. reuteri*, *Limosilactobacillus reuteri*; *L. rhamnosus*, *Lacticaseibacillus rhamnosus*; LGG, *Lacticaseibacillus rhamnosus* GG; PMW, post-menstrual week.

**Supplemental Figure 2.** Probiotic exposures and gut microbial diversity in preterm infants. (A) LOESS fits (95% CIs shaded in grey) over time for Simpson index. (B) Forest plots of the association between probiotics and Simpson index. (C) Subgroup analysis of Simpson index between probiotics and control preterm infants by probiotic types. (D) Sensitivity analysis for multivariate analysis of Simpson index via excluding a study at a time and then pooling the remaining studies. (E) Funnel plot for assessing potential publication bias in the Simpson index before applying the trim-and-fill method. (F) Funnel plot for assessing potential publication bias in the Simpson index after applying the trim-and-fill method. *B. infantis*, *Bifidobacterium infantis*; *L. acidophilus*, *Lactobacillus acidophilus*; LOESS, locally estimated scatterplot smoothing; *L. reuteri*, *Limosilactobacillus reuteri*; *L. rhamnosus*, *Lacticaseibacillus rhamnosus*; PMW, post-menstrual week; SMD, standardised mean difference.

**Supplemental Figure 3.** Probiotic exposures and gut microbial diversity in preterm infants. (A) LOESS fits (95% CIs shaded in grey) over time for Richness index. (B) Forest plots of the association between probiotics and Richness index. (C) Subgroup analysis of Richness index between probiotics and control preterm infants by probiotic types. (D) Sensitivity analysis for multivariate analysis of Richness index via excluding a study at a time and then pooling the remaining studies. (E) Funnel plot for assessing potential publication bias in the Richness index before applying the trim-and-fill method. (F) Funnel plot for assessing potential publication bias in the Richness index after applying the trim-and-fill method. *B. infantis*, *Bifidobacterium infantis*; *L. acidophilus*, *Lactobacillus acidophilus*; LOESS, locally estimated scatterplot smoothing; *L. reuteri*, *Limosilactobacillus reuteri*; *L. rhamnosus*, *Lacticaseibacillus rhamnosus*; PMW, post-menstrual week; SMD, standardised mean difference.

**Supplemental Figure 4.** Probiotic exposures and gut microbial diversity in preterm infants. (A) LOESS fits (95% CIs shaded in grey) over time for Chao1 index. (B) Forest plots of the association between probiotics and Chao1 index. (C) Subgroup analysis of Chao1 index between probiotics and control preterm infants by probiotic types. (D) Sensitivity analysis for multivariate analysis of Chao1 index via excluding a study at a time and then pooling the remaining studies. (E) Funnel plot for assessing potential publication bias in the Chao1 index before applying the trim-and-fill method. (F) Funnel plot for assessing potential publication bias in the Chao1 index after applying the trim-and-fill method. *B. infantis*, *Bifidobacterium infantis*; *L. acidophilus*, *Lactobacillus acidophilus*; LOESS, locally estimated scatterplot smoothing; *L. reuteri*, *Limosilactobacillus reuteri*; *L. rhamnosus*, *Lacticaseibacillus rhamnosus*; PMW, post-menstrual week; SMD, standardised mean difference.

**Supplemental Figure 5.** The association between probiotics and publication bias of gut microbial diversity in preterm infants. Publication bias of (A) Shannon indexes, (B) Simpson indexes, (C) Richness indexes, (D) Chao1 indexes in probiotic and control preterm infants in each study after application of the trim-and-fill method, and the pooled effects across five included studies (meta-analysis) with 95% CI. SMD, standardised mean difference.

**Supplemental Figure 6.** Probiotic exposures and gut microbiome structure in preterm infants. (A) Scree plot visualising the percentage of variation explained by each principal component. (B) UMAP dimensionality reduction analysis of the top 13 PCs identified by the scree plot. umap, uniform manifold approximation and projection.

**Supplemental Figure 7.** Relative abundances of the dominant taxa at the phylum level in three independent cohorts. For display and clarity, only the top 10 most abundant taxa are shown. *B. infantis*, *Bifidobacterium infantis*; *L. acidophilus*, *Lactobacillus acidophilus*; *L. reuteri*, *Limosilactobacillus reuteri*; *L. rhamnosus*, *Lacticaseibacillus rhamnosus*; PMW, post-menstrual week.

**Supplemental Figure 8.** Relative abundances of the dominant taxa at the family level in three independent cohorts. For display and clarity, only the top 10 most abundant taxa are shown. *B. infantis*, *Bifidobacterium infantis*; *L. acidophilus*, *Lactobacillus acidophilus*; *L. reuteri*, *Limosilactobacillus reuteri*; *L. rhamnosus*, *Lacticaseibacillus rhamnosus*; PMW, post-menstrual week.

**Supplemental Figure 9.** Relative abundances of the dominant taxa at the genus level in three independent cohorts. For display and clarity, only the top 10 most abundant taxa are shown. *B. infantis*, *Bifidobacterium infantis*; *L. acidophilus*, *Lactobacillus acidophilus*; *L. reuteri*, *Limosilactobacillus reuteri*; *L. rhamnosus*, *Lacticaseibacillus rhamnosus*; PMW, post-menstrual week.

**Supplemental Figure 10.** Probiotic exposures and gut bacterial MetaCyc pathway abundances in preterm infants. (A) Heatmap of all samples (n = 1437) showing the 347 metabolic pathways with significant changes related to probiotics use in each cohort and meta-analysis. (B) Effect sizes were calculated by SMD in CLR using a random-effect meta-analysis method. Differential abundances of metabolic pathways detected in at least 5% of the samples in that study was corrected for FDR using the Kruskal-Wallis test. *B. infantis*, *Bifidobacterium infantis*; CLR, centered log-ratio; FDR, false discovery rate; *L. acidophilus*, *Lactobacillus acidophilus*; *L. reuteri*, *Limosilactobacillus reuteri*; *L. rhamnosus*, *Lacticaseibacillus rhamnosus*.

**Supplemental Table 1.** Search strategy.

| **Databases**  **(11/22/2023)** | | **Search strategy** | **Number of references** |
| --- | --- | --- | --- |
| **Web of Science** | TS=(Infant, Premature OR Infants, Premature OR Premature Infant OR Preterm Infants OR Infant, Preterm OR Infants, Preterm OR Preterm Infant OR Premature Infants OR Neonatal Prematurity OR Prematurity, Neonatal) AND TS=(Probiotics OR Probiotic OR Lactobacillus OR Bifidobacterium) AND TS=(Randomized Controlled Trial OR Randomized Controlled Trial* OR Randomized Double-Blind Placebo-Controlled Stud*) | | 196 |
| **PubMed** | ((((((((((("Infant, Premature"[Mesh]) OR (Infants, Premature[Title/Abstract])) OR (Premature Infant[Title/Abstract])) OR (Preterm Infants[Title/Abstract])) OR (Infant, Preterm[Title/Abstract])) OR (Infants, Preterm[Title/Abstract])) OR (Preterm Infant[Title/Abstract])) OR (Premature Infants[Title/Abstract])) OR (Neonatal Prematurity[Title/Abstract])) OR (Prematurity, Neonatal[Title/Abstract])) AND (((("Probiotics"[Mesh]) OR (Probiotic[Title/Abstract])) OR (Lactobacillus[Title/Abstract])) OR (Bifidobacterium[Title/Abstract]))) AND ((("Randomized Controlled Trial" [Publication Type]) OR (Randomized Controlled Trial*[Title/Abstract])) OR (Randomized Double-Blind Placebo-Controlled Stud*[Title/Abstract])) | | 182 |
| **Cochrane** | #1 (Infant, Premature OR Infants, Premature OR Premature Infant OR Preterm Infants OR Infant, Preterm OR Infants, Preterm OR Preterm Infant OR Premature Infants OR Neonatal Prematurity OR Prematurity, Neonatal):ab,ti,kw  #2 (Probiotics OR Probiotic OR Lactobacillus OR Bifidobacterium):ab,ti,kw  #3 (Randomized Controlled Trial OR Randomized Controlled Trial* OR Randomized Double-Blind Placebo-Controlled Stud*):ab,ti,kw  #4 #1 AND #2 AND #3 | | 229 |
| **EMBASE** | ((('infant,'/exp OR infant,) AND ('premature'/exp OR premature)) OR ('infants, premature':ab,ti OR 'premature infant':ab,ti OR 'preterm infants':ab,ti OR 'infant, preterm':ab,ti OR 'infants, preterm':ab,ti OR 'preterm infant':ab,ti OR 'premature infants':ab,ti OR 'neonatal prematurity':ab,ti OR 'prematurity, neonatal':ab,ti)) AND (('probiotics'/exp OR probiotics) OR ('probiotic':ab,ti OR 'lactobacillus':ab,ti OR 'bifidobacterium':ab,ti)) AND ((randomized AND controlled AND ('trial'/exp OR trial)) OR ('randomized controlled trial*':ab,ti OR 'randomized double-blind placebo-controlled stud*':ab,ti)) | | 397 |

Mesh, medical subject headings.

**Supplemental Table 2.** Full-text studies excluded.

| **Title** | **Exclusion reason** | **DOI** |
| --- | --- | --- |
| LIVE VS HEAT-INACTIVATED PROBIOTICS in PRETERM INFANTS (THE PROPARA STUDY)- A PILOT RANDOMIZED TRIAL | Inappropriate study type | 10.1111/jpc.16356 |
| Bifidobacterium Supplementation of Colostrum and Breast Milk Enhances Weight Gain and Metabolic Responses Associated with Microbiota Establishment in Very-Preterm Infants | Inappropriate data type | 10.1159/000502935 |
| Effect of early administration of probiotics on gut microflora and feeding in pre-term infants: a randomized controlled trial | Inappropriate data type | 10.3109/14767058.2016.1163674 |
| Effects of oral probiotic supplementation on gut Lactobacillus and Bifidobacterium populations and the clinical status of low-birth-weight preterm neonates: A multicenter randomized, double-blind, placebo-controlled trial | Inappropriate data type | 10.2147/IDR.S166348 |
| Efficacy of Bifidobacterium longum, B. infantis and Lactobacillus acidophilus probiotics to prevent gut dysbiosis in preterm infants of 28+0-32+6 weeks of gestation: a randomised, placebo-controlled, double-blind, multicentre trial: the PRIMAL Clinical Study protocol | Inappropriate data type | 10.1136/bmjopen-2019-032617 |
| Multi-strain probiotics for extremely preterm infants: a randomized controlled trial | Inappropriate data type | 10.1038/s41390-022-02004-z |
| Randomized, Double-blind, Placebo-controlled Study to Assess the Effect of Two Probiotics on the Preterms' Gut Microbiota | Inappropriate data type | 10.1097/MPG.0000000000003427 |
| The effect of probiotic supplementation on the gut microbiota of preterm infants | Inappropriate data type | 10.1099/jmm.0.001403 |
| Effect of Saccharomyces boulardii and Mode of Delivery on the Early Development of the Gut Microbial Community in Preterm Infants | Inappropriate data type | 10.1371/journal.pone.0150306 |
| Gut microbiota of preterm infants supplemented with probiotics: sub-study of the ProPrems trial | Inappropriate data type | 10.1186/s12866-018-1326-1 |
| A comparison of two probiotic strains of bifidobacteria in premature infants | Inappropriate data type | 10.1016/j.jpeds.2013.07.017 |
| Effect of single versus multistrain probiotic in extremely preterm infants: a randomised trial | Unavailable sequencing data | 10.1136/bmjgast-2021-000811 |
| Effects of Probiotics on Gut Microbiomes of Extremely Preterm Infants in the Neonatal Intensive Care Unit: A Prospective Cohort Study | Unavailable sequencing data | 10.3390/nu14153239 |
| **Title** | **Exclusion reason** | **DOI** |
| The Microbiome of Infants Recruited to a Randomised Placebo-controlled Probiotic Trial (PiPS Trial) | Unavailable sequencing data | 10.1016/j.ebiom.2017.05.019 |
| The effect of early probiotic exposure on the preterm infant gut microbiome development | Unavailable sequencing data | 10.1080/19490976.2021.1951113 |
| Preterm Infant Fecal Microbiota and Metabolite Profiles Are Modulated in a Probiotic Specific Manner | Unavailable sequencing data | 10.1097/MPG.0000000000003570 |
| The preterm gut microbiota and administration routes of different probiotics: a randomized controlled trial | Unavailable sequencing data | 10.1038/s41390-023-02560-y |
| Persistence of Suspected Probiotic Organisms in Preterm Infant Gut Microbiota Weeks After Probiotic Supplementation in the NICU | Unavailable sequencing data | 10.3389/fmicb.2020.574137 |
| Correction: efficacy of Bifidobacterium animalis subsp. lactis (BB-12), B. infantis and Lactobacillus acidophilus (La-5) probiotics to prevent gut dysbiosis in preterm infants of 28+0-32+6 weeks of gestation: a randomised, placebo-controlled, double-blind, multicentre trial: the PRIMAL Clinical Study Protocol | Unavailable sequencing data | 10.1136/bmjopen-2019-032617corr1 |
| Effects of a formula with a probiotic Bifidobacterium lactis Supplement on the gut microbiota of low birth weight infants | Unavailable metadata | 10.1007/s00394-019-02006-4 |
| Influence of probiotic supplementation on the developing microbiota in human preterm neonates | Unavailable metadata | 10.1080/19490976.2020.1826747 |
| Supplementation with a probiotic mixture accelerates gut microbiome maturation and reduces intestinal inflammation in extremely preterm infants | Unavailable metadata | 10.1016/j.chom.2022.04.005 |

**Supplemental Table 3.** Significant taxa obtained by Kruskal-Wallis test in pooled data, and meta-analysis of the differential taxa.

| **Taxa^1^** | **Test-Statistic** | ***p*-value** | **FDR_*p*** | **Control_mean** | **Probiotics_mean** |
| --- | --- | --- | --- | --- | --- |
| Lactobacillaceae | 235.4270894 | 3.91E-53 | 1.72E-51 | 0.039402249 | 0.092246282 |
| Lactobacillus | 213.0261712 | 3.00E-48 | 6.61E-47 | 0.039065657 | 0.087698481 |
| Bifidobacterium | 164.2331546 | 1.35E-37 | 1.97E-36 | 0.033888856 | 0.205279515 |
| Bifidobacteriaceae | 162.0137898 | 4.11E-37 | 4.52E-36 | 0.034068541 | 0.205292328 |
| Actinobacteria | 75.22170104 | 4.21E-18 | 3.70E-17 | 0.049712037 | 0.213042151 |
| Bacillaceae | 74.59567654 | 5.78E-18 | 4.24E-17 | 0.000171931 | 0.003964409 |
| Enterobacteriaceae | 66.83495356 | 2.95E-16 | 1.86E-15 | 0.318344225 | 0.157079981 |
| Klebsiella | 58.21065182 | 2.36E-14 | 1.30E-13 | 0.036912981 | 0.007414453 |
| Xanthomonadaceae | 53.35945296 | 2.78E-13 | 1.23E-12 | 0.002979276 | 0.005929629 |
| Stenotrophomonas | 53.35114926 | 2.79E-13 | 1.23E-12 | 0.002979276 | 0.005914452 |
| Proteobacteria | 52.15902413 | 5.12E-13 | 2.05E-12 | 0.372148752 | 0.229371842 |
| Ralstonia | 50.93441721 | 9.55E-13 | 3.50E-12 | 8.78E-05 | 0.008302263 |
| Burkholderiaceae | 49.43568495 | 2.05E-12 | 6.94E-12 | 0.000668352 | 0.011439325 |
| Propionibacteriaceae | 45.12717357 | 1.85E-11 | 5.48E-11 | 0.009244684 | 0.002941431 |
| Cutibacterium | 45.10568827 | 1.87E-11 | 5.48E-11 | 0.009244201 | 0.002935271 |
| Sphingomonadaceae | 41.39351827 | 1.24E-10 | 3.42E-10 | 7.35E-05 | 0.000599356 |
| Sphingomonas | 40.40521001 | 2.06E-10 | 5.34E-10 | 5.91E-05 | 0.000508296 |
| Bacteroidetes | 39.35742489 | 3.53E-10 | 8.63E-10 | 0.026252822 | 0.039206012 |
| Lactococcus | 34.5907149 | 4.07E-09 | 9.02E-09 | 0.001191078 | 0.004011729 |
| Xanthobacteraceae | 34.57654288 | 4.10E-09 | 9.02E-09 | 1.30E-05 | 0.00039468 |
| Pseudomonadaceae | 32.5526479 | 1.16E-08 | 2.32E-08 | 0.004599183 | 0.00478626 |
| Pseudomonas | 32.5526479 | 1.16E-08 | 2.32E-08 | 0.004599183 | 0.00478626 |
| Caulobacteraceae | 31.35063776 | 2.15E-08 | 4.12E-08 | 1.03E-05 | 9.42E-05 |
| Veillonella | 28.19882397 | 1.09E-07 | 2.01E-07 | 0.040298939 | 0.02312287 |
| Veillonellaceae | 26.72829489 | 2.34E-07 | 4.12E-07 | 0.041856778 | 0.023932897 |
| Clostridium sensu stricto 1 | 26.49604028 | 2.64E-07 | 4.47E-07 | 0.014848832 | 0.010187273 |
| **Taxa^1^** | **Test-Statistic** | ***p*-value** | **FDR_*p*** | **Control_mean** | **Probiotics_mean** |
| Clostridiaceae 1 | 24.50966505 | 7.39E-07 | 1.20E-06 | 0.020813636 | 0.011227942 |
| Brevundimonas | 19.27302021 | 1.13E-05 | 1.78E-05 | 1.01E-05 | 4.54E-05 |
| Corynebacterium 1 | 15.9001626 | 6.68E-05 | 0.000101311 | 0.003529208 | 0.002753669 |
| Corynebacteriaceae | 15.13046448 | 0.0001 | 0.000147152 | 0.003720874 | 0.003321448 |
| Anaerococcus | 14.2682714 | 0.000159 | 0.000224988 | 0.001113401 | 0.000479836 |
| Finegoldia | 13.19018613 | 0.000281 | 0.000386951 | 0.001391879 | 0.000406616 |
| Pasteurellaceae | 12.98161614 | 0.000315 | 0.000419419 | 0.012512314 | 0.008599479 |
| Clostridiales Family XI | 11.7266598 | 0.000616 | 0.00079732 | 0.004862163 | 0.001009518 |
| Moraxellaceae | 11.45042935 | 0.000715 | 0.000898574 | 0.002672768 | 0.005254095 |
| Haemophilus | 11.24657455 | 0.000798 | 0.000974968 | 0.011773607 | 0.008544521 |
| Acinetobacter | 10.26261322 | 0.001358 | 0.001614363 | 0.002671923 | 0.0052247 |
| Eggerthellaceae | 7.800010265 | 0.005225 | 0.00604953 | 0.000362133 | 0.000198375 |
| Firmicutes | 5.064499318 | 0.024421 | 0.027551625 | 0.54839092 | 0.510633342 |
| Enterococcus | 2.227930713 | 0.135535 | 0.149088785 | 0.155158347 | 0.14595012 |
| Enterococcaceae | 1.80147821 | 0.179534 | 0.192670515 | 0.155158347 | 0.14597996 |
| Escherichia-Shigella | 1.101052577 | 0.294035 | 0.308036901 | 0.061407757 | 0.055153345 |
| Streptococcus | 0.592431864 | 0.44148 | 0.451747253 | 0.031401516 | 0.020764895 |
| Streptococcaceae | 0.025906094 | 0.87213 | 0.872129865 | 0.032592594 | 0.024776624 |

| **Taxa^2^** | **Pooled estimate (SMD in CLR)** | **95% Pooled lower limit** | **95% Pooled upper limit** | **Pooled *p*-value** |
| --- | --- | --- | --- | --- |
| p_Actinobacteria | 0.52 | 0.41 | 0.62 | 4.19E-22 |
| p_Bacteroidetes | 0.25 | 0.14 | 0.35 | 2.81E-06 |
| p_Firmicutes | -0.12 | -0.22 | -0.01 | 0.029033686 |
| p_Proteobacteria | -0.43 | -0.54 | -0.33 | 5.36E-16 |
| f_Bacillaceae | 0.37 | 0.27 | 0.48 | 2.27E-12 |
| f_Bifidobacteriaceae | 0.81 | 0.70 | 0.91 | 8.12E-49 |
| f_Burkholderiaceae | 0.33 | 0.23 | 0.44 | 3.65E-10 |
| f_Caulobacteraceae | 0.21 | 0.11 | 0.31 | 7.23E-05 |
| f_Clostridiaceae 1 | -0.29 | -0.39 | -0.18 | 7.73E-08 |
| f_Clostridiales Family XI | -0.26 | -0.37 | -0.16 | 6.27E-07 |
| f_Corynebacteriaceae | -0.15 | -0.25 | -0.04 | 0.006031297 |
| f_Eggerthellaceae | -0.16 | -0.26 | -0.05 | 0.002943943 |
| f_Enterobacteriaceae | -0.47 | -0.57 | -0.36 | 1.74E-18 |
| f_Enterococcaceae | -0.06 | -0.17 | 0.04 | 0.225014229 |
| f_Lactobacillaceae | 0.77 | 0.66 | 0.87 | 1.62E-44 |
| f_Moraxellaceae | 0.15 | 0.04 | 0.25 | 0.005344486 |
| f_Pasteurellaceae | -0.15 | -0.26 | -0.05 | 0.003965274 |
| f_Propionibacteriaceae | -0.30 | -0.40 | -0.19 | 2.32E-08 |
| f_Pseudomonadaceae | 0.21 | 0.11 | 0.31 | 6.90E-05 |
| f_Sphingomonadaceae | 0.27 | 0.16 | 0.37 | 5.49E-07 |
| f_Streptococcaceae | 0.00 | -0.10 | 0.11 | 0.947674839 |
| f_Veillonellaceae | -0.34 | -0.45 | -0.24 | 9.09E-11 |
| f_Xanthobacteraceae | 0.26 | 0.15 | 0.36 | 1.12E-06 |
| f_Xanthomonadaceae | 0.28 | 0.18 | 0.39 | 9.18E-08 |
| g_Acinetobacter | 0.14 | 0.04 | 0.25 | 0.006859077 |
| g_Anaerococcus | -0.21 | -0.31 | -0.10 | 9.35E-05 |
| g_Bifidobacterium | 0.81 | 0.71 | 0.92 | 1.64E-49 |
| g_Brevundimonas | 0.15 | 0.04 | 0.25 | 0.005306377 |
| **Taxa^2^** | **Pooled estimate (SMD in CLR)** | **95% Pooled lower limit** | **95% Pooled upper limit** | **Pooled *p*-value** |
| g_Clostridium sensu stricto 1 | -0.27 | -0.37 | -0.16 | 5.80E-07 |
| g_Corynebacterium 1 | -0.16 | -0.26 | -0.06 | 0.00257463 |
| g_Cutibacterium | -0.30 | -0.40 | -0.19 | 2.33E-08 |
| g_Enterococcus | -0.07 | -0.18 | 0.03 | 0.176209956 |
| g_Escherichia-Shigella | 0.03 | -0.07 | 0.14 | 0.514748008 |
| g_Finegoldia | -0.24 | -0.35 | -0.14 | 4.33E-06 |
| g_Haemophilus | -0.15 | -0.25 | -0.04 | 0.005597449 |
| g_Klebsiella | -0.33 | -0.43 | -0.22 | 9.15E-10 |
| g_Lactobacillus | 0.71 | 0.61 | 0.82 | 3.31E-39 |
| g_Lactococcus | 0.21 | 0.11 | 0.32 | 5.50E-05 |
| g_Pseudomonas | 0.21 | 0.11 | 0.31 | 6.90E-05 |
| g_Ralstonia | 0.37 | 0.26 | 0.47 | 5.85E-12 |
| g_Sphingomonas | 0.26 | 0.16 | 0.36 | 9.04E-07 |
| g_Stenotrophomonas | 0.28 | 0.18 | 0.39 | 9.02E-08 |
| g_Streptococcus | -0.06 | -0.16 | 0.05 | 0.277761446 |
| g_Veillonella | -0.35 | -0.45 | -0.24 | 6.40E-11 |

CLR, centered log-ratio; FDR, false discovery rate; SMD, standardised mean difference.

**Table Footnotes**

^1^ Kruskal-Wallis test results for 44 common differential taxa included in the meta-analysis.

^2^ Meta-analysis of all three included studies for 44 common differential taxa.

**Supplemental Table 4.** The result of DMM transitions over time.

| **Control** | PGCT-1@  0-9 d | PGCT-2@  0-9 d | PGCT-1@  10-29 d | PGCT-2@  10-29 d | PGCT-1@  30-49 d | PGCT-2@  30-49 d | PGCT-3@  30-49 d | PGCT-1@  50-99 d | PGCT-2@  50-99 d |
| --- | --- | --- | --- | --- | --- | --- | --- | --- | --- |
| PGCT-1@0-9 d | 0 | 0 | 0.337209302 | 0.279069767 | 0 | 0 | 0 | 0 | 0 |
| PGCT-2@0-9 d | 0 | 0 | 0.058139535 | 0.325581395 | 0 | 0 | 0 | 0 | 0 |
| PGCT-1@10-29 d | 0 | 0 | 0 | 0 | 0.171428571 | 0.228571429 | 0 | 0 | 0 |
| PGCT-2@10-29 d | 0 | 0 | 0 | 0 | 0.114285714 | 0.485714286 | 0 | 0 | 0 |
| PGCT-1@30-49 d | 0 | 0 | 0 | 0 | 0 | 0 | 0 | 0.125 | 0.125 |
| PGCT-2@30-49 d | 0 | 0 | 0 | 0 | 0 | 0 | 0 | 0.041666667 | 0.666666667 |
| PGCT-3@30-49 d | 0 | 0 | 0 | 0 | 0 | 0 | 0 | 0 | 0.041666667 |
| PGCT-1@50-99 d | 0 | 0 | 0 | 0 | 0 | 0 | 0 | 0 | 0 |
| PGCT-2@50-99 d | 0 | 0 | 0 | 0 | 0 | 0 | 0 | 0 | 0 |

| **Probiotics** | PGCT-1@  0-9 d | PGCT-3@  0-9 d | PGCT-1@  10-29 d | PGCT-2@  10-29 d | PGCT-3@  10-29 d | PGCT-1@  30-49 d | PGCT-2@  30-49 d | PGCT-3@  30-49 d | PGCT-1@  50-99 d | PGCT-2@  50-99 d |
| --- | --- | --- | --- | --- | --- | --- | --- | --- | --- | --- |
| PGCT-1@0-9 d | 0 | 0 | 0.807692308 | 0.076923077 | 0.019230769 | 0 | 0 | 0 | 0 | 0 |
| PGCT-3@0-9 d | 0 | 0 | 0.019230769 | 0 | 0.076923077 | 0 | 0 | 0 | 0 | 0 |
| PGCT-1@10-29 d | 0 | 0 | 0 | 0 | 0 | 0.46 | 0.3 | 0 | 0 | 0 |
| PGCT-2@10-29 d | 0 | 0 | 0 | 0 | 0 | 0.02 | 0.04 | 0.02 | 0 | 0 |
| PGCT-3@10-29 d | 0 | 0 | 0 | 0 | 0 | 0.02 | 0 | 0.14 | 0 | 0 |
| PGCT-1@30-49 d | 0 | 0 | 0 | 0 | 0 | 0 | 0 | 0 | 0.416666667 | 0.291666667 |
| PGCT-2@30-49 d | 0 | 0 | 0 | 0 | 0 | 0 | 0 | 0 | 0.083333333 | 0.208333333 |
| PGCT-3@30-49 d | 0 | 0 | 0 | 0 | 0 | 0 | 0 | 0 | 0 | 0 |
| PGCT-1@50-99 d | 0 | 0 | 0 | 0 | 0 | 0 | 0 | 0 | 0 | 0 |
| PGCT-2@50-99 d | 0 | 0 | 0 | 0 | 0 | 0 | 0 | 0 | 0 | 0 |

PGCT, preterm gut community type.

**Supplemental Table 5.** Significant metabolic pathways obtained by Kruskal-Wallis test in pooled data, and meta-analysis of the differential pathways.

| **Pathways^1^** | **Test-Statistic** | | ***p*-value** | **FDR_*p*** | | **Control_mean** | | **Probiotics_mean** | |
| --- | --- | --- | --- | --- | --- | --- | --- | --- | --- |
| Bifidobacterium shunt | 320.0422565 | | 1.42E-71 | 4.92E-69 | | 0.001225887 | | 0.004282216 | |
| heterolactic fermentation | 287.500157 | | 1.74E-64 | 3.02E-62 | | 0.001099635 | | 0.003088172 | |
| peptidoglycan biosynthesis V (beta-lactam resistance) | 261.5715641 | | 7.80E-59 | 9.02E-57 | | 0.000757632 | | 0.001835708 | |
| L-lysine biosynthesis VI | 113.4783683 | | 1.70E-26 | 1.47E-24 | | 0.005639914 | | 0.007502775 | |
| L-lysine biosynthesis III | 106.9345383 | | 4.60E-25 | 3.19E-23 | | 0.007289845 | | 0.008847112 | |
| peptidoglycan biosynthesis III (mycobacteria) | 89.61478483 | | 2.89E-21 | 1.67E-19 | | 0.006057983 | | 0.007764267 | |
| adenosine ribonucleotides de novo biosynthesis | 87.90484765 | | 6.87E-21 | 3.40E-19 | | 0.007668246 | | 0.009222727 | |
| UMP biosynthesis | 86.57102103 | | 1.35E-20 | 5.85E-19 | | 0.007316343 | | 0.009020254 | |
| chorismate biosynthesis from 3-dehydroquinate | 86.07671229 | | 1.73E-20 | 6.67E-19 | | 0.006706701 | | 0.007848623 | |
| inosine-5'-phosphate biosynthesis I | 84.59057097 | | 3.67E-20 | 1.27E-18 | | 0.006598223 | | 0.00802269 | |
| superpathway of adenosine nucleotides de novo biosynthesis I | 82.36021002 | | 1.13E-19 | 3.58E-18 | | 0.008108438 | | 0.009683277 | |
| superpathway of pyrimidine deoxyribonucleotides de novo biosynthesis | 81.5769172 | | 1.69E-19 | 4.87E-18 | | 0.00294794 | | 0.00202751 | |
| peptidoglycan biosynthesis I (meso-diaminopimelate containing) | 81.14724461 | | 2.10E-19 | 5.59E-18 | | 0.006966311 | | 0.008410361 | |
| superpathway of adenosine nucleotides de novo biosynthesis II | 80.37630812 | | 3.09E-19 | 7.67E-18 | | 0.007798038 | | 0.009318127 | |
| peptidoglycan biosynthesis IV (Enterococcus faecium) | 79.60267988 | | 4.58E-19 | 9.93E-18 | | 0.003136246 | | 0.005060502 | |
| UDP-N-acetylmuramoyl-pentapeptide biosynthesis I (meso-diaminopimelate containing) | 79.68856546 | | 4.38E-19 | 9.93E-18 | | 0.007092153 | | 0.008554229 | |
| superpathway of heme biosynthesis from uroporphyrinogen-III | 79.31071002 | | 5.31E-19 | 1.08E-17 | | 0.001929017 | | 0.001100665 | |
| inosine-5'-phosphate biosynthesis III | 77.78219865 | | 1.15E-18 | 2.22E-17 | | 0.006155521 | | 0.007674921 | |
| ppGpp biosynthesis | 77.36976828 | | 1.42E-18 | 2.45E-17 | | 0.002243833 | | 0.001349459 | |
| superpathway of demethylmenaquinol-8 biosynthesis | 77.28268224 | | 1.48E-18 | 2.45E-17 | | 0.002389815 | | 0.00151097 | |
| superpathway of histidine, purine, and pyrimidine biosynthesis | 77.45359373 | | 1.36E-18 | 2.45E-17 | | 0.003082876 | | 0.002176733 | |
| UDP-N-acetyl-D-glucosamine biosynthesis I | 76.30529496 | | 2.43E-18 | 3.83E-17 | | 0.006402271 | | 0.007840588 | |
| superpathway of menaquinol-8 biosynthesis I | 75.60401703 | | 3.47E-18 | 5.23E-17 | | 0.002343023 | | 0.001498037 | |
| **Pathways^1^** | **Test-Statistic** | | ***p*-value** | **FDR_*p*** | | **Control_mean** | | **Probiotics_mean** | |
| UDP-N-acetylmuramoyl-pentapeptide biosynthesis II (lysine-containing) | 74.67747188 | | 5.54E-18 | 8.01E-17 | | 0.007049247 | | 0.008464611 | |
| galactose degradation I (Leloir pathway) | 74.47193331 | | 6.15E-18 | 8.54E-17 | | 0.00608442 | | 0.008313979 | |
| pyruvate fermentation to acetate and lactate II | 74.0934732 | | 7.45E-18 | 9.94E-17 | | 0.006857342 | | 0.008705105 | |
| superpathway of pyridoxal 5'-phosphate biosynthesis and salvage | 73.6860639 | | 9.16E-18 | 1.18E-16 | | 0.002239505 | | 0.001379361 | |
| anhydromuropeptides recycling | 73.42123225 | | 1.05E-17 | 1.30E-16 | | 0.003941538 | | 0.002662057 | |
| 5-aminoimidazole ribonucleotide biosynthesis I | 73.28495637 | | 1.12E-17 | 1.34E-16 | | 0.007312985 | | 0.008695806 | |
| superpathway of L-isoleucine biosynthesis I | 72.93349498 | | 1.34E-17 | 1.55E-16 | | 0.00566854 | | 0.006762959 | |
| superpathway of thiamin diphosphate biosynthesis II | 72.51780826 | | 1.66E-17 | 1.85E-16 | | 0.002277706 | | 0.001471929 | |
| 5-aminoimidazole ribonucleotide biosynthesis II | 72.31409685 | | 1.84E-17 | 1.93E-16 | | 0.007222487 | | 0.008721438 | |
| superpathway of 5-aminoimidazole ribonucleotide biosynthesis | 72.31409685 | | 1.84E-17 | 1.93E-16 | | 0.007222487 | | 0.008721438 | |
| 4-hydroxyphenylacetate degradation | 71.82108263 | | 2.36E-17 | 2.38E-16 | | 0.001063033 | | 0.000502506 | |
| O-antigen building blocks biosynthesis (E. coli) | 71.78553816 | | 2.40E-17 | 2.38E-16 | | 0.002623719 | | 0.004211994 | |
| S-methyl-5-thio-alpha-D-ribose 1-phosphate degradation | 71.43187134 | | 2.87E-17 | 2.77E-16 | | 1.65E-06 | | 3.98E-05 | |
| S-adenosyl-L-methionine cycle I | 70.4024783 | | 4.84E-17 | 4.54E-16 | | 0.006411179 | | 0.007771121 | |
| 1,4-dihydroxy-2-naphthoate biosynthesis I | 69.67758628 | | 6.98E-17 | 6.38E-16 | | 0.004119896 | | 0.003185974 | |
| superpathway of menaquinol-10 biosynthesis | 69.29216674 | | 8.49E-17 | 7.19E-16 | | 0.002014042 | | 0.001175125 | |
| superpathway of menaquinol-6 biosynthesis I | 69.29216674 | | 8.49E-17 | 7.19E-16 | | 0.002014042 | | 0.001175125 | |
| superpathway of menaquinol-9 biosynthesis | 69.29216674 | | 8.49E-17 | 7.19E-16 | | 0.002014042 | | 0.001175125 | |
| superpathway of demethylmenaquinol-6 biosynthesis I | 68.94953995 | | 1.01E-16 | 8.15E-16 | | 0.002039273 | | 0.001176067 | |
| superpathway of demethylmenaquinol-9 biosynthesis | 68.94953995 | | 1.01E-16 | 8.15E-16 | | 0.002039273 | | 0.001176067 | |
| L-isoleucine biosynthesis I (from threonine) | 68.66514844 | | 1.17E-16 | 8.83E-16 | | 0.005764159 | | 0.007102235 | |
| L-valine biosynthesis | 68.66514844 | | 1.17E-16 | 8.83E-16 | | 0.005764159 | | 0.007102235 | |
| superpathway of L-threonine biosynthesis | 68.65982358 | | 1.17E-16 | 8.83E-16 | | 0.007028376 | | 0.007945117 | |
| ADP-L-glycero-beta-D-manno-heptose biosynthesis | 68.09598031 | | 1.56E-16 | 1.15E-15 | | 0.001840095 | | 0.001111467 | |
| dTDP-L-rhamnose biosynthesis I | 67.18822709 | | 2.47E-16 | 1.78E-15 | | 0.002665238 | | 0.004318996 | |
| pyridoxal 5'-phosphate biosynthesis I | 66.71558908 | | 3.14E-16 | 2.22E-15 | | 0.001705394 | | 0.00105412 | |
| superpathway of chorismate metabolism | 65.14993241 | | 6.94E-16 | 4.82E-15 | | 0.002122989 | | 0.001240706 | |
| guanosine deoxyribonucleotides de novo biosynthesis II | 64.97711548 | | 7.58E-16 | 5.06E-15 | | 0.008180112 | | 0.009701136 | |
| **Pathways^1^** | **Test-Statistic** | | ***p*-value** | **FDR_*p*** | | **Control_mean** | | **Probiotics_mean** | |
| adenosine deoxyribonucleotides de novo biosynthesis II | 64.97711548 | | 7.58E-16 | 5.06E-15 | | 0.008180112 | | 0.009701136 | |
| heme biosynthesis I (aerobic) | 64.32754224 | | 1.05E-15 | 6.90E-15 | | 0.003438637 | | 0.002645276 | |
| (5Z)-dodec-5-enoate biosynthesis | 63.1873029 | | 1.88E-15 | 1.21E-14 | | 0.00369468 | | 0.002523435 | |
| superpathway of menaquinol-7 biosynthesis | 62.38967301 | | 2.82E-15 | 1.78E-14 | | 0.003996507 | | 0.003182422 | |
| superpathway of menaquinol-11 biosynthesis | 62.25904628 | | 3.01E-15 | 1.80E-14 | | 0.004008025 | | 0.003198878 | |
| superpathway of menaquinol-12 biosynthesis | 62.25904628 | | 3.01E-15 | 1.80E-14 | | 0.004008025 | | 0.003198878 | |
| superpathway of menaquinol-13 biosynthesis | 62.25904628 | | 3.01E-15 | 1.80E-14 | | 0.004008025 | | 0.003198878 | |
| enterobacterial common antigen biosynthesis | 62.1008175 | | 3.26E-15 | 1.92E-14 | | 0.001686063 | | 0.000990438 | |
| superpathway of branched amino acid biosynthesis | 61.97675926 | | 3.48E-15 | 2.01E-14 | | 0.005532216 | | 0.006686318 | |
| superpathway of (Kdo)2-lipid A biosynthesis | 61.72878459 | | 3.94E-15 | 2.24E-14 | | 0.001774174 | | 0.001068579 | |
| superpathway of phylloquinol biosynthesis | 61.20103951 | | 5.15E-15 | 2.88E-14 | | 0.004012701 | | 0.003204844 | |
| L-isoleucine biosynthesis III | 60.01950552 | | 9.39E-15 | 5.04E-14 | | 0.005367988 | | 0.006507092 | |
| pyruvate fermentation to isobutanol (engineered) | 60.00865911 | | 9.44E-15 | 5.04E-14 | | 0.00709661 | | 0.008753946 | |
| superpathway of beta-D-glucuronide and D-glucuronate degradation | 60.0561284 | | 9.22E-15 | 5.04E-14 | | 0.002386248 | | 0.001544205 | |
| heme biosynthesis II (anaerobic) | 59.89139863 | | 1.00E-14 | 5.27E-14 | | 0.004242785 | | 0.00336196 | |
| polymyxin resistance | 59.78210127 | | 1.06E-14 | 5.49E-14 | | 0.001479056 | | 0.000847254 | |
| methylphosphonate degradation I | 59.18109958 | | 1.44E-14 | 7.34E-14 | | 0.001858088 | | 0.001086883 | |
| dTDP-N-acetylthomosamine biosynthesis | 58.68457411 | | 1.85E-14 | 9.31E-14 | | 0.001819264 | | 0.00113287 | |
| CDP-diacylglycerol biosynthesis I | 57.10350284 | | 4.13E-14 | 1.99E-13 | | 0.00780449 | | 0.008972941 | |
| CDP-diacylglycerol biosynthesis II | 57.10350284 | | 4.13E-14 | 1.99E-13 | | 0.00780449 | | 0.008972941 | |
| enterobactin biosynthesis | 57.13409008 | | 4.07E-14 | 1.99E-13 | | 0.002126163 | | 0.001391593 | |
| superpathway of pyrimidine nucleobases salvage | 56.53744592 | | 5.51E-14 | 2.62E-13 | | 0.008357015 | | 0.009806451 | |
| superpathway of D-glucarate and D-galactarate degradation | 56.34967419 | | 6.07E-14 | 2.81E-13 | | 0.001580094 | | 0.000921109 | |
| D-galactarate degradation I | 56.34967419 | | 6.07E-14 | 2.81E-13 | | 0.001580094 | | 0.000921109 | |
| aerobactin biosynthesis | 56.30830812 | | 6.20E-14 | 2.83E-13 | | 0.000349804 | | 0.000154822 | |
| L-isoleucine biosynthesis II | 55.49818953 | | 9.35E-14 | 4.22E-13 | | 0.006046425 | | 0.00718878 | |
| D-glucarate degradation I | 55.27102147 | | 1.05E-13 | 4.67E-13 | | 0.001572292 | | 0.000923378 | |
| NAD salvage pathway II | 55.19292823 | | 1.09E-13 | 4.80E-13 | | 0.001980476 | | 0.001274697 | |
| **Pathways^1^** | **Test-Statistic** | | ***p*-value** | **FDR_*p*** | | **Control_mean** | | **Probiotics_mean** | |
| superpathway of ornithine degradation | 54.69244595 | | 1.41E-13 | 6.11E-13 | | 0.00170341 | | 0.000946875 | |
| tetrapyrrole biosynthesis II (from glycine) | 54.47137487 | | 1.58E-13 | 6.76E-13 | | 0.003913192 | | 0.003091146 | |
| L-arginine degradation II (AST pathway) | 53.6103821 | | 2.44E-13 | 1.03E-12 | | 0.001605907 | | 0.000984408 | |
| biotin biosynthesis II | 53.60400956 | | 2.45E-13 | 1.03E-12 | | 0.000985577 | | 0.00046265 | |
| oleate biosynthesis IV (anaerobic) | 52.88095703 | | 3.54E-13 | 1.46E-12 | | 0.003453175 | | 0.00233265 | |
| chorismate biosynthesis I | 52.42427715 | | 4.47E-13 | 1.83E-12 | | 0.006747232 | | 0.007452667 | |
| L-lysine biosynthesis I | 52.03615014 | | 5.45E-13 | 2.20E-12 | | 0.003963966 | | 0.00506188 | |
| palmitoleate biosynthesis I (from (5Z)-dodec-5-enoate) | 51.73350818 | | 6.36E-13 | 2.49E-12 | | 0.003280636 | | 0.002211212 | |
| superpathway of fatty acid biosynthesis initiation (E. coli) | 51.72341046 | | 6.39E-13 | 2.49E-12 | | 0.002928798 | | 0.001958214 | |
| biotin biosynthesis I | 51.75480675 | | 6.29E-13 | 2.49E-12 | | 0.002708634 | | 0.001819703 | |
| glycolysis III (from glucose) | 51.46567038 | | 7.29E-13 | 2.81E-12 | | 0.00751616 | | 0.008574318 | |
| superpathway of aromatic amino acid biosynthesis | 50.98650309 | | 9.30E-13 | 3.55E-12 | | 0.007040106 | | 0.007791728 | |
| 8-amino-7-oxononanoate biosynthesis I | 50.94941196 | | 9.48E-13 | 3.57E-12 | | 0.002754094 | | 0.001853458 | |
| preQ0 biosynthesis | 50.67630503 | | 1.09E-12 | 4.06E-12 | | 0.004020108 | | 0.003228591 | |
| Kdo transfer to lipid IVA III (Chlamydia) | 49.9194039 | | 1.60E-12 | 5.91E-12 | | 0.001980414 | | 0.001316392 | |
| thiazole biosynthesis I (E. coli) | 49.30517597 | | 2.19E-12 | 8.00E-12 | | 0.00257038 | | 0.001785354 | |
| L-isoleucine biosynthesis IV | 49.25952913 | | 2.24E-12 | 8.11E-12 | | 0.003672314 | | 0.002774939 | |
| fatty acid beta-oxidation I | 49.0847915 | | 2.45E-12 | 8.77E-12 | | 0.003500593 | | 0.002392196 | |
| protocatechuate degradation I (meta-cleavage pathway) | 48.85083268 | | 2.76E-12 | 9.78E-12 | | 2.16E-06 | | 1.90E-05 | |
| superpathway of glycolysis and Entner-Doudoroff | 48.81541493 | | 2.81E-12 | 9.86E-12 | | 0.002857061 | | 0.002071576 | |
| guanosine nucleotides degradation III | 48.55295866 | | 3.21E-12 | 1.12E-11 | | 0.002840641 | | 0.001899083 | |
| superpathway of L-serine and glycine biosynthesis I | 48.20188801 | | 3.85E-12 | 1.32E-11 | | 0.004790061 | | 0.005630826 | |
| superpathway of lipopolysaccharide biosynthesis | 47.70780727 | | 4.95E-12 | 1.68E-11 | | 0.000618503 | | 0.00028015 | |
| superpathway of arginine and polyamine biosynthesis | 47.68467271 | | 5.01E-12 | 1.69E-11 | | 0.002085126 | | 0.001406051 | |
| stearate biosynthesis II (bacteria and plants) | 47.03029758 | | 6.99E-12 | 2.33E-11 | | 0.00306861 | | 0.002117204 | |
| superpathway of geranylgeranyl diphosphate biosynthesis II (via MEP) | 46.6543427 | | 8.47E-12 | 2.80E-11 | | 0.003005042 | | 0.004169261 | |
| superpathway of thiamin diphosphate biosynthesis I | 46.11101826 | | 1.12E-11 | 3.66E-11 | | 0.002420593 | | 0.001656871 | |
| NAD biosynthesis II (from tryptophan) | 45.97042777 | | 1.20E-11 | 3.89E-11 | | 2.20E-05 | | 6.27E-05 | |
| **Pathways^1^** | **Test-Statistic** | | ***p*-value** | **FDR_*p*** | | **Control_mean** | | **Probiotics_mean** | |
| pentose phosphate pathway | 45.68360262 | | 1.39E-11 | 4.47E-11 | | 0.005302328 | | 0.006169425 | |
| L-tryptophan degradation to 2-amino-3-carboxymuconate semialdehyde | 45.58995981 | | 1.46E-11 | 4.64E-11 | | 1.90E-05 | | 4.70E-05 | |
| tetrapyrrole biosynthesis I (from glutamate) | 44.98521989 | | 1.99E-11 | 6.26E-11 | | 0.004110991 | | 0.003316681 | |
| pyrimidine deoxyribonucleotides de novo biosynthesis II | 44.85129268 | | 2.13E-11 | 6.65E-11 | | 0.004398704 | | 0.005519239 | |
| sulfoglycolysis | 44.65707593 | | 2.35E-11 | 7.27E-11 | | 0.000756334 | | 0.000439254 | |
| superpathway of heme biosynthesis from glycine | 44.31271115 | | 2.80E-11 | 8.59E-11 | | 0.000742172 | | 0.000407533 | |
| guanosine ribonucleotides de novo biosynthesis | 42.52863151 | | 6.97E-11 | 2.12E-10 | | 0.007028225 | | 0.008277705 | |
| superpathway of ubiquinol-8 biosynthesis (prokaryotic) | 42.05433702 | | 8.88E-11 | 2.68E-10 | | 0.001843031 | | 0.001241615 | |
| reductive TCA cycle I | 41.47120364 | | 1.20E-10 | 3.58E-10 | | 0.001339969 | | 0.000764117 | |
| superpathway of polyamine biosynthesis I | 41.31095554 | | 1.30E-10 | 3.85E-10 | | 0.00190208 | | 0.001235274 | |
| UDP-2,3-diacetamido-2,3-dideoxy-alpha-D-mannuronate biosynthesis | 41.20800977 | | 1.37E-10 | 4.02E-10 | | 6.06E-06 | | 2.50E-05 | |
| L-arginine biosynthesis III (via N-acetyl-L-citrulline) | 39.99692078 | | 2.54E-10 | 7.42E-10 | | 0.002857199 | | 0.002117473 | |
| D-galacturonate degradation I | 39.14619934 | | 3.93E-10 | 1.14E-09 | | 0.002185219 | | 0.001474966 | |
| ubiquinol-10 biosynthesis (prokaryotic) | 38.31049196 | | 6.03E-10 | 1.69E-09 | | 0.00190335 | | 0.001310365 | |
| ubiquinol-7 biosynthesis (prokaryotic) | 38.31049196 | | 6.03E-10 | 1.69E-09 | | 0.00190335 | | 0.001310365 | |
| ubiquinol-8 biosynthesis (prokaryotic) | 38.31049196 | | 6.03E-10 | 1.69E-09 | | 0.00190335 | | 0.001310365 | |
| ubiquinol-9 biosynthesis (prokaryotic) | 38.31049196 | | 6.03E-10 | 1.69E-09 | | 0.00190335 | | 0.001310365 | |
| superpathway of heme biosynthesis from glutamate | 37.62096445 | | 8.59E-10 | 2.39E-09 | | 0.002313069 | | 0.001756336 | |
| methylerythritol phosphate pathway I | 37.56415468 | | 8.85E-10 | 2.40E-09 | | 0.003129298 | | 0.004162135 | |
| methylerythritol phosphate pathway II | 37.56415468 | | 8.85E-10 | 2.40E-09 | | 0.003129298 | | 0.004162135 | |
| catechol degradation II (meta-cleavage pathway) | 37.58583029 | | 8.75E-10 | 2.40E-09 | | 1.76E-06 | | 1.48E-05 | |
| L-1,2-propanediol degradation | 37.54685225 | | 8.92E-10 | 2.40E-09 | | 0.00181895 | | 0.001167363 | |
| CMP-3-deoxy-D-manno-octulosonate biosynthesis I | 37.14652971 | | 1.10E-09 | 2.92E-09 | | 0.002286718 | | 0.001605421 | |
| sulfate reduction I (assimilatory) | 37.06783971 | | 1.14E-09 | 3.02E-09 | | 0.004078846 | | 0.003277153 | |
| superpathway of pyrimidine deoxyribonucleoside salvage | 35.66859331 | | 2.34E-09 | 6.15E-09 | | 0.004833946 | | 0.004292945 | |
| glucose degradation (oxidative) | 35.4824835 | | 2.57E-09 | 6.71E-09 | | 3.63E-06 | | 2.31E-05 | |
| L-ornithine biosynthesis | 35.2774819 | | 2.86E-09 | 7.40E-09 | | 0.003593928 | | 0.004449098 | |
| flavin biosynthesis I (bacteria and plants) | 35.21093918 | | 2.96E-09 | 7.60E-09 | | 0.00443555 | | 0.003729227 | |
| **Pathways^1^** | **Test-Statistic** | | ***p*-value** | **FDR_*p*** | | **Control_mean** | | **Probiotics_mean** | |
| L-tryptophan degradation IX | 34.63625276 | | 3.97E-09 | 1.01E-08 | | 2.99E-07 | | 6.88E-06 | |
| TCA cycle VI (obligate autotrophs) | 33.95310701 | | 5.65E-09 | 1.43E-08 | | 0.002911802 | | 0.003918288 | |
| catechol degradation to 2-oxopent-4-enoate II | 33.24347199 | | 8.13E-09 | 2.04E-08 | | 2.05E-06 | | 1.05E-05 | |
| fatty acid elongation - saturated | 33.18070014 | | 8.40E-09 | 2.10E-08 | | 0.007960526 | | 0.006901371 | |
| glutaryl-CoA degradation | 32.87806565 | | 9.81E-09 | 2.43E-08 | | 0.000484128 | | 0.000217083 | |
| lipid IVA biosynthesis | 32.29853198 | | 1.32E-08 | 3.25E-08 | | 0.002045753 | | 0.001476322 | |
| superpathway of pyrimidine ribonucleosides salvage | 32.26120842 | | 1.35E-08 | 3.29E-08 | | 0.005423565 | | 0.004785895 | |
| 2-amino-3-carboxymuconate semialdehyde degradation to 2-oxopentenoate | 32.0407514 | | 1.51E-08 | 3.66E-08 | | 2.35E-07 | | 3.10E-06 | |
| superpathway of sulfate assimilation and cysteine biosynthesis | 31.98571765 | | 1.55E-08 | 3.74E-08 | | 0.004227179 | | 0.003506764 | |
| L-tyrosine degradation I | 31.40774084 | | 2.09E-08 | 5.01E-08 | | 7.27E-05 | | 0.000243046 | |
| L-arginine biosynthesis II (acetyl cycle) | 31.2378932 | | 2.28E-08 | 5.43E-08 | | 0.004742095 | | 0.005634652 | |
| pyrimidine deoxyribonucleosides salvage | 30.75170543 | | 2.93E-08 | 6.92E-08 | | 0.005764071 | | 0.005107124 | |
| chlorophyllide a biosynthesis II (anaerobic) | 30.69070515 | | 3.03E-08 | 7.05E-08 | | 3.14E-07 | | 4.63E-06 | |
| chlorophyllide a biosynthesis III (aerobic, light independent) | 30.69070515 | | 3.03E-08 | 7.05E-08 | | 3.14E-07 | | 4.63E-06 | |
| superpathway of pyrimidine deoxyribonucleosides degradation | 30.48689111 | | 3.36E-08 | 7.78E-08 | | 0.006100182 | | 0.005595051 | |
| superpathway of purine deoxyribonucleosides degradation | 30.14490372 | | 4.01E-08 | 9.21E-08 | | 0.005771815 | | 0.005337818 | |
| D-fructuronate degradation | 29.90267528 | | 4.54E-08 | 1.04E-07 | | 0.004040344 | | 0.003257733 | |
| taxadiene biosynthesis (engineered) | 29.43670792 | | 5.78E-08 | 1.31E-07 | | 0.002081934 | | 0.00267717 | |
| phosphopantothenate biosynthesis I | 29.37975293 | | 5.95E-08 | 1.34E-07 | | 0.004504926 | | 0.003772764 | |
| ketogluconate metabolism | 29.24011349 | | 6.39E-08 | 1.43E-07 | | 0.000869813 | | 0.000517644 | |
| purine ribonucleosides degradation | 29.23089701 | | 6.42E-08 | 1.43E-07 | | 0.006664581 | | 0.006162237 | |
| L-arginine biosynthesis I (via L-ornithine) | 29.12745839 | | 6.78E-08 | 1.50E-07 | | 0.004455127 | | 0.005218751 | |
| chlorosalicylate degradation | 29.03944068 | | 7.09E-08 | 1.56E-07 | | 1.89E-06 | | 8.74E-06 | |
| superpathway of L-phenylalanine biosynthesis | 29.01865125 | | 7.17E-08 | 1.56E-07 | | 0.003185424 | | 0.002414506 | |
| L-methionine biosynthesis III | 28.40969911 | | 9.82E-08 | 2.10E-07 | | 0.002299402 | | 0.002811339 | |
| myo-inositol degradation I | 28.41059525 | | 9.81E-08 | 2.10E-07 | | 0.001099152 | | 0.00079305 | |
| **Pathways^1^** | **Test-Statistic** | | ***p*-value** | **FDR_*p*** | | **Control_mean** | | **Probiotics_mean** | |
| superpathway of UDP-N-acetylglucosamine-derived O-antigen building blocks biosynthesis | 28.4101327 | | 9.82E-08 | 2.10E-07 | | 2.61E-05 | | 6.22E-05 | |
| 6-hydroxymethyl-dihydropterin diphosphate biosynthesis III (Chlamydia) | 27.83859589 | | 1.32E-07 | 2.81E-07 | | 0.004649286 | | 0.00395645 | |
| incomplete reductive TCA cycle | 27.64957235 | | 1.45E-07 | 3.08E-07 | | 0.004000199 | | 0.003270567 | |
| queuosine biosynthesis | 27.44176218 | | 1.62E-07 | 3.38E-07 | | 0.005663357 | | 0.00498239 | |
| cis-vaccenate biosynthesis | 27.44242888 | | 1.62E-07 | 3.38E-07 | | 0.008279906 | | 0.007201487 | |
| pantothenate and coenzyme A biosynthesis I | 27.01871176 | | 2.01E-07 | 4.19E-07 | | 0.004470775 | | 0.003839122 | |
| thiazole biosynthesis II (Bacillus) | 26.81499164 | | 2.24E-07 | 4.62E-07 | | 0.002652086 | | 0.002225046 | |
| superpathway of glycolysis, pyruvate dehydrogenase, TCA, and glyoxylate bypass | 26.44107787 | | 2.72E-07 | 5.58E-07 | | 0.002660963 | | 0.001992045 | |
| tRNA processing | 26.37082461 | | 2.82E-07 | 5.75E-07 | | 0.002040931 | | 0.001560152 | |
| cob(II)yrinate a,c-diamide biosynthesis I (early cobalt insertion) | 26.04873536 | | 3.33E-07 | 6.76E-07 | | 0.000638521 | | 0.00046674 | |
| superpathway of N-acetylneuraminate degradation | 25.94709693 | | 3.51E-07 | 7.08E-07 | | 0.002447953 | | 0.001847417 | |
| 2-nitrobenzoate degradation I | 25.91782972 | | 3.56E-07 | 7.15E-07 | | 2.70E-07 | | 3.52E-06 | |
| pyrimidine deoxyribonucleotide phosphorylation | 25.55192202 | | 4.31E-07 | 8.59E-07 | | 0.005058724 | | 0.004450976 | |
| superpathway of L-tyrosine biosynthesis | 25.06985112 | | 5.53E-07 | 1.10E-06 | | 0.003101393 | | 0.00237011 | |
| nitrate reduction VI (assimilatory) | 24.89670172 | | 6.05E-07 | 1.19E-06 | | 0.000400494 | | 0.000261433 | |
| pyrimidine deoxyribonucleotides de novo biosynthesis III | 24.82043029 | | 6.29E-07 | 1.23E-06 | | 0.000434774 | | 0.000249933 | |
| ectoine biosynthesis | 24.68684693 | | 6.74E-07 | 1.31E-06 | | 1.09E-05 | | 8.65E-06 | |
| superpathway of hexuronide and hexuronate degradation | 24.33345113 | | 8.10E-07 | 1.57E-06 | | 0.001671664 | | 0.001177911 | |
| gallate degradation II | 24.00598208 | | 9.60E-07 | 1.84E-06 | | 0.000101562 | | 0.000113897 | |
| gluconeogenesis I | 24.00868199 | | 9.59E-07 | 1.84E-06 | | 0.006120158 | | 0.005495914 | |
| methylgallate degradation | 23.98114289 | | 9.73E-07 | 1.85E-06 | | 0.000121867 | | 0.000136172 | |
| photorespiration | 23.67158147 | | 1.14E-06 | 2.17E-06 | | 5.62E-06 | | 6.51E-05 | |
| L-arabinose degradation IV | 23.60019291 | | 1.19E-06 | 2.24E-06 | | 3.99E-07 | | 1.07E-05 | |
| meta cleavage pathway of aromatic compounds | 23.55030739 | | 1.22E-06 | 2.28E-06 | | 1.26E-06 | | 7.33E-06 | |
| superpathway of S-adenosyl-L-methionine biosynthesis | 23.48924817 | | 1.26E-06 | 2.34E-06 | | 0.003697365 | | 0.004662505 | |
| L-leucine degradation I | 23.07196073 | | 1.56E-06 | 2.90E-06 | | 8.83E-05 | | 0.000288137 | |
| **Pathways^1^** | **Test-Statistic** | | ***p*-value** | **FDR_*p*** | | **Control_mean** | | **Probiotics_mean** | |
| superpathway of glyoxylate bypass and TCA | 23.01745792 | | 1.61E-06 | 2.96E-06 | | 0.002409101 | | 0.001826951 | |
| glyoxylate cycle | 22.81052513 | | 1.79E-06 | 3.28E-06 | | 0.00188927 | | 0.001419601 | |
| L-arginine biosynthesis IV (archaebacteria) | 22.75703513 | | 1.84E-06 | 3.36E-06 | | 0.003839661 | | 0.004623837 | |
| sucrose degradation IV (sucrose phosphorylase) | 22.57160579 | | 2.02E-06 | 3.68E-06 | | 0.003236505 | | 0.004253335 | |
| toluene degradation IV (aerobic) (via catechol) | 22.5394037 | | 2.06E-06 | 3.72E-06 | | 1.52E-05 | | 3.49E-05 | |
| L-lysine biosynthesis II | 22.1460002 | | 2.53E-06 | 4.54E-06 | | 0.003408133 | | 0.004444275 | |
| toluene degradation III (aerobic) (via p-cresol) | 21.82804466 | | 2.98E-06 | 5.31E-06 | | 0.000209839 | | 0.000295119 | |
| glycogen biosynthesis I (from ADP-D-Glucose) | 21.8314279 | | 2.98E-06 | 5.31E-06 | | 0.002860109 | | 0.003896833 | |
| palmitate biosynthesis II (bacteria and plants) | 21.77712773 | | 3.06E-06 | 5.42E-06 | | 0.002549235 | | 0.00178382 | |
| TCA cycle IV (2-oxoglutarate decarboxylase) | 21.54255598 | | 3.46E-06 | 6.10E-06 | | 0.002708034 | | 0.002099849 | |
| L-methionine salvage cycle III | 21.50909847 | | 3.52E-06 | 6.17E-06 | | 1.04E-06 | | 3.61E-06 | |
| isopropanol biosynthesis | 21.22998158 | | 4.07E-06 | 7.10E-06 | | 3.32E-05 | | 7.53E-05 | |
| urate biosynthesis/inosine 5'-phosphate degradation | 21.06961169 | | 4.43E-06 | 7.68E-06 | | 0.003870904 | | 0.003167148 | |
| purine nucleobases degradation I (anaerobic) | 21.02312054 | | 4.54E-06 | 7.83E-06 | | 0.001211203 | | 0.000916464 | |
| L-tryptophan biosynthesis | 20.62936418 | | 5.57E-06 | 9.57E-06 | | 0.004644968 | | 0.004037222 | |
| starch degradation V | 20.32422262 | | 6.54E-06 | 1.12E-05 | | 0.003712322 | | 0.005141395 | |
| superpathway of L-threonine metabolism | 20.21107788 | | 6.94E-06 | 1.18E-05 | | 0.000453726 | | 0.000311451 | |
| purine nucleotides degradation II (aerobic) | 20.14624872 | | 7.17E-06 | 1.21E-05 | | 0.002090213 | | 0.001499059 | |
| nylon-6 oligomer degradation | 19.92645033 | | 8.05E-06 | 1.36E-05 | | 4.03E-07 | | 3.65E-06 | |
| peptidoglycan maturation (meso-diaminopimelate containing) | 19.59231777 | | 9.59E-06 | 1.61E-05 | | 0.006563418 | | 0.007543363 | |
| vanillin and vanillate degradation II | 19.37915095 | | 1.07E-05 | 1.79E-05 | | 9.36E-07 | | 5.03E-06 | |
| superpathway of vanillin and vanillate degradation | 19.35375493 | | 1.09E-05 | 1.79E-05 | | 8.54E-07 | | 4.52E-06 | |
| vanillin and vanillate degradation I | 19.35375493 | | 1.09E-05 | 1.79E-05 | | 8.54E-07 | | 4.52E-06 | |
| adenosine nucleotides degradation II | 19.23643608 | | 1.15E-05 | 1.90E-05 | | 0.002282817 | | 0.001569609 | |
| glycine betaine degradation I | 18.99101215 | | 1.31E-05 | 2.15E-05 | | 3.23E-06 | | 9.30E-06 | |
| acetylene degradation | 18.51296191 | | 1.69E-05 | 2.75E-05 | | 0.00728963 | | 0.008597502 | |
| NAD salvage pathway I | 18.4253002 | | 1.77E-05 | 2.87E-05 | | 0.002507224 | | 0.003474966 | |
| L-methionine biosynthesis I | 17.73468992 | | 2.54E-05 | 4.10E-05 | | 0.003099487 | | 0.003955744 | |
| **Pathways^1^** | **Test-Statistic** | | ***p*-value** | **FDR_*p*** | | **Control_mean** | | **Probiotics_mean** | |
| NAD biosynthesis I (from aspartate) | 17.25993678 | | 3.26E-05 | 5.24E-05 | | 0.002614555 | | 0.00318725 | |
| superpathway of L-arginine and L-ornithine degradation | 17.12423095 | | 3.50E-05 | 5.57E-05 | | 0.001065622 | | 0.00081011 | |
| superpathway of L-arginine, putrescine, and 4-aminobutanoate degradation | 17.12423095 | | 3.50E-05 | 5.57E-05 | | 0.001065622 | | 0.00081011 | |
| fatty acid salvage | 17.05099071 | | 3.64E-05 | 5.77E-05 | | 0.00016539 | | 0.000523578 | |
| pentose phosphate pathway (non-oxidative branch) | 16.86987841 | | 4.00E-05 | 6.31E-05 | | 0.008472037 | | 0.008583724 | |
| mono-trans, poly-cis decaprenyl phosphate biosynthesis | 16.77969239 | | 4.20E-05 | 6.59E-05 | | 0.000148107 | | 8.93E-05 | |
| 1,4-dihydroxy-6-naphthoate biosynthesis I | 16.04514631 | | 6.18E-05 | 9.67E-05 | | 4.71E-06 | | 6.17E-06 | |
| hexitol fermentation to lactate, formate, ethanol and acetate | 15.85682844 | | 6.83E-05 | 0.000106309 | | 0.003581049 | | 0.00306502 | |
| pyruvate fermentation to acetone | 15.76347052 | | 7.18E-05 | 0.000111187 | | 0.001162707 | | 0.000754187 | |
| TCA cycle V (2-oxoglutarate:ferredoxin oxidoreductase) | 15.73183497 | | 7.30E-05 | 0.00011256 | | 0.004651062 | | 0.004033544 | |
| superpathway of guanosine nucleotides de novo biosynthesis I | 14.75350975 | | 0.000122519 | 0.000188116 | | 0.006114804 | | 0.005586013 | |
| adenine and adenosine salvage III | 14.63347555 | | 0.000130574 | 0.000199601 | | 0.007054136 | | 0.006669327 | |
| cob(II)yrinate a,c-diamide biosynthesis II (late cobalt incorporation) | 14.26082912 | | 0.000159142 | 0.000242204 | | 2.09E-05 | | 6.04E-05 | |
| 4-methylcatechol degradation (ortho cleavage) | 14.08381644 | | 0.000174842 | 0.000264935 | | 0.000167865 | | 0.000223245 | |
| thiamin salvage II | 13.61195682 | | 0.00022475 | 0.000339079 | | 0.006112215 | | 0.005795145 | |
| tRNA charging | 13.58219803 | | 0.000228341 | 0.000343005 | | 0.00383257 | | 0.004322762 | |
| mixed acid fermentation | 13.53225403 | | 0.000234498 | 0.000350737 | | 0.004874175 | | 0.005488562 | |
| superpathway of N-acetylglucosamine, N-acetylmannosamine and N-acetylneuraminate degradation | 13.43212315 | | 0.000247352 | 0.000368374 | | 0.003055102 | | 0.00267967 | |
| glycogen degradation II (eukaryotic) | 13.23890494 | | 0.000274198 | 0.00040661 | | 1.81E-05 | | 2.06E-05 | |
| superpathway of GDP-mannose-derived O-antigen building blocks biosynthesis | 13.1238282 | | 0.000291564 | 0.000430522 | | 0.001249647 | | 0.000945171 | |
| TCA cycle VII (acetate-producers) | 13.10448459 | | 0.00029459 | 0.000433147 | | 0.004512624 | | 0.00391681 | |
| superpathway of guanosine nucleotides de novo biosynthesis II | 12.95423648 | | 0.000319198 | 0.000467349 | | 0.006044929 | | 0.005582893 | |
| 2-methylcitrate cycle I | 12.86051392 | | 0.000335588 | 0.000489282 | | 0.00073913 | | 0.000541032 | |
| colanic acid building blocks biosynthesis | 12.66705204 | | 0.000372156 | 0.000540327 | | 0.001426496 | | 0.001118744 | |
| allantoin degradation IV (anaerobic) | 12.37791896 | | 0.000434441 | 0.000628129 | | 0.001360516 | | 0.001133072 | |
| **Pathways^1^** | **Test-Statistic** | | ***p*-value** | **FDR_*p*** | | **Control_mean** | | **Probiotics_mean** | |
| superpathway of fucose and rhamnose degradation | 12.28376111 | | 0.000456917 | 0.000657884 | | 0.00107772 | | 0.000774202 | |
| fucose degradation | 12.05821374 | | 0.000515647 | 0.000739378 | | 0.001066292 | | 0.000743213 | |
| 2-methylcitrate cycle II | 12.00852082 | | 0.000529579 | 0.00075623 | | 0.000759875 | | 0.000562269 | |
| pyrimidine deoxyribonucleotides de novo biosynthesis I | 11.95413008 | | 0.000545264 | 0.000775437 | | 0.004713544 | | 0.004370002 | |
| aerobic respiration I (cytochrome c) | 11.87248783 | | 0.000569693 | 0.000806871 | | 0.001060367 | | 0.002158253 | |
| gondoate biosynthesis (anaerobic) | 11.8290264 | | 0.000583145 | 0.000822566 | | 0.008899305 | | 0.008339958 | |
| L-rhamnose degradation II | 11.42931499 | | 0.000722943 | 0.001015633 | | 2.57E-07 | | 8.66E-07 | |
| L-rhamnose degradation I | 11.38270435 | | 0.000741311 | 0.001037238 | | 0.001667707 | | 0.001437232 | |
| adenosylcobalamin salvage from cobinamide I | 11.25166516 | | 0.000795516 | 0.001108611 | | 0.001688468 | | 0.001361277 | |
| glycogen degradation I (bacterial) | 11.19548468 | | 0.000819966 | 0.001138113 | | 0.003532523 | | 0.004675903 | |
| superpathway of hexitol degradation (bacteria) | 11.1635977 | | 0.00083418 | 0.001153229 | | 0.003527179 | | 0.003209785 | |
| superpathway of salicylate degradation | 11.11607005 | | 0.000855829 | 0.001178463 | | 0.000164792 | | 0.00021585 | |
| allantoin degradation to glyoxylate III | 11.06624356 | | 0.000879136 | 0.001205772 | | 0.001238283 | | 0.001046946 | |
| octane oxidation | 10.97630993 | | 0.00092284 | 0.00126073 | | 7.93E-05 | | 9.83E-05 | |
| N10-formyl-tetrahydrofolate biosynthesis | 10.84811491 | | 0.000988964 | 0.001345766 | | 0.003338572 | | 0.003812139 | |
| mycolyl-arabinogalactan-peptidoglycan complex biosynthesis | 10.74399971 | | 0.00104618 | 0.001418064 | | 2.90E-05 | | 2.75E-05 | |
| L-histidine degradation I | 10.6158085 | | 0.001121249 | 0.001513905 | | 0.002570178 | | 0.002248367 | |
| acetyl-CoA fermentation to butanoate II | 10.25634228 | | 0.001362155 | 0.001832045 | | 0.000115451 | | 0.000229675 | |
| superpathway of phospholipid biosynthesis I (bacteria) | 10.18419305 | | 0.001416497 | 0.001897778 | | 0.007748095 | | 0.00830753 | |
| adenosylcobalamin biosynthesis II (late cobalt incorporation) | 10.07772778 | | 0.001500723 | 0.002002888 | | 3.10E-06 | | 9.02E-06 | |
| ethylmalonyl-CoA pathway | 9.861393623 | | 0.001687832 | 0.002243976 | | 4.31E-07 | | 3.23E-06 | |
| glycerol degradation to butanol | 9.743688466 | | 0.001799395 | 0.002383168 | | 0.000421116 | | 0.00025992 | |
| superpathway of menaquinol-8 biosynthesis II | 9.217677604 | | 0.002396894 | 0.003150463 | | 2.40E-05 | | 2.41E-05 | |
| 1,4-dihydroxy-6-naphthoate biosynthesis II | 9.222821909 | | 0.002390169 | 0.003150463 | | 1.08E-05 | | 1.02E-05 | |
| sucrose degradation III (sucrose invertase) | 9.18787744 | | 0.002436232 | 0.003190085 | | 0.007871261 | | 0.00830428 | |
| 4-deoxy-L-threo-hex-4-enopyranuronate degradation | 8.904917848 | | 0.002844037 | 0.003710078 | | 0.00201653 | | 0.001787706 | |
| GDP-D-glycero-alpha-D-manno-heptose biosynthesis | 8.796700839 | | 0.003017758 | 0.003921955 | | 8.64E-05 | | 5.27E-05 | |
| 4-aminobutanoate degradation V | 8.789221948 | | 0.003030155 | 0.003923372 | | 0.001508961 | | 0.001212106 | |
| **Pathways^1^** | **Test-Statistic** | | ***p*-value** | **FDR_*p*** | | **Control_mean** | | **Probiotics_mean** | |
| superpathway of L-methionine biosynthesis (transsulfuration) | 8.648778934 | | 0.003272806 | 0.004221798 | | 0.003205833 | | 0.003731029 | |
| superpathway of methylglyoxal degradation | 8.595393438 | | 0.003370144 | 0.004331259 | | 0.000758536 | | 0.000556302 | |
| 3-phenylpropanoate degradation | 8.486432241 | | 0.003578048 | 0.004581486 | | 0.000128039 | | 0.000155087 | |
| superpathway of glycol metabolism and degradation | 8.263968874 | | 0.004043962 | 0.005159025 | | 0.001068146 | | 0.000802968 | |
| superpathway of tetrahydrofolate biosynthesis and salvage | 8.074885758 | | 0.004488294 | 0.005704901 | | 0.005654472 | | 0.005891009 | |
| TCA cycle I (prokaryotic) | 7.810173258 | | 0.005195292 | 0.00657944 | | 0.005307499 | | 0.004754202 | |
| 6-hydroxymethyl-dihydropterin diphosphate biosynthesis I | 7.754778119 | | 0.005357062 | 0.006743775 | | 0.005250129 | | 0.005528752 | |
| aspartate superpathway | 7.752465924 | | 0.005363925 | 0.006743775 | | 0.002665301 | | 0.002316252 | |
| arginine, ornithine and proline interconversion | 7.545318884 | | 0.006016617 | 0.007537062 | | 0.004075642 | | 0.003663799 | |
| superpathway of glucose and xylose degradation | 7.485016421 | | 0.00622145 | 0.007765623 | | 0.003629361 | | 0.003310687 | |
| reductive acetyl coenzyme A pathway | 7.47135534 | | 0.006268834 | 0.007796722 | | 3.56E-05 | | 1.53E-05 | |
| superpathway of UDP-glucose-derived O-antigen building blocks biosynthesis | 7.064980473 | | 0.007860517 | 0.009741426 | | 0.002018061 | | 0.001724209 | |
| mycothiol biosynthesis | 6.541006485 | | 0.010541569 | 0.013017525 | | 3.21E-05 | | 3.22E-05 | |
| protocatechuate degradation II (ortho-cleavage pathway) | 6.501686829 | | 0.01077722 | 0.013261331 | | 0.000310995 | | 0.000442881 | |
| nicotinate degradation I | 6.316379944 | | 0.011962761 | 0.01466812 | | 0.000243194 | | 8.15E-05 | |
| methanol oxidation to carbon dioxide | 6.060051287 | | 0.013827367 | 0.016894705 | | 1.94E-06 | | 8.35E-06 | |
| mycolate biosynthesis | 5.6323332 | | 0.017632148 | 0.021467913 | | 0.002520714 | | 0.002003626 | |
| phosphatidylglycerol biosynthesis I (plastidic) | 5.484659472 | | 0.019184056 | 0.023114124 | | 0.007451268 | | 0.007911525 | |
| phosphatidylglycerol biosynthesis II (non-plastidic) | 5.484659472 | | 0.019184056 | 0.023114124 | | 0.007451268 | | 0.007911525 | |
| catechol degradation to beta-ketoadipate | 5.493501155 | | 0.019087283 | 0.023114124 | | 0.000180647 | | 0.000225192 | |
| creatinine degradation I | 5.256982902 | | 0.021858881 | 0.026245784 | | 3.36E-06 | | 8.09E-06 | |
| adenosylcobalamin salvage from cobinamide II | 4.998626944 | | 0.025367435 | 0.030353448 | | 0.001167098 | | 0.000986231 | |
| superpathway of L-tryptophan biosynthesis | 4.833649779 | | 0.027909488 | 0.033280386 | | 0.001365482 | | 0.001135554 | |
| Calvin-Benson-Bassham cycle | 4.7154483 | | 0.02989277 | 0.035523258 | | 0.007383906 | | 0.006956949 | |
| adenosylcobalamin biosynthesis from cobyrinate a,c-diamide I | 4.682642139 | | 0.030468851 | 0.036084271 | | 0.001146566 | | 0.000975166 | |
| superpathway of tetrahydrofolate biosynthesis | 4.660063638 | | 0.030872047 | 0.036437416 | | 0.005475446 | | 0.005658564 | |
| **Pathways^1^** | **Test-Statistic** | | ***p*-value** | **FDR_*p*** | | **Control_mean** | | **Probiotics_mean** | |
| polyisoprenoid biosynthesis (E. coli) | 4.290448023 | | 0.038327065 | 0.045000816 | | 0.006158083 | | 0.00650518 | |
| urea cycle | 4.287797475 | | 0.038386863 | 0.045000816 | | 0.001715446 | | 0.001895384 | |
| superpathway of L-methionine biosynthesis (by sulfhydrylation) | 4.279669686 | | 0.038570843 | 0.045064251 | | 0.001652293 | | 0.001840989 | |
| gallate degradation I | 4.21325417 | | 0.040109321 | 0.046704478 | | 0.000100766 | | 0.000100336 | |
| aromatic compounds degradation via beta-ketoadipate | 4.190013497 | | 0.040662774 | 0.047033275 | | 0.000198109 | | 0.000246298 | |
| catechol degradation III (ortho-cleavage pathway) | 4.190013497 | | 0.040662774 | 0.047033275 | | 0.000198109 | | 0.000246298 | |
| superpathway of L-lysine, L-threonine and L-methionine biosynthesis I | 3.75876536 | | 0.052531354 | 0.060559401 | | 0.002841353 | | 0.002530255 | |
| pyruvate fermentation to butanoate | 3.501170485 | | 0.061325472 | 0.070230821 | | 0.000195323 | | 0.000105595 | |
| superpathway of Clostridium acetobutylicum acidogenic fermentation | 3.5048919 | | 0.061187839 | 0.070230821 | | 0.000232135 | | 0.000126014 | |
| lactose and galactose degradation I | 3.475033267 | | 0.062301469 | 0.071113848 | | 0.003034731 | | 0.002862561 | |
| norspermidine biosynthesis | 3.41872073 | | 0.064460947 | 0.073337537 | | 4.03E-05 | | 2.73E-05 | |
| superpathway of geranylgeranyldiphosphate biosynthesis I (via mevalonate) | 3.07908887 | | 0.079304941 | 0.089930767 | | 0.004000118 | | 0.004492034 | |
| adenosylcobalamin biosynthesis I (early cobalt insertion) | 3.067243006 | | 0.079884845 | 0.090293293 | | 5.82E-06 | | 8.19E-06 | |
| methyl ketone biosynthesis | 2.645237074 | | 0.103860662 | 0.11701185 | | 1.51E-05 | | 2.36E-05 | |
| superpathway of pyrimidine ribonucleotides de novo biosynthesis | 2.194928619 | | 0.138465623 | 0.155493758 | | 0.006134829 | | 0.005838353 | |
| glucose and glucose-1-phosphate degradation | 2.141911117 | | 0.143323246 | 0.160429569 | | 0.002226879 | | 0.002877027 | |
| aromatic biogenic amine degradation (bacteria) | 2.102500569 | | 0.147058465 | 0.164081309 | | 0.000108472 | | 0.000109833 | |
| formaldehyde oxidation I | 2.089209245 | | 0.148342836 | 0.164983859 | | 0.002759568 | | 0.00308343 | |
| TCA cycle VIII (helicobacter) | 2.068956052 | | 0.150324388 | 0.166653555 | | 0.003669943 | | 0.003374921 | |
| L-histidine biosynthesis | 2.043390148 | | 0.152868622 | 0.168934433 | | 0.004232453 | | 0.003970117 | |
| L-glutamate degradation V (via hydroxyglutarate) | 2.012218129 | | 0.156037036 | 0.171888417 | | 3.37E-05 | | 3.76E-05 | |
| mevalonate pathway I | 1.944901043 | | 0.163137373 | 0.179141356 | | 0.003877946 | | 0.004295533 | |
| pyruvate fermentation to propanoate I | 1.682239488 | | 0.194627143 | 0.213046115 | | 0.000877164 | | 0.000799598 | |
| L-glutamate and L-glutamine biosynthesis | 1.505875288 | | 0.219769561 | 0.239811439 | | 0.000983701 | | 0.00087987 | |
| coenzyme A biosynthesis I | 1.386409047 | | 0.239012463 | 0.259991613 | | 0.006400332 | | 0.006413912 | |
| catechol degradation I (meta-cleavage pathway) | 1.359933567 | | 0.243548995 | 0.264098442 | | 0.000269852 | | 0.000212378 | |
| superpathway of L-alanine biosynthesis | 1.207873853 | | 0.271753609 | 0.293764805 | | 0.00435387 | | 0.004641397 | |
| **Pathways^1^** | **Test-Statistic** | | ***p*-value** | **FDR_*p*** | | **Control_mean** | | **Probiotics_mean** | |
| L-histidine degradation II | 1.196117871 | | 0.274098974 | 0.29537995 | | 0.000355482 | | 0.000218661 | |
| superpathway of 2,3-butanediol biosynthesis | 1.130798941 | | 0.287604988 | 0.308975018 | | 0.002108388 | | 0.001690127 | |
| phenylacetate degradation I (aerobic) | 1.029183256 | | 0.310350575 | 0.332381634 | | 0.000781671 | | 0.00073828 | |
| superpathway of purine nucleotides de novo biosynthesis I | 0.952902671 | | 0.328981546 | 0.351251066 | | 0.006086874 | | 0.005875773 | |
| superpathway of glycerol degradation to 1,3-propanediol | 0.914798685 | | 0.338844384 | 0.360671783 | | 0.000459918 | | 0.000465478 | |
| toluene degradation I (aerobic) (via o-cresol) | 0.794448466 | | 0.372758388 | 0.394351099 | | 0.000618746 | | 0.000586979 | |
| toluene degradation II (aerobic) (via 4-methylcatechol) | 0.794448466 | | 0.372758388 | 0.394351099 | | 0.000618746 | | 0.000586979 | |
| superpathway of polyamine biosynthesis II | 0.782488005 | | 0.376381298 | 0.396973588 | | 0.000130776 | | 0.000162959 | |
| superpathway of phenylethylamine degradation | 0.675057889 | | 0.411293735 | 0.432481594 | | 0.000744413 | | 0.000690395 | |
| formaldehyde assimilation II (RuMP Cycle) | 0.632127456 | | 0.426576021 | 0.44719601 | | 0.003404473 | | 0.003246769 | |
| myo-, chiro- and scillo-inositol degradation | 0.586468428 | | 0.443788014 | 0.463838677 | | 0.002158472 | | 0.002229234 | |
| peptidoglycan biosynthesis II (staphylococci) | 0.564416438 | | 0.452486245 | 0.471509691 | | 0.002114383 | | 0.002277477 | |
| glycolysis I (from glucose 6-phosphate) | 0.513488606 | | 0.473632552 | 0.492067352 | | 0.007402879 | | 0.007462166 | |
| 3-phenylpropanoate and 3-(3-hydroxyphenyl)propanoate degradation | 0.484047179 | | 0.486594807 | 0.504025069 | | 0.000618915 | | 0.000573948 | |
| 3-phenylpropanoate and 3-(3-hydroxyphenyl)propanoate degradation to 2-oxopent-4-enoate | 0.444262669 | | 0.50507219 | 0.518520857 | | 0.000543882 | | 0.000504528 | |
| superpathway of pyrimidine deoxyribonucleotides de novo biosynthesis (E. coli) | 0.449579063 | | 0.502534919 | 0.518520857 | | 0.004614437 | | 0.004669659 | |
| cinnamate and 3-hydroxycinnamate degradation to 2-oxopent-4-enoate | 0.444262669 | | 0.50507219 | 0.518520857 | | 0.000543882 | | 0.000504528 | |
| mannan degradation | 0.381879601 | | 0.536599019 | 0.549262122 | | 0.000137711 | | 0.000143709 | |
| superpathway of L-aspartate and L-asparagine biosynthesis | 0.182144555 | | 0.669536685 | 0.683321264 | | 0.002794013 | | 0.002666664 | |
| L-lysine fermentation to acetate and butanoate | 0.080255256 | | 0.776951793 | 0.790622499 | | 2.83E-05 | | 2.39E-05 | |
| GDP-mannose biosynthesis | 0.056715506 | | 0.81176462 | 0.823632524 | | 0.002633151 | | 0.002945936 | |
| superpathway of (R,R)-butanediol biosynthesis | 0.036233575 | | 0.849033944 | 0.858935214 | | 0.002086778 | | 0.001905058 | |
| isoprene biosynthesis II (engineered) | 0.027698812 | | 0.86781888 | 0.875387068 | | 1.03E-05 | | 6.38E-06 | |
| superpathway of purine nucleotides de novo biosynthesis II | 0.014041513 | | 0.905673959 | 0.910924243 | | 0.005952498 | | 0.005944523 | |
| glycolysis II (from fructose 6-phosphate) | 0.013238869 | | 0.90839734 | 0.911022765 | | 0.007517341 | | 0.007649993 | |
| homolactic fermentation | 0.000578679 | | 0.980808143 | 0.980808143 | | 0.007144742 | | 0.007279544 | |
| **Pathways^2^** | | **Pooled estimate (SMD in CLR)** | | | **95% Pooled lower limit** | | **95% Pooled upper limit** | | **Pooled *p*-value** |
| (5Z)-dodec-5-enoate biosynthesis | | -0.363014358 | | | -0.467383378 | | -0.258645338 | | 9.29E-12 |
| 1,4-dihydroxy-2-naphthoate biosynthesis I | | -0.256683745 | | | -0.360628865 | | -0.152738625 | | 1.30E-06 |
| 1,4-dihydroxy-6-naphthoate biosynthesis I | | 0.320316332 | | | 0.216134798 | | 0.424497865 | | 1.68E-09 |
| 1,4-dihydroxy-6-naphthoate biosynthesis II | | 0.260253804 | | | 0.156296788 | | 0.36421082 | | 9.26E-07 |
| 2-amino-3-carboxymuconate semialdehyde degradation to 2-oxopentenoate | | 0.445936146 | | | 0.341137352 | | 0.55073494 | | 7.43E-17 |
| 2-methylcitrate cycle I | | -0.162239074 | | | -0.265928833 | | -0.058549316 | | 0.002164544 |
| 2-methylcitrate cycle II | | -0.154312293 | | | -0.257985835 | | -0.050638751 | | 0.003530791 |
| 2-nitrobenzoate degradation I | | 0.419413217 | | | 0.314761273 | | 0.524065161 | | 4.00E-15 |
| 3-phenylpropanoate and 3-(3-hydroxyphenyl)propanoate degradation | | -0.00980196 | | | -0.113322106 | | 0.093718187 | | 0.852772481 |
| 3-phenylpropanoate and 3-(3-hydroxyphenyl)propanoate degradation to 2-oxopent-4-enoate | | -0.010903278 | | | -0.114423572 | | 0.092617016 | | 0.836452487 |
| 3-phenylpropanoate degradation | | 0.245806534 | | | 0.141896654 | | 0.349716415 | | 3.54E-06 |
| 4-aminobutanoate degradation V | | -0.111168783 | | | -0.214768271 | | -0.007569295 | | 0.035451353 |
| 4-deoxy-L-threo-hex-4-enopyranuronate degradation | | -0.098868017 | | | -0.202450793 | | 0.004714759 | | 0.061379374 |
| 4-hydroxyphenylacetate degradation | | -0.454520472 | | | -0.559368678 | | -0.349672267 | | 1.95E-17 |
| 4-methylcatechol degradation (ortho cleavage) | | 0.283337046 | | | 0.179299184 | | 0.387374909 | | 9.41E-08 |
| 5-aminoimidazole ribonucleotide biosynthesis I | | 0.270115752 | | | 0.166125029 | | 0.374106476 | | 3.56E-07 |
| 5-aminoimidazole ribonucleotide biosynthesis II | | 0.278766194 | | | 0.174744881 | | 0.382787508 | | 1.50E-07 |
| 6-hydroxymethyl-dihydropterin diphosphate biosynthesis I | | 0.075651997 | | | -0.027904567 | | 0.17920856 | | 0.152193047 |
| 6-hydroxymethyl-dihydropterin diphosphate biosynthesis III (Chlamydia) | | -0.184448669 | | | -0.288188173 | | -0.080709165 | | 0.000492482 |
| 8-amino-7-oxononanoate biosynthesis I | | -0.354063025 | | | -0.458390828 | | -0.249735222 | | 2.90E-11 |
| acetyl-CoA fermentation to butanoate II | | 0.256391873 | | | 0.152447719 | | 0.360336028 | | 1.33E-06 |
| acetylene degradation | | 0.19963833 | | | 0.09586115 | | 0.30341551 | | 0.000162971 |
| adenine and adenosine salvage III | | -0.044683037 | | | -0.148215484 | | 0.05884941 | | 0.397613673 |
| adenosine deoxyribonucleotides de novo biosynthesis II | | 0.26495623 | | | 0.160983296 | | 0.368929164 | | 5.90E-07 |
| adenosine nucleotides degradation II | | -0.174416156 | | | -0.278132403 | | -0.070699909 | | 0.000980699 |
| adenosine ribonucleotides de novo biosynthesis | | 0.282917464 | | | 0.178881132 | | 0.386953796 | | 9.82E-08 |
| **Pathways^2^** | | **Pooled estimate (SMD in CLR)** | | | **95% Pooled lower limit** | | **95% Pooled upper limit** | | **Pooled *p*-value** |
| adenosylcobalamin biosynthesis from cobyrinate a,c-diamide I | | -0.093196307 | | | -0.196772036 | | 0.010379422 | | 0.077807076 |
| adenosylcobalamin biosynthesis I (early cobalt insertion) | | 0.205039288 | | | 0.101247996 | | 0.308830579 | | 0.00010799 |
| adenosylcobalamin biosynthesis II (late cobalt incorporation) | | 0.289748458 | | | 0.185686934 | | 0.393809982 | | 4.83E-08 |
| adenosylcobalamin salvage from cobinamide I | | -0.138620597 | | | -0.242264426 | | -0.034976768 | | 0.008757083 |
| adenosylcobalamin salvage from cobinamide II | | -0.096475166 | | | -0.200054919 | | 0.007104586 | | 0.067921225 |
| ADP-L-glycero-beta-D-manno-heptose biosynthesis | | -0.40488785 | | | -0.509463104 | | -0.300312596 | | 3.24E-14 |
| aerobactin biosynthesis | | -0.331658629 | | | -0.435887713 | | -0.227429546 | | 4.47E-10 |
| aerobic respiration I (cytochrome c) | | 0.202577061 | | | 0.09879225 | | 0.306361873 | | 0.000130431 |
| allantoin degradation IV (anaerobic) | | -0.151753525 | | | -0.255422005 | | -0.048085044 | | 0.004116893 |
| allantoin degradation to glyoxylate III | | -0.13884644 | | | -0.242490674 | | -0.035202206 | | 0.008648237 |
| anhydromuropeptides recycling | | -0.354515865 | | | -0.458845729 | | -0.250186002 | | 2.74E-11 |
| arginine, ornithine and proline interconversion | | -0.074278016 | | | -0.177833247 | | 0.029277214 | | 0.159771205 |
| aromatic biogenic amine degradation (bacteria) | | 0.011173682 | | | -0.092346651 | | 0.114694015 | | 0.832455949 |
| aromatic compounds degradation via beta-ketoadipate | | 0.205215305 | | | 0.101423548 | | 0.309007063 | | 0.000106534 |
| aspartate superpathway | | -0.154238944 | | | -0.25791234 | | -0.050565548 | | 0.003546475 |
| Bifidobacterium shunt | | 1.152522517 | | | 1.04073528 | | 1.264309755 | | 8.47E-91 |
| biotin biosynthesis I | | -0.357722234 | | | -0.462066763 | | -0.253377704 | | 1.83E-11 |
| biotin biosynthesis II | | -0.362266349 | | | -0.466631887 | | -0.257900812 | | 1.02E-11 |
| Calvin-Benson-Bassham cycle | | 0.007871327 | | | -0.095648599 | | 0.111391252 | | 0.881530403 |
| catechol degradation I (meta-cleavage pathway) | | -0.001137553 | | | -0.104657087 | | 0.10238198 | | 0.982816798 |
| catechol degradation II (meta-cleavage pathway) | | 0.470031406 | | | 0.365091593 | | 0.574971219 | | 1.65E-18 |
| catechol degradation III (ortho-cleavage pathway) | | 0.205215305 | | | 0.101423548 | | 0.309007063 | | 0.000106534 |
| catechol degradation to beta-ketoadipate | | 0.216572114 | | | 0.112749436 | | 0.320394791 | | 4.34E-05 |
| catechol degradation to 2-oxopent-4-enoate II | | 0.43826798 | | | 0.333512546 | | 0.543023414 | | 2.40E-16 |
| CDP-diacylglycerol biosynthesis I | | 0.244230263 | | | 0.140325364 | | 0.348135162 | | 4.09E-06 |
| CDP-diacylglycerol biosynthesis II | | 0.244230263 | | | 0.140325364 | | 0.348135162 | | 4.09E-06 |
| chlorophyllide a biosynthesis II (anaerobic) | | 0.437037529 | | | 0.332288984 | | 0.541786074 | | 2.90E-16 |
| **Pathways^2^** | | **Pooled estimate (SMD in CLR)** | | | **95% Pooled lower limit** | | **95% Pooled upper limit** | | **Pooled *p*-value** |
| chlorophyllide a biosynthesis III (aerobic, light independent) | | 0.437037529 | | | 0.332288984 | | 0.541786074 | | 2.90E-16 |
| chlorosalicylate degradation | | 0.411572389 | | | 0.306962171 | | 0.516182606 | | 1.25E-14 |
| chorismate biosynthesis from 3-dehydroquinate | | 0.267590926 | | | 0.16360895 | | 0.371572901 | | 4.56E-07 |
| chorismate biosynthesis I | | 0.175480973 | | | 0.07176232 | | 0.279199627 | | 0.00091299 |
| cinnamate and 3-hydroxycinnamate degradation to 2-oxopent-4-enoate | | -0.010903278 | | | -0.114423572 | | 0.092617016 | | 0.836452487 |
| cis-vaccenate biosynthesis | | -0.221120896 | | | -0.324956422 | | -0.11728537 | | 3.00E-05 |
| CMP-3-deoxy-D-manno-octulosonate biosynthesis I | | -0.339499306 | | | -0.443762215 | | -0.235236398 | | 1.75E-10 |
| cob(II)yrinate a,c-diamide biosynthesis I (early cobalt insertion) | | -0.256608969 | | | -0.360553841 | | -0.152664096 | | 1.31E-06 |
| cob(II)yrinate a,c-diamide biosynthesis II (late cobalt incorporation) | | 0.295616501 | | | 0.191532862 | | 0.399700141 | | 2.60E-08 |
| coenzyme A biosynthesis I | | 0.041176738 | | | -0.062353761 | | 0.144707237 | | 0.435668749 |
| colanic acid building blocks biosynthesis | | -0.148219969 | | | -0.251881598 | | -0.04455834 | | 0.005071719 |
| creatinine degradation I | | 0.254153138 | | | 0.150216352 | | 0.358089924 | | 1.65E-06 |
| D-fructuronate degradation | | -0.126511907 | | | -0.230134979 | | -0.022888835 | | 0.016716177 |
| D-galactarate degradation I | | -0.358661458 | | | -0.463010308 | | -0.254312607 | | 1.62E-11 |
| D-galacturonate degradation I | | -0.280376758 | | | -0.384403872 | | -0.176349644 | | 1.27E-07 |
| D-glucarate degradation I | | -0.355368131 | | | -0.45970188 | | -0.251034381 | | 2.46E-11 |
| dTDP-L-rhamnose biosynthesis I | | 0.322929052 | | | 0.21873671 | | 0.427121394 | | 1.24E-09 |
| dTDP-N-acetylthomosamine biosynthesis | | -0.379454153 | | | -0.483901508 | | -0.275006798 | | 1.08E-12 |
| ectoine biosynthesis | | 0.396863103 | | | 0.292329081 | | 0.501397124 | | 9.99E-14 |
| enterobacterial common antigen biosynthesis | | -0.371539561 | | | -0.475948778 | | -0.267130344 | | 3.07E-12 |
| enterobactin biosynthesis | | -0.328646434 | | | -0.432862731 | | -0.224430138 | | 6.38E-10 |
| ethylmalonyl-CoA pathway | | 0.318363699 | | | 0.214190187 | | 0.422537211 | | 2.10E-09 |
| fatty acid beta-oxidation I | | -0.307098715 | | | -0.411226897 | | -0.202970533 | | 7.45E-09 |
| fatty acid elongation - saturated | | -0.194225908 | | | -0.297989323 | | -0.090462493 | | 0.000243797 |
| fatty acid salvage | | 0.323418285 | | | 0.21922391 | | 0.427612661 | | 1.17E-09 |
| flavin biosynthesis I (bacteria and plants) | | -0.203576561 | | | -0.307363993 | | -0.099789128 | | 0.000120837 |
| formaldehyde assimilation II (RuMP Cycle) | | 0.050482444 | | | -0.053053575 | | 0.154018463 | | 0.339251165 |
| **Pathways^2^** | | **Pooled estimate (SMD in CLR)** | | | **95% Pooled lower limit** | | **95% Pooled upper limit** | | **Pooled *p*-value** |
| formaldehyde oxidation I | | 0.10251946 | | | -0.001068073 | | 0.206106993 | | 0.052409455 |
| fucose degradation | | -0.147581401 | | | -0.251241809 | | -0.043920993 | | 0.00526426 |
| galactose degradation I (Leloir pathway) | | 0.391230828 | | | 0.286725263 | | 0.495736393 | | 2.18E-13 |
| gallate degradation I | | 0.215847001 | | | 0.112026347 | | 0.319667655 | | 4.60E-05 |
| gallate degradation II | | 0.339142294 | | | 0.234880943 | | 0.443403646 | | 1.82E-10 |
| GDP-D-glycero-alpha-D-manno-heptose biosynthesis | | 0.232528222 | | | 0.128659307 | | 0.336397137 | | 1.15E-05 |
| GDP-mannose biosynthesis | | -0.035601039 | | | -0.139128767 | | 0.067926689 | | 0.500317056 |
| gluconeogenesis I | | -0.05754074 | | | -0.161081694 | | 0.046000213 | | 0.27606156 |
| glucose and glucose-1-phosphate degradation | | -0.004133681 | | | -0.107653316 | | 0.099385954 | | 0.937618015 |
| glucose degradation (oxidative) | | 0.445336219 | | | 0.340540844 | | 0.550131595 | | 8.15E-17 |
| glutaryl-CoA degradation | | -0.281622352 | | | -0.385653975 | | -0.177590729 | | 1.12E-07 |
| glycerol degradation to butanol | | -0.115933955 | | | -0.219540442 | | -0.012327468 | | 0.028295316 |
| glycine betaine degradation I | | 0.354001079 | | | 0.249673557 | | 0.4583286 | | 2.92E-11 |
| glycogen biosynthesis I (from ADP-D-Glucose) | | 0.11106325 | | | 0.007463913 | | 0.214662586 | | 0.035625917 |
| glycogen degradation I (bacterial) | | 0.115130916 | | | 0.011525629 | | 0.218736203 | | 0.029405941 |
| glycogen degradation II (eukaryotic) | | 0.303538299 | | | 0.199424108 | | 0.40765249 | | 1.10E-08 |
| glycolysis I (from glucose 6-phosphate) | | 0.074652918 | | | -0.028902674 | | 0.178208509 | | 0.15767566 |
| glycolysis II (from fructose 6-phosphate) | | 0.081170307 | | | -0.022391856 | | 0.184732469 | | 0.124492454 |
| glycolysis III (from glucose) | | 0.236497937 | | | 0.132617011 | | 0.340378862 | | 8.12E-06 |
| glyoxylate cycle | | -0.242630852 | | | -0.34653073 | | -0.138730975 | | 4.72E-06 |
| gondoate biosynthesis (anaerobic) | | -0.113499411 | | | -0.217102286 | | -0.009896537 | | 0.031778387 |
| guanosine deoxyribonucleotides de novo biosynthesis II | | 0.26495623 | | | 0.160983296 | | 0.368929164 | | 5.90E-07 |
| guanosine nucleotides degradation III | | -0.277597883 | | | -0.381615009 | | -0.173580757 | | 1.69E-07 |
| guanosine ribonucleotides de novo biosynthesis | | 0.240918783 | | | 0.137024244 | | 0.344813321 | | 5.50E-06 |
| heme biosynthesis I (aerobic) | | -0.274940097 | | | -0.378947763 | | -0.170932432 | | 2.21E-07 |
| heme biosynthesis II (anaerobic) | | -0.234090744 | | | -0.337964362 | | -0.130217125 | | 1.00E-05 |
| heterolactic fermentation | | 1.12283432 | | | 1.011452726 | | 1.234215914 | | 6.80E-87 |
| **Pathways^2^** | | **Pooled estimate (SMD in CLR)** | | | **95% Pooled lower limit** | | **95% Pooled upper limit** | | **Pooled *p*-value** |
| hexitol fermentation to lactate, formate, ethanol and acetate | | -0.074036121 | | | -0.177591119 | | 0.029518877 | | 0.161134449 |
| homolactic fermentation | | 0.056120997 | | | -0.047418912 | | 0.159660907 | | 0.288078965 |
| incomplete reductive TCA cycle | | -0.141096873 | | | -0.24474518 | | -0.037448566 | | 0.007627993 |
| inosine-5'-phosphate biosynthesis I | | 0.288461508 | | | 0.184404775 | | 0.39251824 | | 5.53E-08 |
| inosine-5'-phosphate biosynthesis III | | 0.279837406 | | | 0.175812238 | | 0.383862574 | | 1.35E-07 |
| isoprene biosynthesis II (engineered) | | 0.095646848 | | | -0.007931875 | | 0.199225571 | | 0.070315418 |
| isopropanol biosynthesis | | 0.36496414 | | | 0.260586007 | | 0.469342273 | | 7.23E-12 |
| Kdo transfer to lipid IVA III (Chlamydia) | | -0.36496563 | | | -0.46934377 | | -0.26058749 | | 7.22E-12 |
| ketogluconate metabolism | | -0.259770411 | | | -0.363725807 | | -0.155815016 | | 9.70E-07 |
| L-1,2-propanediol degradation | | -0.333232659 | | | -0.43746847 | | -0.228996847 | | 3.71E-10 |
| lactose and galactose degradation I | | -0.053079138 | | | -0.156616897 | | 0.050458622 | | 0.315000337 |
| L-arabinose degradation IV | | 0.417621261 | | | 0.312978921 | | 0.522263601 | | 5.20E-15 |
| L-arginine biosynthesis I (via L-ornithine) | | 0.107836519 | | | 0.004241751 | | 0.211431286 | | 0.041328826 |
| L-arginine biosynthesis II (acetyl cycle) | | 0.208274842 | | | 0.104474918 | | 0.312074767 | | 8.40E-05 |
| L-arginine biosynthesis III (via N-acetyl-L-citrulline) | | -0.328612204 | | | -0.432828356 | | -0.224396052 | | 6.41E-10 |
| L-arginine biosynthesis IV (archaebacteria) | | 0.10119439 | | | -0.002391396 | | 0.204780177 | | 0.055528735 |
| L-arginine degradation II (AST pathway) | | -0.361135031 | | | -0.465495314 | | -0.256774748 | | 1.18E-11 |
| L-glutamate and L-glutamine biosynthesis | | -0.034112266 | | | -0.137639322 | | 0.069414791 | | 0.518402385 |
| L-glutamate degradation V (via hydroxyglutarate) | | 0.019734202 | | | -0.083787843 | | 0.123256247 | | 0.708684044 |
| L-histidine biosynthesis | | 0.002638246 | | | -0.100881324 | | 0.106157815 | | 0.960161741 |
| L-histidine degradation I | | -0.093395003 | | | -0.196970972 | | 0.010180966 | | 0.077176332 |
| L-histidine degradation II | | -0.005618334 | | | -0.109138063 | | 0.097901395 | | 0.915286195 |
| lipid IVA biosynthesis | | -0.328725906 | | | -0.432942538 | | -0.224509273 | | 6.32E-10 |
| L-isoleucine biosynthesis I (from threonine) | | 0.272120683 | | | 0.168122954 | | 0.376118411 | | 2.92E-07 |
| L-isoleucine biosynthesis II | | 0.242361427 | | | 0.138462392 | | 0.346260462 | | 4.83E-06 |
| L-isoleucine biosynthesis III | | 0.246466603 | | | 0.142554627 | | 0.350378579 | | 3.34E-06 |
| L-isoleucine biosynthesis IV | | -0.291558696 | | | -0.395626996 | | -0.187490397 | | 4.00E-08 |
| **Pathways^2^** | | **Pooled estimate (SMD in CLR)** | | | **95% Pooled lower limit** | | **95% Pooled upper limit** | | **Pooled *p*-value** |
| L-leucine degradation I | | 0.381568633 | | | 0.277110955 | | 0.486026311 | | 8.10E-13 |
| L-lysine biosynthesis I | | 0.162785212 | | | 0.059094306 | | 0.266476117 | | 0.002091208 |
| L-lysine biosynthesis II | | 0.250685902 | | | 0.146760401 | | 0.354611403 | | 2.27E-06 |
| L-lysine biosynthesis III | | 0.300120841 | | | 0.196019928 | | 0.404221754 | | 1.60E-08 |
| L-lysine biosynthesis VI | | 0.389320732 | | | 0.284824727 | | 0.493816737 | | 2.83E-13 |
| L-lysine fermentation to acetate and butanoate | | 0.101066863 | | | -0.002518757 | | 0.204652483 | | 0.055836945 |
| L-methionine biosynthesis I | | 0.117696186 | | | 0.014087037 | | 0.221305336 | | 0.025984252 |
| L-methionine biosynthesis III | | 0.347806166 | | | 0.243506572 | | 0.45210576 | | 6.32E-11 |
| L-methionine salvage cycle III | | 0.376916614 | | | 0.272481572 | | 0.481351655 | | 1.51E-12 |
| L-ornithine biosynthesis | | 0.089170485 | | | -0.014400495 | | 0.192741464 | | 0.091516692 |
| L-rhamnose degradation I | | -0.136133836 | | | -0.239773248 | | -0.032494425 | | 0.010039138 |
| L-rhamnose degradation II | | 0.315413254 | | | 0.21125177 | | 0.419574738 | | 2.94E-09 |
| L-tryptophan biosynthesis | | -0.113930818 | | | -0.217534327 | | -0.010327309 | | 0.031135605 |
| L-tryptophan degradation IX | | 0.459140422 | | | 0.354265244 | | 0.5640156 | | 9.43E-18 |
| L-tryptophan degradation to 2-amino-3-carboxymuconate semialdehyde | | 0.465111169 | | | 0.360200739 | | 0.570021599 | | 3.65E-18 |
| L-tyrosine degradation I | | 0.406213778 | | | 0.301631634 | | 0.510795923 | | 2.68E-14 |
| L-valine biosynthesis | | 0.272120683 | | | 0.168122954 | | 0.376118411 | | 2.92E-07 |
| mannan degradation | | 0.143563833 | | | 0.039910986 | | 0.24721668 | | 0.006634828 |
| meta cleavage pathway of aromatic compounds | | 0.410128663 | | | 0.305526045 | | 0.514731282 | | 1.53E-14 |
| methanol oxidation to carbon dioxide | | 0.266230232 | | | 0.162252937 | | 0.370207526 | | 5.21E-07 |
| methyl ketone biosynthesis | | 0.044348316 | | | -0.059183939 | | 0.14788057 | | 0.401157507 |
| methylerythritol phosphate pathway I | | 0.256613535 | | | 0.152668648 | | 0.360558423 | | 1.31E-06 |
| methylerythritol phosphate pathway II | | 0.256613535 | | | 0.152668648 | | 0.360558423 | | 1.31E-06 |
| methylgallate degradation | | 0.339259745 | | | 0.234997882 | | 0.443521609 | | 1.80E-10 |
| methylphosphonate degradation I | | -0.367735607 | | | -0.472126774 | | -0.263344439 | | 5.04E-12 |
| mevalonate pathway I | | 0.108954741 | | | 0.005358405 | | 0.212551077 | | 0.039270524 |
| mixed acid fermentation | | 0.201794111 | | | 0.098011344 | | 0.305576879 | | 0.000138443 |
| **Pathways^2^** | | **Pooled estimate (SMD in CLR)** | | | **95% Pooled lower limit** | | **95% Pooled upper limit** | | **Pooled *p*-value** |
| mono-trans, poly-cis decaprenyl phosphate biosynthesis | | -0.149705794 | | | -0.253370285 | | -0.046041304 | | 0.004648144 |
| mycolate biosynthesis | | -0.047915276 | | | -0.15144966 | | 0.055619109 | | 0.364373456 |
| mycolyl-arabinogalactan-peptidoglycan complex biosynthesis | | -0.068540136 | | | -0.172090064 | | 0.035009792 | | 0.194525072 |
| mycothiol biosynthesis | | -0.039206736 | | | -0.14273621 | | 0.064322738 | | 0.457941507 |
| myo-, chiro- and scillo-inositol degradation | | -0.011691126 | | | -0.115211535 | | 0.091829283 | | 0.824820349 |
| myo-inositol degradation I | | -0.257823301 | | | -0.3617722 | | -0.153874401 | | 1.17E-06 |
| N10-formyl-tetrahydrofolate biosynthesis | | 0.089518378 | | | -0.014053004 | | 0.19308976 | | 0.090260006 |
| NAD biosynthesis I (from aspartate) | | 0.213344148 | | | 0.109530428 | | 0.317157869 | | 5.63E-05 |
| NAD biosynthesis II (from tryptophan) | | 0.467045217 | | | 0.362123273 | | 0.571967161 | | 2.67E-18 |
| NAD salvage pathway I | | 0.128891363 | | | 0.025264362 | | 0.232518365 | | 0.014776739 |
| NAD salvage pathway II | | -0.358165863 | | | -0.462512433 | | -0.253819294 | | 1.73E-11 |
| nicotinate degradation I | | -0.043628424 | | | -0.147160269 | | 0.05990342 | | 0.408843341 |
| nitrate reduction VI (assimilatory) | | -0.246574759 | | | -0.350487079 | | -0.142662439 | | 3.31E-06 |
| norspermidine biosynthesis | | 0.202682472 | | | 0.098897384 | | 0.30646756 | | 0.000129386 |
| nylon-6 oligomer degradation | | 0.391566373 | | | 0.287059124 | | 0.496073622 | | 2.08E-13 |
| O-antigen building blocks biosynthesis (E. coli) | | 0.356443623 | | | 0.252104957 | | 0.460782289 | | 2.15E-11 |
| octane oxidation | | 0.27285436 | | | 0.168854055 | | 0.376854664 | | 2.72E-07 |
| oleate biosynthesis IV (anaerobic) | | -0.351012177 | | | -0.455326163 | | -0.24669819 | | 4.25E-11 |
| palmitate biosynthesis II (bacteria and plants) | | -0.148904447 | | | -0.25256739 | | -0.045241503 | | 0.004872431 |
| palmitoleate biosynthesis I (from (5Z)-dodec-5-enoate) | | -0.352425782 | | | -0.456746156 | | -0.248105409 | | 3.56E-11 |
| pantothenate and coenzyme A biosynthesis I | | -0.167407475 | | | -0.271108243 | | -0.063706707 | | 0.001555993 |
| pentose phosphate pathway | | 0.270998127 | | | 0.167004327 | | 0.374991928 | | 3.26E-07 |
| pentose phosphate pathway (non-oxidative branch) | | 0.085658697 | | | -0.01790831 | | 0.189225704 | | 0.105005541 |
| peptidoglycan biosynthesis I (meso-diaminopimelate containing) | | 0.280874637 | | | 0.176845723 | | 0.384903551 | | 1.21E-07 |
| peptidoglycan biosynthesis II (staphylococci) | | 0.0671842 | | | -0.036364536 | | 0.170732937 | | 0.203494544 |
| peptidoglycan biosynthesis III (mycobacteria) | | 0.368301298 | | | 0.263907458 | | 0.472695139 | | 4.69E-12 |
| peptidoglycan biosynthesis IV (Enterococcus faecium) | | 0.503097635 | | | 0.397952562 | | 0.608242708 | | 6.72E-21 |
| **Pathways^2^** | | **Pooled estimate (SMD in CLR)** | | | **95% Pooled lower limit** | | **95% Pooled upper limit** | | **Pooled *p*-value** |
| peptidoglycan biosynthesis V (beta-lactam resistance) | | 1.073977843 | | | 0.963243809 | | 1.184711877 | | 1.43E-80 |
| peptidoglycan maturation (meso-diaminopimelate containing) | | 0.25696646 | | | 0.153020404 | | 0.360912516 | | 1.26E-06 |
| phenylacetate degradation I (aerobic) | | -0.02726705 | | | -0.130791387 | | 0.076257287 | | 0.60569333 |
| phosphatidylglycerol biosynthesis I (plastidic) | | 0.135233731 | | | 0.031595898 | | 0.238871563 | | 0.010542906 |
| phosphatidylglycerol biosynthesis II (non-plastidic) | | 0.135233731 | | | 0.031595898 | | 0.238871563 | | 0.010542906 |
| phosphopantothenate biosynthesis I | | -0.205792966 | | | -0.309586256 | | -0.101999675 | | 0.000101885 |
| photorespiration | | 0.405844602 | | | 0.301264379 | | 0.510424826 | | 2.83E-14 |
| polyisoprenoid biosynthesis (E. coli) | | 0.100969503 | | | -0.00261599 | | 0.204554995 | | 0.056073207 |
| polymyxin resistance | | -0.368573751 | | | -0.47296888 | | -0.264178622 | | 4.52E-12 |
| ppGpp biosynthesis | | -0.427940154 | | | -0.53263835 | | -0.323241959 | | 1.14E-15 |
| preQ0 biosynthesis | | -0.251802628 | | | -0.355731747 | | -0.147873509 | | 2.05E-06 |
| protocatechuate degradation I (meta-cleavage pathway) | | 0.478009275 | | | 0.37302118 | | 0.582997371 | | 4.51E-19 |
| protocatechuate degradation II (ortho-cleavage pathway) | | 0.21384914 | | | 0.110034027 | | 0.317664253 | | 5.41E-05 |
| purine nucleobases degradation I (anaerobic) | | -0.181448651 | | | -0.285181065 | | -0.077716238 | | 0.000607223 |
| purine nucleotides degradation II (aerobic) | | -0.171129647 | | | -0.274838556 | | -0.067420737 | | 0.001220145 |
| purine ribonucleosides degradation | | -0.108662614 | | | -0.212258538 | | -0.005066689 | | 0.03979965 |
| pyridoxal 5'-phosphate biosynthesis I | | -0.399021926 | | | -0.50356696 | | -0.294476892 | | 7.39E-14 |
| pyrimidine deoxyribonucleosides salvage | | -0.108063747 | | | -0.211658832 | | -0.004468662 | | 0.040903319 |
| pyrimidine deoxyribonucleotide phosphorylation | | -0.114189768 | | | -0.217793659 | | -0.010585877 | | 0.030755169 |
| pyrimidine deoxyribonucleotides de novo biosynthesis I | | 0.040479587 | | | -0.063050543 | | 0.144009718 | | 0.443478313 |
| pyrimidine deoxyribonucleotides de novo biosynthesis II | | 0.400828826 | | | 0.29627453 | | 0.505383123 | | 5.74E-14 |
| pyrimidine deoxyribonucleotides de novo biosynthesis III | | -0.21394789 | | | -0.317763276 | | -0.110132505 | | 5.36E-05 |
| pyruvate fermentation to acetate and lactate II | | 0.212981493 | | | 0.10916877 | | 0.316794216 | | 5.79E-05 |
| pyruvate fermentation to acetone | | -0.183206874 | | | -0.286943429 | | -0.079470319 | | 0.000537274 |
| pyruvate fermentation to butanoate | | -0.04411364 | | | -0.14764576 | | 0.05941848 | | 0.403653401 |
| pyruvate fermentation to isobutanol (engineered) | | 0.25450624 | | | 0.150568295 | | 0.358444184 | | 1.59E-06 |
| pyruvate fermentation to propanoate I | | -0.025698039 | | | -0.129221838 | | 0.07782576 | | 0.626592832 |
| **Pathways^2^** | | **Pooled estimate (SMD in CLR)** | | | **95% Pooled lower limit** | | **95% Pooled upper limit** | | **Pooled *p*-value** |
| queuosine biosynthesis | | -0.138341395 | | | -0.241984724 | | -0.034698065 | | 0.008893337 |
| reductive acetyl coenzyme A pathway | | -0.064961157 | | | -0.168507993 | | 0.038585679 | | 0.218845705 |
| reductive TCA cycle I | | -0.318184241 | | | -0.422357019 | | -0.214011464 | | 2.14E-09 |
| S-adenosyl-L-methionine cycle I | | 0.28196329 | | | 0.177930429 | | 0.38599615 | | 1.08E-07 |
| S-methyl-5-thio-alpha-D-ribose 1-phosphate degradation | | 0.556392672 | | | 0.450888372 | | 0.661896971 | | 4.84E-25 |
| starch degradation V | | 0.125183766 | | | 0.021562856 | | 0.228804676 | | 0.017893234 |
| stearate biosynthesis II (bacteria and plants) | | -0.341911019 | | | -0.446184488 | | -0.237637549 | | 1.30E-10 |
| sucrose degradation III (sucrose invertase) | | 0.081352835 | | | -0.02220952 | | 0.184915189 | | 0.123648407 |
| sucrose degradation IV (sucrose phosphorylase) | | 0.135894582 | | | 0.032255591 | | 0.239533573 | | 0.010170906 |
| sulfate reduction I (assimilatory) | | -0.224700261 | | | -0.328546084 | | -0.120854437 | | 2.23E-05 |
| sulfoglycolysis | | -0.342063237 | | | -0.446337375 | | -0.237789099 | | 1.28E-10 |
| superpathway of heme biosynthesis from glutamate | | -0.14495511 | | | -0.248610552 | | -0.041299668 | | 0.006127548 |
| superpathway of beta-D-glucuronide and D-glucuronate degradation | | -0.31549348 | | | -0.419655289 | | -0.21133167 | | 2.91E-09 |
| superpathway of (Kdo)2-lipid A biosynthesis | | -0.393131223 | | | -0.497646344 | | -0.288616101 | | 1.68E-13 |
| superpathway of (R,R)-butanediol biosynthesis | | 0.058061922 | | | -0.045479421 | | 0.161603266 | | 0.271737146 |
| superpathway of 2,3-butanediol biosynthesis | | 0.048462767 | | | -0.055071959 | | 0.151997493 | | 0.358920318 |
| superpathway of 5-aminoimidazole ribonucleotide biosynthesis | | 0.278766194 | | | 0.174744881 | | 0.382787508 | | 1.50E-07 |
| superpathway of adenosine nucleotides de novo biosynthesis I | | 0.276489998 | | | 0.172476826 | | 0.38050317 | | 1.89E-07 |
| superpathway of adenosine nucleotides de novo biosynthesis II | | 0.276048165 | | | 0.172036566 | | 0.380059764 | | 1.97E-07 |
| superpathway of arginine and polyamine biosynthesis | | -0.267023147 | | | -0.371003167 | | -0.163043128 | | 4.82E-07 |
| superpathway of aromatic amino acid biosynthesis | | 0.179254048 | | | 0.075526748 | | 0.282981348 | | 0.000706437 |
| superpathway of branched amino acid biosynthesis | | 0.255178604 | | | 0.151238451 | | 0.359118758 | | 1.50E-06 |
| superpathway of chorismate metabolism | | -0.341143499 | | | -0.4454136 | | -0.236873399 | | 1.43E-10 |
| superpathway of Clostridium acetobutylicum acidogenic fermentation | | -0.043162816 | | | -0.146694399 | | 0.060368767 | | 0.413860752 |
| superpathway of demethylmenaquinol-6 biosynthesis I | | -0.351825877 | | | -0.456143537 | | -0.247508217 | | 3.84E-11 |
| superpathway of demethylmenaquinol-8 biosynthesis | | -0.36434111 | | | -0.468716325 | | -0.259965894 | | 7.83E-12 |
| superpathway of demethylmenaquinol-9 biosynthesis | | -0.351825877 | | | -0.456143537 | | -0.247508217 | | 3.84E-11 |
| **Pathways^2^** | | **Pooled estimate (SMD in CLR)** | | | **95% Pooled lower limit** | | **95% Pooled upper limit** | | **Pooled *p*-value** |
| superpathway of D-glucarate and D-galactarate degradation | | -0.358661458 | | | -0.463010308 | | -0.254312607 | | 1.62E-11 |
| superpathway of fatty acid biosynthesis initiation (E. coli) | | -0.354163618 | | | -0.458491879 | | -0.249835358 | | 2.86E-11 |
| superpathway of fucose and rhamnose degradation | | -0.156162821 | | | -0.259840077 | | -0.052485566 | | 0.00315543 |
| superpathway of GDP-mannose-derived O-antigen building blocks biosynthesis | | -0.161235343 | | | -0.264923004 | | -0.057547683 | | 0.002305513 |
| superpathway of geranylgeranyl diphosphate biosynthesis II (via MEP) | | 0.289974802 | | | 0.185912433 | | 0.394037171 | | 4.72E-08 |
| superpathway of geranylgeranyldiphosphate biosynthesis I (via mevalonate) | | 0.121509075 | | | 0.017894027 | | 0.225124123 | | 0.021536518 |
| superpathway of glucose and xylose degradation | | -0.078385651 | | | -0.181944939 | | 0.025173637 | | 0.137934312 |
| superpathway of glycerol degradation to 1,3-propanediol | | 0.106778447 | | | 0.003185148 | | 0.210371746 | | 0.043359915 |
| superpathway of glycol metabolism and degradation | | -0.122155375 | | | -0.225771441 | | -0.018539309 | | 0.02085237 |
| superpathway of glycolysis and Entner-Doudoroff | | -0.251264422 | | | -0.355191796 | | -0.147337049 | | 2.15E-06 |
| superpathway of glycolysis, pyruvate dehydrogenase, TCA, and glyoxylate bypass | | -0.23557433 | | | -0.339452443 | | -0.131696217 | | 8.80E-06 |
| superpathway of glyoxylate bypass and TCA | | -0.235244819 | | | -0.339121932 | | -0.131367707 | | 9.05E-06 |
| superpathway of guanosine nucleotides de novo biosynthesis I | | -0.046888764 | | | -0.150422519 | | 0.05664499 | | 0.374736573 |
| superpathway of guanosine nucleotides de novo biosynthesis II | | -0.023698647 | | | -0.127221807 | | 0.079824513 | | 0.653664443 |
| superpathway of heme biosynthesis from glycine | | -0.360237374 | | | -0.464593499 | | -0.255881249 | | 1.33E-11 |
| superpathway of heme biosynthesis from uroporphyrinogen-III | | -0.427486168 | | | -0.532181879 | | -0.322790458 | | 1.22E-15 |
| superpathway of hexitol degradation (bacteria) | | -0.053852459 | | | -0.157390753 | | 0.049685836 | | 0.308004204 |
| superpathway of hexuronide and hexuronate degradation | | -0.228570109 | | | -0.33242725 | | -0.124712968 | | 1.61E-05 |
| superpathway of histidine, purine, and pyrimidine biosynthesis | | -0.383021436 | | | -0.487486239 | | -0.278556633 | | 6.66E-13 |
| superpathway of L-alanine biosynthesis | | -0.057640853 | | | -0.161181881 | | 0.045900175 | | 0.275227266 |
| superpathway of L-arginine and L-ornithine degradation | | -0.211390046 | | | -0.31519841 | | -0.107581683 | | 6.57E-05 |
| superpathway of L-arginine, putrescine, and 4-aminobutanoate degradation | | -0.211390046 | | | -0.31519841 | | -0.107581683 | | 6.57E-05 |
| superpathway of L-aspartate and L-asparagine biosynthesis | | 0.08522777 | | | -0.018338761 | | 0.1887943 | | 0.106764345 |
| superpathway of lipopolysaccharide biosynthesis | | -0.33810681 | | | -0.442363654 | | -0.233849965 | | 2.07E-10 |
| superpathway of L-isoleucine biosynthesis I | | 0.277438412 | | | 0.173421856 | | 0.381454968 | | 1.72E-07 |
| superpathway of L-lysine, L-threonine and L-methionine biosynthesis I | | -0.164662653 | | | -0.268357532 | | -0.060967775 | | 0.001856157 |
| superpathway of L-methionine biosynthesis (by sulfhydrylation) | | 0.19757647 | | | 0.093804578 | | 0.301348362 | | 0.000190212 |
| **Pathways^2^** | | **Pooled estimate (SMD in CLR)** | | | **95% Pooled lower limit** | | **95% Pooled upper limit** | | **Pooled *p*-value** |
| superpathway of L-methionine biosynthesis (transsulfuration) | | 0.131761851 | | | 0.028130012 | | 0.23539369 | | 0.012703315 |
| superpathway of L-phenylalanine biosynthesis | | -0.28618827 | | | -0.39023659 | | -0.18213995 | | 7.01E-08 |
| superpathway of L-serine and glycine biosynthesis I | | 0.221509453 | | | 0.117672817 | | 0.325346089 | | 2.90E-05 |
| superpathway of L-threonine biosynthesis | | 0.21239003 | | | 0.108578931 | | 0.316201129 | | 6.07E-05 |
| superpathway of L-threonine metabolism | | -0.193199452 | | | -0.296960299 | | -0.089438605 | | 0.000262862 |
| superpathway of L-tryptophan biosynthesis | | -0.059180914 | | | -0.162723107 | | 0.044361278 | | 0.262610043 |
| superpathway of L-tyrosine biosynthesis | | -0.282027686 | | | -0.38606078 | | -0.177994592 | | 1.08E-07 |
| superpathway of menaquinol-10 biosynthesis | | -0.3564022 | | | -0.460740676 | | -0.252063724 | | 2.16E-11 |
| superpathway of menaquinol-11 biosynthesis | | -0.21463869 | | | -0.318455987 | | -0.110821394 | | 5.07E-05 |
| superpathway of menaquinol-12 biosynthesis | | -0.21463869 | | | -0.318455987 | | -0.110821394 | | 5.07E-05 |
| superpathway of menaquinol-13 biosynthesis | | -0.21463869 | | | -0.318455987 | | -0.110821394 | | 5.07E-05 |
| superpathway of menaquinol-6 biosynthesis I | | -0.3564022 | | | -0.460740676 | | -0.252063724 | | 2.16E-11 |
| superpathway of menaquinol-7 biosynthesis | | -0.214123978 | | | -0.31793985 | | -0.110308106 | | 5.29E-05 |
| superpathway of menaquinol-8 biosynthesis I | | -0.374850217 | | | -0.479275291 | | -0.270425143 | | 1.98E-12 |
| superpathway of menaquinol-8 biosynthesis II | | 0.260176464 | | | 0.156219708 | | 0.364133221 | | 9.33E-07 |
| superpathway of menaquinol-9 biosynthesis | | -0.3564022 | | | -0.460740676 | | -0.252063724 | | 2.16E-11 |
| superpathway of methylglyoxal degradation | | -0.11739845 | | | -0.221007147 | | -0.013789754 | | 0.026362849 |
| superpathway of N-acetylglucosamine, N-acetylmannosamine and N-acetylneuraminate degradation | | -0.12315255 | | | -0.226770198 | | -0.019534902 | | 0.019833997 |
| superpathway of N-acetylneuraminate degradation | | -0.209227439 | | | -0.313029931 | | -0.105424948 | | 7.80E-05 |
| superpathway of ornithine degradation | | -0.347855283 | | | -0.452155097 | | -0.24355547 | | 6.29E-11 |
| superpathway of phenylethylamine degradation | | -0.013461919 | | | -0.116982616 | | 0.090058779 | | 0.798819362 |
| superpathway of phospholipid biosynthesis I (bacteria) | | 0.158363557 | | | 0.054681828 | | 0.262045286 | | 0.002756614 |
| superpathway of phylloquinol biosynthesis | | -0.224176585 | | | -0.328020892 | | -0.120332279 | | 2.33E-05 |
| superpathway of polyamine biosynthesis I | | -0.260352166 | | | -0.364309512 | | -0.15639482 | | 9.17E-07 |
| superpathway of polyamine biosynthesis II | | 0.117591439 | | | 0.013982449 | | 0.221200429 | | 0.026116907 |
| superpathway of purine deoxyribonucleosides degradation | | -0.066580173 | | | -0.170128387 | | 0.036968041 | | 0.20758579 |
| **Pathways^2^** | | **Pooled estimate (SMD in CLR)** | | | **95% Pooled lower limit** | | **95% Pooled upper limit** | | **Pooled *p*-value** |
| superpathway of purine nucleotides de novo biosynthesis I | | 0.03781746 | | | -0.065711321 | | 0.141346242 | | 0.474025626 |
| superpathway of purine nucleotides de novo biosynthesis II | | 0.133187661 | | | 0.029553379 | | 0.236821942 | | 0.011772652 |
| superpathway of pyridoxal 5'-phosphate biosynthesis and salvage | | -0.397946599 | | | -0.50248614 | | -0.293407058 | | 8.59E-14 |
| superpathway of pyrimidine deoxyribonucleoside salvage | | -0.094033684 | | | -0.197610427 | | 0.00954306 | | 0.075177092 |
| superpathway of pyrimidine deoxyribonucleosides degradation | | -0.126312462 | | | -0.229935208 | | -0.022689717 | | 0.016888473 |
| superpathway of pyrimidine deoxyribonucleotides de novo biosynthesis | | -0.418225355 | | | -0.522870928 | | -0.313579782 | | 4.76E-15 |
| superpathway of pyrimidine deoxyribonucleotides de novo biosynthesis (E. coli) | | 0.181283934 | | | 0.077551907 | | 0.285015962 | | 0.000614194 |
| superpathway of pyrimidine nucleobases salvage | | 0.258200051 | | | 0.154249899 | | 0.362150204 | | 1.13E-06 |
| superpathway of pyrimidine ribonucleosides salvage | | -0.100067144 | | | -0.203651463 | | 0.003517176 | | 0.058302839 |
| superpathway of pyrimidine ribonucleotides de novo biosynthesis | | -0.001224149 | | | -0.104743684 | | 0.102295385 | | 0.981508958 |
| superpathway of S-adenosyl-L-methionine biosynthesis | | 0.133895678 | | | 0.030260174 | | 0.237531182 | | 0.011333409 |
| superpathway of salicylate degradation | | 0.265758109 | | | 0.161782433 | | 0.369733785 | | 5.45E-07 |
| superpathway of sulfate assimilation and cysteine biosynthesis | | -0.190261894 | | | -0.294015467 | | -0.086508322 | | 0.000325446 |
| superpathway of tetrahydrofolate biosynthesis | | 0.082966436 | | | -0.020597634 | | 0.186530506 | | 0.116379989 |
| superpathway of tetrahydrofolate biosynthesis and salvage | | 0.119209424 | | | 0.015597956 | | 0.222820892 | | 0.024131925 |
| superpathway of thiamin diphosphate biosynthesis I | | -0.3202238 | | | -0.424404952 | | -0.216042648 | | 1.70E-09 |
| superpathway of thiamin diphosphate biosynthesis II | | -0.400195335 | | | -0.504746379 | | -0.29564429 | | 6.27E-14 |
| superpathway of ubiquinol-8 biosynthesis (prokaryotic) | | -0.360200199 | | | -0.464556152 | | -0.255844245 | | 1.33E-11 |
| superpathway of UDP-glucose-derived O-antigen building blocks biosynthesis | | -0.055412814 | | | -0.158952212 | | 0.048126584 | | 0.294203494 |
| superpathway of UDP-N-acetylglucosamine-derived O-antigen building blocks biosynthesis | | 0.390877712 | | | 0.286373918 | | 0.495381506 | | 2.29E-13 |
| superpathway of vanillin and vanillate degradation | | 0.36791515 | | | 0.263523135 | | 0.472307166 | | 4.93E-12 |
| taxadiene biosynthesis (engineered) | | 0.283545311 | | | 0.179506688 | | 0.387583934 | | 9.21E-08 |
| TCA cycle I (prokaryotic) | | -0.064363842 | | | -0.167910178 | | 0.039182494 | | 0.22310885 |
| TCA cycle IV (2-oxoglutarate decarboxylase) | | -0.242215544 | | | -0.346114123 | | -0.138316965 | | 4.90E-06 |
| TCA cycle V (2-oxoglutarate:ferredoxin oxidoreductase) | | -0.11175776 | | | -0.215358097 | | -0.008157423 | | 0.034490482 |
| TCA cycle VI (obligate autotrophs) | | 0.133665728 | | | 0.030030622 | | 0.237300835 | | 0.011474442 |
| **Pathways^2^** | | **Pooled estimate (SMD in CLR)** | | | **95% Pooled lower limit** | | **95% Pooled upper limit** | | **Pooled *p*-value** |
| TCA cycle VII (acetate-producers) | | -0.0843324 | | | -0.187897949 | | 0.019233149 | | 0.110493426 |
| TCA cycle VIII (helicobacter) | | 0.008592572 | | | -0.094927431 | | 0.112112575 | | 0.87076661 |
| tetrapyrrole biosynthesis I (from glutamate) | | -0.259784434 | | | -0.363739876 | | -0.155828991 | | 9.68E-07 |
| tetrapyrrole biosynthesis II (from glycine) | | -0.297609499 | | | -0.401700749 | | -0.193518248 | | 2.10E-08 |
| thiamin salvage II | | -0.073792644 | | | -0.177347409 | | 0.029762121 | | 0.162515471 |
| thiazole biosynthesis I (E. coli) | | -0.298863687 | | | -0.402959753 | | -0.19476762 | | 1.83E-08 |
| thiazole biosynthesis II (Bacillus) | | -0.020546134 | | | -0.124068391 | | 0.082976124 | | 0.69727955 |
| toluene degradation I (aerobic) (via o-cresol) | | 0.00226189 | | | -0.101257668 | | 0.105781448 | | 0.965841053 |
| toluene degradation II (aerobic) (via 4-methylcatechol) | | 0.00226189 | | | -0.101257668 | | 0.105781448 | | 0.965841053 |
| toluene degradation III (aerobic) (via p-cresol) | | 0.319299358 | | | 0.215122009 | | 0.423476708 | | 1.89E-09 |
| toluene degradation IV (aerobic) (via catechol) | | 0.375241361 | | | 0.270814405 | | 0.479668318 | | 1.88E-12 |
| tRNA charging | | 0.133999887 | | | 0.030364203 | | 0.237635572 | | 0.011270004 |
| tRNA processing | | -0.284489848 | | | -0.388531927 | | -0.18044777 | | 8.36E-08 |
| ubiquinol-10 biosynthesis (prokaryotic) | | -0.353003384 | | | -0.457326375 | | -0.248680394 | | 3.31E-11 |
| ubiquinol-7 biosynthesis (prokaryotic) | | -0.353003384 | | | -0.457326375 | | -0.248680394 | | 3.31E-11 |
| ubiquinol-8 biosynthesis (prokaryotic) | | -0.353003384 | | | -0.457326375 | | -0.248680394 | | 3.31E-11 |
| ubiquinol-9 biosynthesis (prokaryotic) | | -0.353003384 | | | -0.457326375 | | -0.248680394 | | 3.31E-11 |
| UDP-2,3-diacetamido-2,3-dideoxy-alpha-D-mannuronate biosynthesis | | 0.451151491 | | | 0.346322787 | | 0.555980195 | | 3.31E-17 |
| UDP-N-acetyl-D-glucosamine biosynthesis I | | 0.280605655 | | | 0.176577714 | | 0.384633596 | | 1.24E-07 |
| UDP-N-acetylmuramoyl-pentapeptide biosynthesis I (meso-diaminopimelate containing) | | 0.279024012 | | | 0.175001773 | | 0.383046252 | | 1.46E-07 |
| UDP-N-acetylmuramoyl-pentapeptide biosynthesis II (lysine-containing) | | 0.275937993 | | | 0.171926786 | | 0.3799492 | | 2.00E-07 |
| UMP biosynthesis | | 0.265506122 | | | 0.161531309 | | 0.369480936 | | 5.59E-07 |
| urate biosynthesis/inosine 5'-phosphate degradation | | -0.0359463 | | | -0.139474188 | | 0.067581588 | | 0.49617126 |
| urea cycle | | 0.168015875 | | | 0.064313789 | | 0.271717962 | | 0.00149583 |
| vanillin and vanillate degradation I | | 0.36791515 | | | 0.263523135 | | 0.472307166 | | 4.93E-12 |
| vanillin and vanillate degradation II | | 0.368371785 | | | 0.263977611 | | 0.472765959 | | 4.64E-12 |

CLR, centered log-ratio; FDR, false discovery rate; SMD, standardised mean difference.

**Table Footnotes**

^1^ Kruskal-Wallis test results for 347 common differential metabolic pathways included in the meta-analysis.

^2^ Meta-analysis of all three included studies for 347 common differential metabolic pathways.

**Supplemental Figure 1.** Timeline of sample collection among the included studies exploring the association between probiotics and gut microbiota in preterm infants. The filled diamonds (◊) represent the start and end of probiotics supplementation for preterm infants. The filled circles (○) represent the near times when the samples were collected (timeline not to scale). Studies involving single-strain probiotics are denoted in blue, multiple-strain probiotics are denoted in orange, and single- or multiple-strain probiotics are denoted in red. *B.*, *Bifidobacterium*; *L. acidophilus*, *Lactobacillus acidophilus*; LOHS, length of hospital stay; *L. reuteri*, *Limosilactobacillus reuteri*; *L. rhamnosus*, *Lacticaseibacillus rhamnosus*; LGG, *Lacticaseibacillus rhamnosus* GG; PMW, post-menstrual week.

**Supplemental Figure 2.** Probiotic exposures and gut microbial diversity in preterm infants. (A) LOESS fits (95% CIs shaded in grey) over time for Simpson index. (B) Forest plots of the association between probiotics and Simpson index. (C) Subgroup analysis of Simpson index between probiotics and control preterm infants by probiotic types. (D) Sensitivity analysis for multivariate analysis of Simpson index via excluding a study at a time and then pooling the remaining studies. (E) Funnel plot for assessing potential publication bias in the Simpson index before applying the trim-and-fill method. (F) Funnel plot for assessing potential publication bias in the Simpson index after applying the trim-and-fill method. *B. infantis*, *Bifidobacterium infantis*; *L. acidophilus*, *Lactobacillus acidophilus*; LOESS, locally estimated scatterplot smoothing; *L. reuteri*, *Limosilactobacillus reuteri*; *L. rhamnosus*, *Lacticaseibacillus rhamnosus*; PMW, post-menstrual week; SMD, standardised mean difference.

**Supplemental Figure 3.** Probiotic exposures and gut microbial diversity in preterm infants. (A) LOESS fits (95% CIs shaded in grey) over time for Richness index. (B) Forest plots of the association between probiotics and Richness index. (C) Subgroup analysis of Richness index between probiotics and control preterm infants by probiotic types. (D) Sensitivity analysis for multivariate analysis of Richness index via excluding a study at a time and then pooling the remaining studies. (E) Funnel plot for assessing potential publication bias in the Richness index before applying the trim-and-fill method. (F) Funnel plot for assessing potential publication bias in the Richness index after applying the trim-and-fill method. *B. infantis*, *Bifidobacterium infantis*; *L. acidophilus*, *Lactobacillus acidophilus*; LOESS, locally estimated scatterplot smoothing; *L. reuteri*, *Limosilactobacillus reuteri*; *L. rhamnosus*, *Lacticaseibacillus rhamnosus*; PMW, post-menstrual week; SMD, standardised mean difference.

**Supplemental Figure 4.** Probiotic exposures and gut microbial diversity in preterm infants. (A) LOESS fits (95% CIs shaded in grey) over time for Chao1 index. (B) Forest plots of the association between probiotics and Chao1 index. (C) Subgroup analysis of Chao1 index between probiotics and control preterm infants by probiotic types. (D) Sensitivity analysis for multivariate analysis of Chao1 index via excluding a study at a time and then pooling the remaining studies. (E) Funnel plot for assessing potential publication bias in the Chao1 index before applying the trim-and-fill method. (F) Funnel plot for assessing potential publication bias in the Chao1 index after applying the trim-and-fill method. *B. infantis*, *Bifidobacterium infantis*; *L. acidophilus*, *Lactobacillus acidophilus*; LOESS, locally estimated scatterplot smoothing; *L. reuteri*, *Limosilactobacillus reuteri*; *L. rhamnosus*, *Lacticaseibacillus rhamnosus*; PMW, post-menstrual week; SMD, standardised mean difference.

**Supplemental Figure 5.** The association between probiotics and publication bias of gut microbial diversity in preterm infants. Publication bias of (A) Shannon indexes, (B) Simpson indexes, (C) Richness indexes, (D) Chao1 indexes in probiotic and control preterm infants in each study after application of the trim-and-fill method, and the pooled effects across five included studies (meta-analysis) with 95% CI. SMD, standardised mean difference.

**Supplemental Figure 6.** Probiotic exposures and gut microbiome structure in preterm infants. (A) Scree plot visualising the percentage of variation explained by each principal component. (B) UMAP dimensionality reduction analysis of the top 13 PCs identified by the scree plot. umap, uniform manifold approximation and projection.

**Supplemental Figure 7.** Relative abundances of the dominant taxa at the phylum level in three independent cohorts. For display and clarity, only the top 10 most abundant taxa are shown. *B. infantis*, *Bifidobacterium infantis*; *L. acidophilus*, *Lactobacillus acidophilus*; *L. reuteri*, *Limosilactobacillus reuteri*; *L. rhamnosus*, *Lacticaseibacillus rhamnosus*; PMW, post-menstrual week.

**Supplemental Figure 8.** Relative abundances of the dominant taxa at the family level in three independent cohorts. For display and clarity, only the top 10 most abundant taxa are shown. *B. infantis*, *Bifidobacterium infantis*; *L. acidophilus*, *Lactobacillus acidophilus*; *L. reuteri*, *Limosilactobacillus reuteri*; *L. rhamnosus*, *Lacticaseibacillus rhamnosus*; PMW, post-menstrual week.

**Supplemental Figure 9.** Relative abundances of the dominant taxa at the genus level in three independent cohorts. For display and clarity, only the top 10 most abundant taxa are shown. *B. infantis*, *Bifidobacterium infantis*; *L. acidophilus*, *Lactobacillus acidophilus*; *L. reuteri*, *Limosilactobacillus reuteri*; *L. rhamnosus*, *Lacticaseibacillus rhamnosus*; PMW, post-menstrual week.

**Supplemental Figure 10.** Probiotic exposures and gut bacterial MetaCyc pathway abundances in preterm infants. (A) Heatmap of all samples (n = 1437) showing the 347 metabolic pathways with significant changes related to probiotics use in each cohort and meta-analysis. (B) Effect sizes were calculated by SMD in CLR using a random-effect meta-analysis method. Differential abundances of metabolic pathways detected in at least 5% of the samples in that study was corrected for FDR using the Kruskal-Wallis test. *B. infantis*, *Bifidobacterium infantis*; CLR, centered log-ratio; FDR, false discovery rate; *L. acidophilus*, *Lactobacillus acidophilus*; *L. reuteri*, *Limosilactobacillus reuteri*; *L. rhamnosus*, *Lacticaseibacillus rhamnosus*.
